# Characterisation of infection associated microRNA and protein cargo in extracellular vesicles of *Theileria annulata* infected leukocytes

**DOI:** 10.1111/cmi.12969

**Published:** 2018-11-09

**Authors:** Victoria Gillan, Deborah M. Simpson, Jane Kinnaird, Kirsty Maitland, Brian Shiels, Eileen Devaney

**Affiliations:** ^1^ Institute of Biodiversity, Animal Health and Comparative Medicine University of Glasgow Glasgow UK; ^2^ Institute of Integrative Biology, Centre for Proteome Research University of Liverpool Liverpool UK

## Abstract

The protozoan parasites *Theileria annulata* and Theileria parva are unique amongst intracellular eukaryotic pathogens as they induce a transformation‐like phenotype in their bovine host cell. T. annulata causes tropical theileriosis, which is frequently fatal, with infected leukocytes becoming metastatic and forming foci in multiple organs resulting in destruction of the lymphoid system. Exosomes, a subset of extracellular vesicles (EV), are critical in metastatic progression in many cancers. Here, we characterised the cargo of EV from a control bovine lymphosarcoma cell line (BL20) and BL20 infected with T. annulata (TBL20) by comparative mass spectrometry and microRNA (miRNA) profiling (data available via ProteomeXchange, identifier PXD010713 and NCBI GEO, accession number GSE118456, respectively). Ingenuity pathway analysis that many infection‐associated proteins essential to migration and extracellular matrix digestion were upregulated in EV from TBL20 cells compared with BL20 controls. An altered repertoire of host miRNA, many with known roles in tumour and/or infection biology, was also observed. Focusing on the tumour suppressor miRNA, *bta‐miR‐181a* and *bta‐miR‐181b*, we identified putative messenger RNA targets and confirmed the interaction of *bta‐miR181a* with ICAM‐1. We propose that EV and their miRNA cargo play an important role in the manipulation of the host cell phenotype and the pathobiology of *Theileria* infection.

## 1 INTRODUCTION

The tick‐borne parasites of ruminants *Theileria annulata* and Theileria parva are responsible for significant pathology, productivity, and economic loss over large areas of the old world. T. annulata causes tropical theileriosis, and is widespread in tropical and subtropical regions, including parts of Southern Europe, where infection rates of approximately 30% have been recorded (Gomes et al., 2016). In North Africa and India, T. annulata is the primary tick‐borne infection of cattle with an estimated 40 million cattle at risk of infection, compromising the livelihood of many small‐scale farmers and costing the economy an estimated $348 million per annum. In sub‐Saharan Africa, T. parva is the causative agent of east coast fever, a condition that is frequently fatal. These *Theileria* spp are unique amongst apicomplexan parasites because of their ability to transform host cells, which, in the case of T. annulata, are myeloid cells, dendritic cells (DC), and B cells. Following invasion by the sporozoite and development of the macroschizont stage, the infected host cell undergoes a phase of uncontrolled proliferation and metastasis, characteristics with similarities to cancer cells. Previous studies have shown that the constitutive activation of genes controlled by key transcription factors, NFkB and AP1, is critical for cellular transformation (as reviewed in Shiels et al., 2006) and proliferating *Theileria*‐infected cells are resistant to apoptosis (Heussler et al., 1999).

Understanding the molecular mechanisms underlying these events may offer novel avenues for intervention, as current control of theileriosis is problematic. In some countries, cattle are vaccinated with T. annulata‐infected cells, attenuated by prolonged *in vitro* passage (Ali et al., 2008), but these vaccines carry the continued risk of reversion to virulence and are difficult to produce and deliver. Consequently, they are not widely deployed in endemic regions. Limiting the spread of infection is possible using externally applied acaricides against the vector, but resistance to these chemicals compromises control (Abbas, Zaman, Colwell, Gilleard, & Iqbal, 2014) and their use has environmental implications. *Theileria* infection can be controlled by chemotherapy, primarily using buparvaquone, but just as with tick control, drug resistance has been identified (Mhadhbi, Chaouch, Ajroud, Darghouth, & BenAbderrazak, 2015), and the cost of treatment is relatively high. Thus, to improve productivity in endemic regions, development of novel treatments and vaccines is required, a challenge that requires greater understanding of the molecular mechanisms involved in parasite manipulation of the host cell. The potential socio‐economic impact of improved control of T. parva was illustrated in a recent study of pastoralist households in Kenya. Uptake of a vaccine was predicted to be positively associated with increased household income and expenditure on food and had a significant effect on the likelihood of children attending school (Marsh, Yoder, Deboch, McElwain, & Palmer, 2016).

In this paper, we adopted a novel approach to understanding the molecular mechanisms underlying the pathogenesis of T. annulata, focusing on extracellular vesicles (EV) and their microRNA (miRNA) cargo. EV have important roles in cell–cell communication and have been particularly well characterised in cancer because of their role in various processes leading to oncogenesis, including their capacity to generate a metastatic niche, which allows the engraftment of tumour cells elsewhere in the body (Costa‐Silva et al., 2015; Hoshino et al., 2015). The best‐described subset of EV are exosomes, which derive from the endocytic pathway, display a characteristic cup‐shaped morphology and are approximately 50–150 nm in size. Other classes of EV, such as microvesicles bud from the plasma membrane, while apoptotic bodies occur when cells are undergoing apoptotic fragmentation. These subtypes have a larger size range (50 to 2000 nm and 50 to 5000 nm, respectively) and lack the cup‐shape morphology unique to exosomes (reviewed in Edgar, 2016). Despite these differences, distinguishing exosomes from other EV is complex, and there is no single reliable marker for their identification. Consequently, in this manuscript, we use the terminology EV throughout. EV can contain protein and nucleic acids, such as miRNA or messenger RNA (mRNA). Depending on the cell type from which they are derived, their role in cellular communication can vary, relating to the complex and dynamic nature of their cargo.

EV are gaining prominence in infectious disease biology, with several recent studies highlighting their importance in both pathology and the modulation of host immune responses (reviewed in Robbins and Morelli, 2014). For example, EV from *Trypanosomes* have been implicated in the development of anaemia (Szempruch et al., 2016), while EV from *Plasmodium*‐infected red blood cells are important in malaria pathogenesis (Mantel et al., 2016). EV are also involved in the initiation and/or regulation of immune responses: They can mediate the transfer of antigenic peptides from infected cells to antigen‐presenting cells and can deliver either stimulatory or suppressive signals to regulate the induction and differentiation of immune responses (reviewed in Robbins and Morelli, 2014). Tumour‐derived EV are most often associated with immune suppression, while EV from *Plasmodium*‐infected reticulocytes induce protection via their interaction with CD4 and CD8 cells (Martin‐Jaular et al., 2016). Notably, many of the properties of EV are mediated via their miRNA cargo (Mantel et al., 2016).

Here, we compared the protein and miRNA profile of EV from T. annulata infected (TBL20) and control uninfected cells (BL20). Proteomic analysis revealed that a large number of host proteins were elevated in infected relative to uninfected EV. The expression of several miRNAs was also shown to be dysregulated in EV from TBL20 cells (TBL20‐EV) compared with EV from BL20 cells (BL20‐EV). While each of the differentially expressed miRNA have known roles in regulating aspects of the immune response or in oncogenesis, we focus on the tumour suppressor miRNA, *bta‐miR‐181a/b* and their interaction with potential target molecules. Our results indicate that infection‐associated EV proteins and miRNA may play a key role in the pathobiology of this important veterinary pathogen.

## 2 RESULTS

### 2.1 Identification of EV from BL20 and TBL20 cells

**Figure 1:**
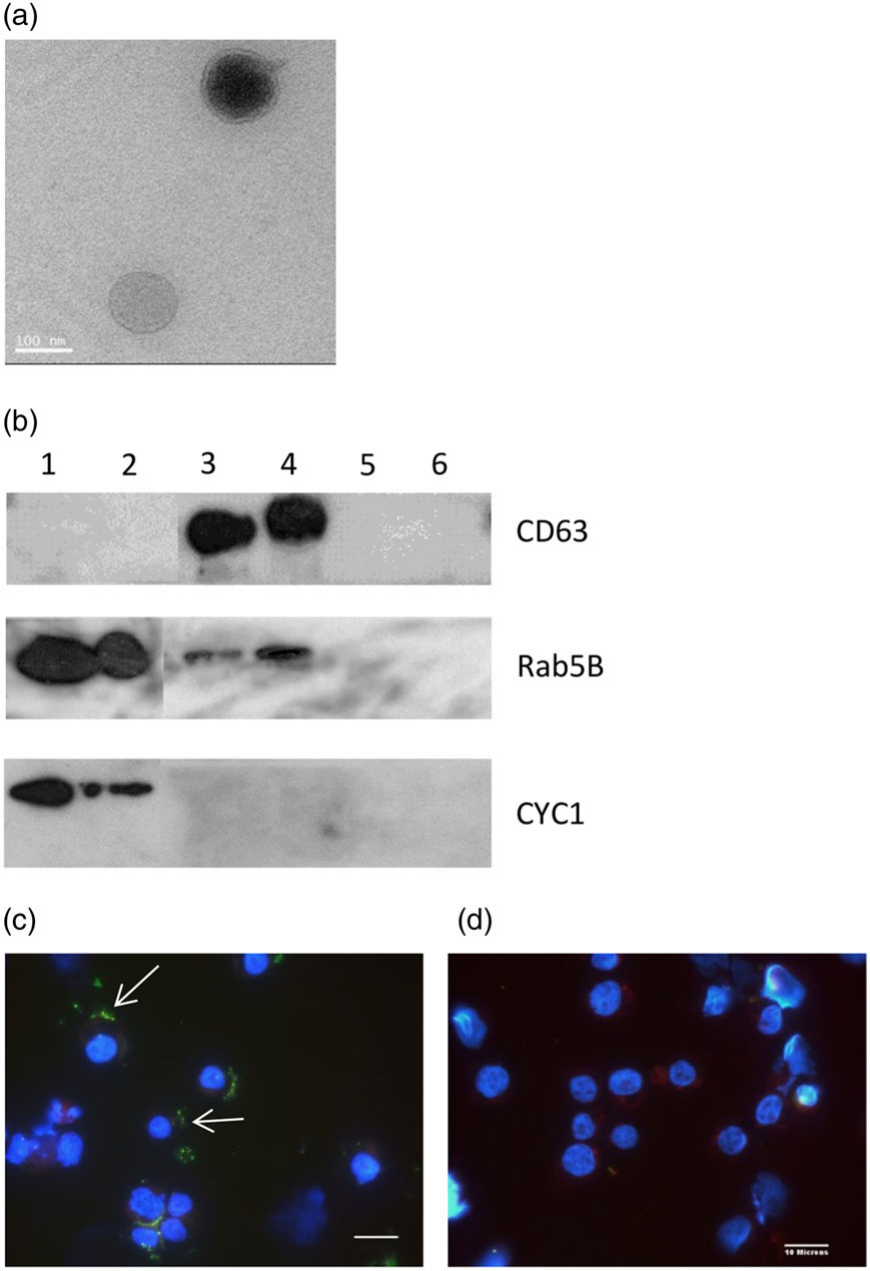
EV shed by BL20 and TBL20 cells. (a) Whole mount transmission electron microscopy of purified exosomes from TBL20. Scale bar represents 100 nm. (b) Western blot analysis of 1. BL20 whole cell extract; 2. TBL20 whole cell extract; 3. BL‐EV (ultracentrifugation); 4. TBL‐EV (ultracentrifugation); 5. BL20 concentrated EV depleted supernatant; 6. TBL20 concentrated EV depleted supernatant. Antibodies for mouse antibovine CD63 (1:500, AbD Serotec), rabbit antihuman Rab 5B (1:500, Santa Cruz Biotechnology), and rabbit antibovine Cyc1 (1:1000, Aviva Systems Biology) were used. Fluorescent microscopy images at ×60 magnification of DAPI stained BL20 cells incubated with (c) PKH‐67 labelled TBL20‐EV and (d**)** EV depleted supernatant from TBL20 cultures. Green, EV (white arrows) and blue, DAPI stained nuclei. Scale bars represent 10 μm

BL20‐EV and TBL20‐EV were characterised using a variety of methods. Electron microscopy revealed vesicles of the correct size for exosomes (~100 nm) in the preparations from both BL20 and TBL20 culture supernatants using ultracentrifugation (Figure 1a). Western blots showed that CD63 was highly enriched in BL20‐EV and TBL20‐EV samples and virtually absent in the corresponding whole cell extracts and EV depleted supernatant. Rab‐5B was present in EV preparations and cell extracts but absent in EV depleted supernatants. CYC1, a mitochondrial associated protein, was used as a negative control and was present in cell extracts but absent in EV preparations and EV depleted supernatants as would be expected (Figure 1b). Quantification by either NTA or EXOCET yielded 5 × 10^8^ to 1 × 10^9^ EV per million cells. Additionally, to examine whether TBL20‐EV have the capacity to enter other cells, PKH‐67 labelled EV were incubated with BL20 cells for 2 hr and examined using fluorescence microscopy. Figure 1c shows a representative image demonstrating the presence of labelled EV within the recipient cells. When EV depleted supernatants were labelled using the same protocol and included as a negative control, no intracellular particles were detected (Figure 1d).

### 2.2 BL20‐EV and TBL20‐EV exhibit differences in protein composition/cargo

Proteomic analysis of both TBL20‐EV and BL20‐EV samples generated a list of 1,068 quantifiable proteins from Progenesis. These data have been deposited to the ProteomeXchange Consortium via the PRIDE (Vizcaino et al., 2016) partner repository with the dataset identifier PXD010713 and 10.6019/PXD010713. Prior to differential expression analysis, this list of 1,068 proteins was searched for the top 100 proteins in the Exocarta database of exosome‐associated molecules (http://www.exocarta.org/exosome_markers). Seventy of these key exosome markers were present in both TBL20‐EV and BL20‐EV populations, consistent with the isolation of a population of EV highly enriched with exosomes. Table S2 lists the 100 exosome markers and gives their normalised abundance in TBL20‐EV and BL20‐EV.

**Figure 2:**
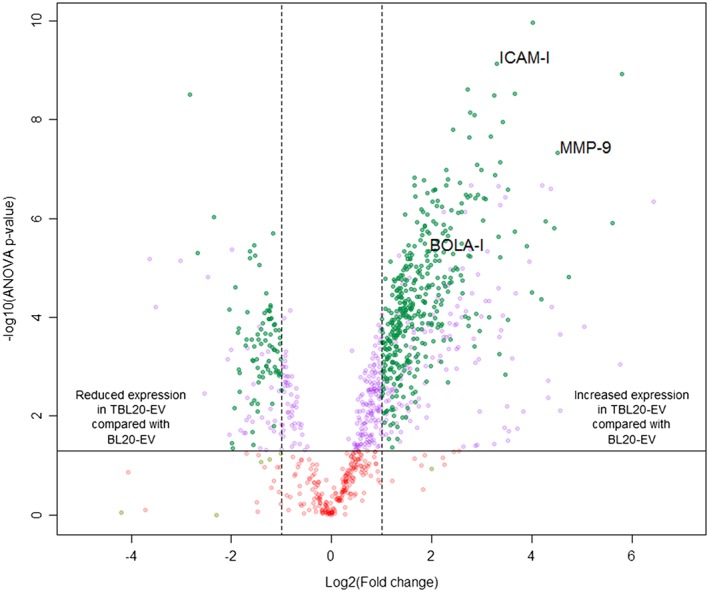
Volcano plot depicts differences in protein cargo in TBL20‐EV vs. BL20‐EV. All proteins detected by Progenesis QI were plotted. Each circle represents one protein. The log_2_ fold change in the TBL20‐EV vs. BL20‐EV is represented on the x‐axis. The y‐axis shows the negative log_10_ of the ANOVA *p* value. Different colours of circles represent proteins with the following: red, ANOVA *p* > 0.05; purple, ANOVA *p* ≤ 0.05; dark green, ANOVA *p* ≤ 0.05, ≥2 unique peptides and ≥2 fold change; pale green, ≥2 unique peptides and ≥2 fold change. ICAM‐1, MMP‐9, and BOLA‐1 are annotated to illustrate their levels of significance. All proteins above the solid horizontal line are significantly different between the groups, with ANOVA *p* ≤ 0.05. The top left panel contains proteins, which are reduced in TBL20‐EV compared with BL20‐EV, and the top right panel contains proteins, which are increased in TBL20‐EV compared with BL20‐EV, only those in dark green were used in downstream analysis

**Figure 3:**
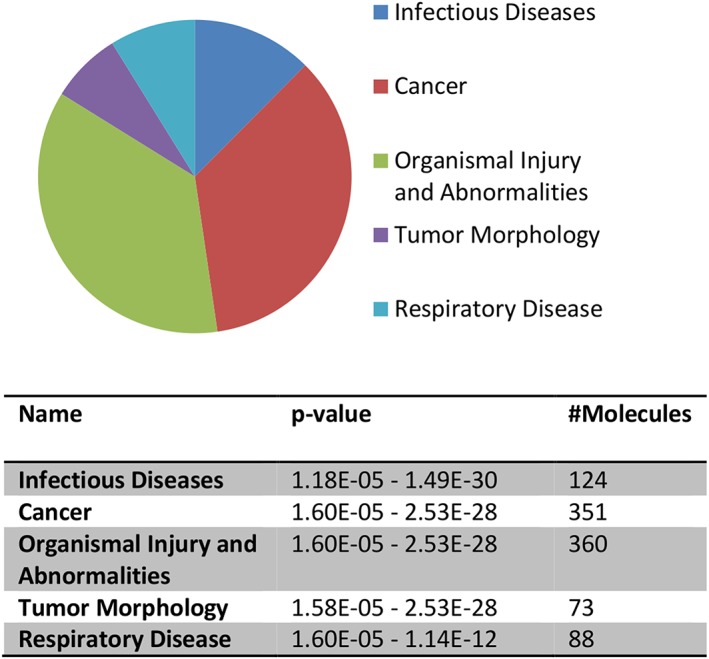
Ingenuity pathway analysis (Qiagen) of EV proteomic data. Three hundred and ninety‐five proteins significantly upregulated in TBL20‐EV, as listed in Table S3, were used for these analyses. The top five molecular and cellular functions and canonical pathways are shown in the pie chart and table. Threshold criteria considered for the analysis are ANOVA *p* ≤ 0.05, ≥2 unique peptides, and ≥2 fold change

While both populations of EV contained proteins in common, differential expression analysis showed a clear divergence between BL20‐EV and TBL20‐EV, with no outliers at ANOVA *p* ≤ 0.05 at either the peptide or protein level (Figure S1 represents PCA biplots of individual samples). To increase the confidence of downstream analysis, in addition to statistical significance (ANOVA *p* ≤ 0.05), only those proteins identified by two or more unique peptides and exhibiting a fold change ≥2 were selected, resulting in a total of 479 significantly differentially expressed proteins. Of these, 395 were elevated in TBL20‐EV compared with BL20‐EV, whereas 84 were elevated in BL20‐EV compared with TBL20‐EV (depicted in Figure 2 and listed Table S3). Proteins up‐regulated in TBL20‐EV included many previously identified in TBL20 cells (Kinnaird et al., 2013a) with likely functions in extracellular matrix degradation (e.g., MMP‐9), and several involved in the immune response (e.g., ICAM‐1 and BOLA‐1). These results demonstrate that the presence of the parasite alters host protein expression levels in EV, as well as in host cells. In order to cluster these proteins into functional networks, the datasets were uploaded to ingenuity pathway analysis (IPA). Of the 395 proteins elevated in TBL20‐EV, the most significant biological processes were “infectious disease” and “cancer.” The pie chart in Figure 3 represents the top five enrichment terms generated by IPA for up‐regulated proteins in TBL20‐EV. These pathways are ranked in order of significance as shown in the table in Figure 3.

### 2.3 BL20‐EV and TBL20‐EV exhibit differences in miRNA cargo

**Figure 4:**
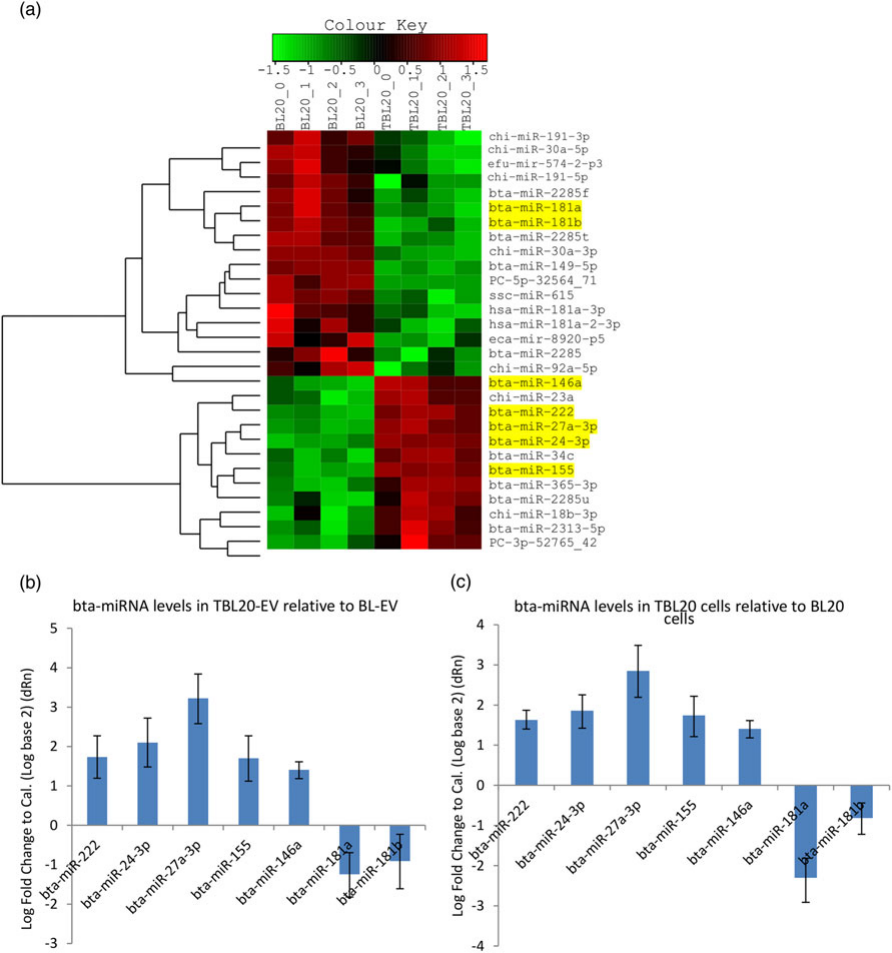
miRNA profile of TBL20‐EV. (a) Differentially expressed miRNA determined by RNA‐Seq are depicted in a heat map *p* ≤ 0.01. (b) Histograms depicting comparative qRT‐PCR data from biological replicates of EV confirms RNA‐Seq data and (c) cell of origin samples show a similar profile. Only miRNA with *p* ≤ 0.01 and a read count >500 were selected for analysis by qRT‐PCR. log_2_ fold change is calculated by comparing test samples (TBL20‐EV) to calibrator samples (BL20‐EV) using the constitutively expressed miRNA *bta‐miR‐30e‐3p* as an internal normaliser

The results of miRNA profiling of BL20‐EV and TBL20‐EV showed that the expression of 15 miRNA differed significantly between the groups at *p* < 0.01, and 39 at *p* < 0.05 (data available at NCBI GEO, accession number GSE118456). In order to select miRNA of interest, we applied a cut‐off of *p* < 0.01 and a read count of >500, which gave a list of six miRNA, each of which has known functions in various models of malignancy and/or infectious disease. These results are shown in Figure 4a, where the heat map depicts the level of differential expression between samples, and in Table 1, in which log_2_ fold changes are detailed. *bta‐miR‐222*, *bta‐miR‐24‐3p*, *bta‐miR‐27a‐3p*, *bta‐miR‐155*, and *bta‐miR‐146a* are all enriched in TBL20‐EV compared with BL20‐EV, while *bta‐miR‐181a* and *b* showed a contrasting profile and are downregulated in TBL20‐EV compared with BL20‐EV. Each of the upregulated miRNA are associated with tumours and promote proliferation (see Table 1), while *bta‐miR‐181a/b* can act as tumour suppressors (Huang, Ye, Yang, Shi, & Zhao, 2015; Wang et al., 2015; Weng, Lal, Yang, & Chen, 2015). The differential expression revealed by RNA‐Seq analysis was confirmed via qRT‐PCR for the six candidate miRNA on separate biological replicates of BL20‐EV and TBL20‐EV (Figure 4b). In addition, miRNA qRT‐PCR was carried out on BL20 and TBL20 cells to determine whether specific miRNA might be selectively packaged into EV. However, the cellular expression pattern of all six miRNA was similar to that observed in EV (Figure 4c). We also characterised the expression of all six miRNA in a different cell line infected with *Theileria*, TBL‐3 compared with its uninfected partner BL‐3. All miRNA showed a similar pattern of expression in EV isolated from TBL‐3 cells relative to BL‐3 cells; in whole cell extracts, only *bta‐miR‐155* was different (Figure S2). In subsequent experiments, we focused our efforts on defining the function of the tumour suppressor miRNA, *bta‐miR‐181a/b* in *Theileria* infected cells.

**Table 1:** Host miRNA differentially expressed in TBL20‐EV compared with BL20‐EV

microRNA	Log_2_ (*p value*)	Function
*bta‐miR‐222*	1.63 (*p =* 1.5E‐05)	Oncogene in many human malignancies (Felicetti et al., 2016; Kara et al., 2015; Li et al., 2016; M. Wang et al., 2016)
*bta‐miR‐24‐3p*	2.66 (*p =* 1.5E‐05)	Promotes cell proliferation and inhibits apoptosis in human breast cancer (Lu et al., 2015)
*bta‐miR‐27a‐3p*	3.39 (*p =* 3.3E‐05)	Promotes cellular proliferation (Kara et al., 2015; Nakata et al., 2015; Wu et al., 2015)
*bta‐miR‐155*	2.94 (*p =* 9.3E‐04)	Tumour associated miRNA (Faraoni, Antonetti, Cardone, & Bonmassar, 2009; Jurkovicova et al., 2014; Kara et al., 2015)
*bta‐miR‐146a*	2.76 (*p =* 4.3E‐05)	Tumour associated miRNA (Petrovic, Davidovic, Bajic, Obradovic, & Isenovic, 2017; Sun et al., 2015)
*bta‐miR‐181a/b*	−1.35/−1.05 (*p =* 8E‐05/2E‐05)	Tumour suppressors (Shi et al., 2008; H. Wang et al., 2015; Weng et al., 2015)

*Note*. miRNA: micro RNA; TBL20‐EV: extracellular vesicles from *Theileria annulata* infected line; BL20‐EV: extracellular vesicles from bovine lymphoblastoid cell line.

### 2.4 Computational analysis of *bta‐miR‐181a* and *b* mRNA targets

Reduced levels of *bta‐miR‐181a* and *bta‐miR‐181b* in TBL20 cells may result in higher levels of expression of their target mRNA and increased levels of their protein products in EV. As miRNA bind to partially complementary sequences usually within the 3′ UTR of their target mRNAs, these targets can be predicted computationally (Rajewsky, 2006; Ritchie & Rasko, 2014). The 3′ UTR of the genes encoding the top 15 upregulated proteins in TBL20‐EV were analysed for *bta‐miR‐181a* and *bta‐miR‐181b* binding sites. *bta‐miR‐181a* and *bta‐miR‐181b* sequences were downloaded from miRBase release 21 (http://www.mirbase.org/; Kozomara & Griffiths‐Jones, 2014) and have identical seed sequences; however, their secondary structure predicted by folding and hybridisation software Mfold (http://unafold.rna.albany.edu; Zuker, 2003) is different (Table S4). For this reason, *bta‐miR‐181a* and *bta‐miR‐181b* targets were analysed separately. High confidence potential mRNA targets were defined by a ddG score of <−7 defined by the PITA algorithm and are highlighted in bold (Table 2). Amongst these were vesicle‐associated proteins TSN‐6 and IST‐1 but of particular interest was ICAM‐1, which interacts with leukocyte function associated antigen‐1, an interaction that contributes to the migration of leukocytes across the endothelium (Gahmberg, Tolvanen, & Kotovuori, 1997; Hogg, Laschinger, Giles, & McDowall, 2003). Previous studies have shown that ICAM‐1 mRNA is upregulated in TBL20 cells (Durrani, Weir, Pillai, Kinnaird, & Shiels, 2012). Additionally, matrix metalloproteinase 9 (MMP‐9) was significantly overexpressed in TBL20‐EV compared with BL20‐EV and shown to be a potential target of *miR‐181a* and *b*. MMP‐9 is upregulated at the “pre‐metastatic niche” in lung metastasis (Kaplan et al., 2005), and various MMPs have been shown to be present in tumour cell EV (Dolo et al., 1999; Hakulinen, Sankkila, Sugiyama, Lehti, & Keski‐Oja, 2008) and to have a role in metastasis of TBL20 cells (Adamson, Logan, Kinnaird, Langsley, & Hall, 2000). Although significantly altered in TBL20‐EV (but not in the top 15 differentially expressed proteins), BOLA‐1 (ranked 83) was also included as a possible target of *bta‐miR‐181a* and *b*, due to its role in antigen presentation to CD8+ T cells, a major effector mechanism of protective immunity against theileriosis (Shaw, Tilney, Musoke, & Teale, 1995).

**Table 2:** The top 15 upregulated proteins in TBL20‐EV compared with BL20‐EV and predicted *bta‐miR‐181a* and *bta‐miR‐181b* binding sites

Gene	Fold change (up in TBL20‐EV)	*q* value	No. of miR‐181a binding sites (combined ddG)	No. of miR‐181b binding sites (combined ddG)
VTA1_BOVIN Vacuolar protein sorting‐associated protein^b^	16.5	3.13E‐08	4(−2.99)	3 (−5.37)
ICAM‐1_BOVIN Intercellular adhesion molecule 1	9.6	4.96E‐08	**2 (−9.01)**	0
NEUM_BOVIN Neuromodulin	56.2	7.06E‐08	1 (2.51)	1 (−4.86)
TSN6_BOVIN Tetraspanin‐6^b^	12.6	8.72E‐08	1 (−6)	**1 (−9)**
VPS4B_BOVIN Vacuolar protein sorting‐associated protein 4B^b^	9.5	8.72E‐08	2 (−4.88)	2 (−6.32)
MFGM_BOVIN Lactadherin	6.6	8.72E‐08	0	0
SATT_BOVIN Neutral amino acid transporter A	7.3	1.85E‐07	0	1 (−6)
ITA5_BOVIN Integrin alpha‐5	6.1	2.02E‐07	0	1 (0.7)
IST1_BOVIN IST1 homologue^b^	10.5	2.58E‐07	1 (2)	**3 (−9)**
TMEDA_BOVIN Transmembrane emp24 domain‐containing protein 10^b^	5.4	2.58E‐07	3 (−4.51)	3 (−4.1)
VPS28_BOVIN Vacuolar protein sorting‐associated protein 28 homologue^b^	6.8	2.91E‐07	3 (−4.13)	1 (0.51)
CHM1B_BOVIN Charged multivesicular body protein 1b^b^	9	3.00E‐07	2 (0.2)	3 (5.58)
MMP‐9_BOVIN Matrix metalloproteinase‐9	23.3	9.12E‐07	**1 (−13.05)**	**1 (−11.84)**
VAPA_BOVIN Vesicle‐associated membrane protein‐associated protein A^b^	10.4	9.12E‐07	2 (−4.76)	2 (−5.83)
MPP7_BOVIN MAGUK p55 Subfamily Member 7	7.5	9.12E‐07	4 (−5.69)	**2 (−8.05)**
aHA1A_BOVIN BOLA‐1 Class I histocompatibility antigen, alpha chain	3.8	8.47E‐06	**2 (−11.95)**	**2 (−14.5)**

*Note*. TBL20‐EV: extracellular vesicles from *Theileria annulata* infected line; BL20‐EV: extracellular vesicles from bovine lymphoblastoid cell line.

a BOLA‐1 also included.

b known components of EV.

### 2.5 Functional confirmation of *bta‐miR‐181a*/*b* binding to target mRNAs

**Figure 5:**
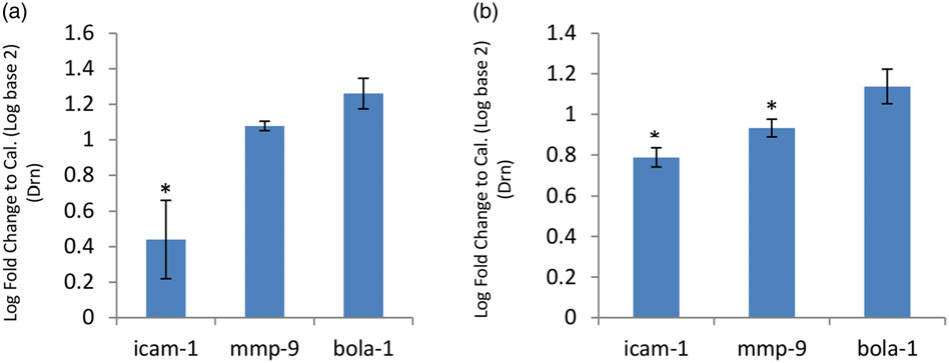
*bta‐miR‐181a i*nteracts with predicted target gene 3′ UTR. Histograms show the mean of three experiments +/− standard deviation comparing Firefly to Renilla luciferase signal in the presence of *bta‐miR‐181a* (a) or *bta‐miR‐181b* (b) and the 3′ UTRs of predicted targets mRNAs, ICAM‐1*,* MMP‐9, and BOLA‐1. *bta‐miR‐181a* or *bta‐miR‐181b* were cloned in the forward orientation and reverse orientation, and reduction in signal was calculated by comparison of the two (*p ≤ 0.05)

Three predicted targets of *bta‐miR‐181a or b* from the bioinformatic analysis were further investigated by transfection of HEK293 cells in a dual luciferase assay. Approximately 1 kb of the ICAM‐1, MMP‐9, AND BOLA‐1 3′ UTRs were cloned from TBL20 genomic DNA downstream of firefly luciferase and transiently transfected into HEK293 cells along with a plasmid containing *bta‐miR‐181a* or *b* in the forward or reverse direction. ICAM‐1 and BOLA‐1 3′ UTR sequence both contained two putative *bta‐miR‐181a* binding sites within their 3′ UTR, while MMP‐9 3′ UTR contained a single site. For *bta‐miR‐181b*, the BOLA‐1 3′ UTR contained two binding sites, MMP‐9 3′ UTR contained one, and ICAM‐1 3′ UTR had no predicted binding sites (see Table 2). Levels of luciferase in the presence of the forward or reverse *bta‐miR‐181a* or *b* were compared. *bta‐miR‐181a* suppressed the expression of ICAM‐1 3′ UTR fused to luciferase by 66% (*p =* 0.00095), while no difference was observed for MMP‐9 or BOLA‐1 3′ UTR (Figure 5a). For *bta‐miR‐181b*, the suppression of both ICAM‐1 and MMP‐9 constructs was more moderate, 21% and 7%, respectively, but these results were also significant (*p =* 0.047 and 0.021, respectively, Figure 5b). Transfection of both *bta‐miR‐181a* and *b* together did not enhance the reduction in luciferase (data not shown). Unexpectedly, a small, but significant increase in BOLA‐1 expression was observed in the presence of *bta‐miR‐181a* or *b* (*p =* 0.0003 and 0.0185, respectively).

### 2.6 Overexpression of *miR‐181a* reduces ICAM‐1 mRNA expression in TBL20 cells

**Figure 6:**
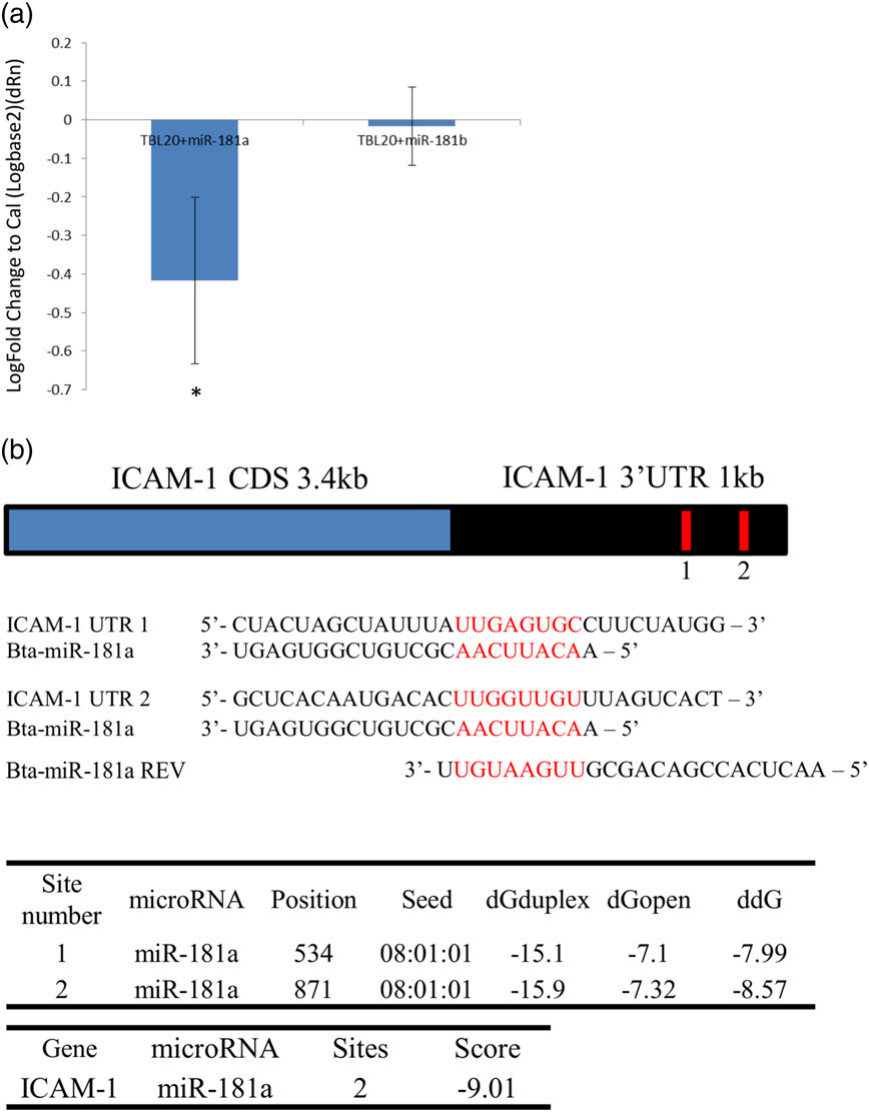
Overexpression of *miR‐181a* negatively regulates ICAM‐1 mRNA. (a) Comparative qRT‐PCR results show significantly reduced levels of ICAM‐1 expression in TBL20 cells when they are transfected with *miR‐181a* mimic (Dharmacon; *p ≤ 0.05). These results were calibrated against ICAM‐1 expression inTBL20 cells transfected with *cel‐miR‐67* negative control (Dharmacon). *bta‐miR‐30e‐3p* was used as an internal normaliser. These results were not replicated when TBL20 cells were transfected with *miR‐181b* mimic. (b) Schematic representation of ICAM‐1 cDNA and 3′ UTR showing the conserved *miR‐181a* seed region in the 3′ UTR. Bovine *miR‐181a* mature sequence with proposed base pairing to the 3′ UTR are presented. The reverse complement of *bta‐miR‐181* was used as a control for luciferase assays. Tables show data from generated using https://genie.weizmann.ac.il/pubs/mir07/mir07_prediction.html online prediction tool resulting in a combined ddG score for ICAM‐1 3’ UTR predicted binding to *bta‐mir‐181a*.

Along with elevated protein expression, mRNA encoding ICAM‐1, MMP‐9, and BOLA‐1 expression levels were increased in TBL20 cells compared with BL20 cells (Figure S3). Therefore, it was of interest to determine if there was a correlation between levels of *bta‐miR‐181a* or *bta‐miR‐181b* and their target genes in infected cells. To this end, synthetic mimics of *miR‐181a* and *b* or a negative transfection control *cel‐miR‐67* (miRIDIAN Mimic Negative Control #1) were introduced to TBL20 cells by nucleofection. Following transfection of TBL20 cells, *bta‐miR‐181a* and *b* mimics, were detected by qRT‐PCR while the *cel‐miR‐67* control showed no effect on levels of *mir‐181a* or *b* (Figure S4). qRT‐PCR was also carried out to determine if levels of ICAM‐1, MMP‐9, or BOLA‐1 mRNA were inhibited by the mimics. Interestingly, ICAM‐1 was significantly downregulated only in the presence of *bta‐miR‐181a* (Figure 6a), validating the results from the dual luciferase assay where *bta‐miR‐181a* had a much more pronounced effect on ICAM‐1 than *bta‐miR‐181b*. Expression of MMP‐9 and BOLA‐1 mRNA were unaffected by the presence of *bta‐miR‐181a* and *b* mimics (data not shown); therefore, these mRNAs are unlikely to be true targets of *miR‐181a/b* in his system.

### 2.7 Transfection of TBL20 cells with *miR‐181a* causes a significant growth phenotype

**Figure 7:**
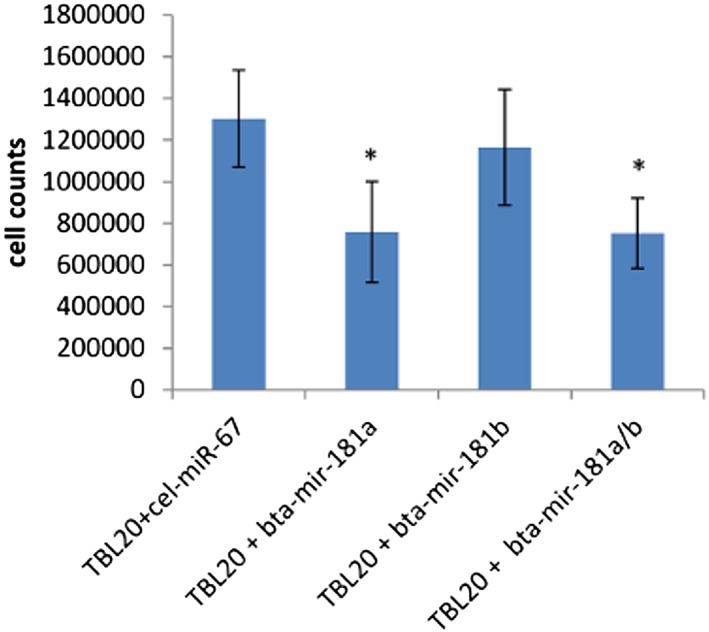
*miR‐181a* overexpression affects cell number in TBL20 cultures. Graph shows the mean of three experiments where cell counts from TBL20 cells transfected with *cel‐miR‐67, miR‐181a* mimic, *miR‐181b* mimic, or both mimics were taken at 24 hr. Counts from wells containing TBL20 cells expressing *miR‐181a* mimic or both mimics consistently and significantly showed decreased numbers (*p ≤ 0.05)

When BL20 and TBL20 cells were transfected with *miR‐181a, b*, or both mimics, we observed a significant reduction in growth of TBL20 cells in the presence of *miR‐181a* compared with cells transfected with *cel‐miR‐67*. This effect was not observed using *miR‐181b*, and simultaneous transfection of both *miR‐181a* and *b* showed no additive effect (Figure 7). Significantly, this growth phenotype was only observed in TBL20 cells and not in BL20 cells. To further understand a possible role for *miR‐181a* in this system, IPA was used to generate a list of predicted *bta‐miR‐181a* gene targets. Selecting only moderate and high confidence predictions, 858 potential target genes were used in pathway analysis, and the top four canonical pathways were ceramide degradation, PTEN signalling, T‐cell signalling, and regulation of IL‐2 expression, all of which are associated with cellular proliferation, growth, or apoptosis (Table S5). It is interesting to note that an alpha subunit of casein kinase II (CSNK2A2) is a potential target of *miR‐181a* while other alpha and beta subunits of casein kinase II (CSNK2A1/CSNK2B) are upregulated in TBL20‐EV. A previous study has identified this molecule as intrinsic to *Theileria* induced lymphocyte transformation (Dessauge, Lizundia, & Langsley, 2005). Additionally, the list of 858 *miR‐181a* potential target genes was cross‐referenced with previous BL20 vs. TBL20 microarray data from this lab (Kinnaird et al., 2013b). One thousand five hundred and ninety‐six genes, which were upregulated in TBL20 cells versus BL20 cells with an FDR (*q* value) < 0.05 from that study were included in the cross referencing. Forty‐four genes were present in both lists (as shown in Table S6), some of which have roles in the regulation of cell proliferation such as CD69, DUSP10, and FAS. Further studies will be required to confirm an association between *miR‐181a* and any of these potential targets.

## 3 DISCUSSION

The transforming species of *Theileria* are amongst the most intriguing of eukaryotic pathogens because of their unique ability to subvert host gene expression, and cause a cancer‐like phenotype in the infected host leukocyte. Most previous studies have focused on the association between the parasite and the constitutive activation of host transcription factors such as NFkB, AP1, and cMyc (Baumgartner et al., 2000; Dessauge et al., 2005). Here, we describe a potential role for EV and their cargo in the pathobiology of T. annulata infection. EV are known to mediate communication between various cell types by transmitting signals from donor to recipient cells, negating the requirement for cell‐to‐cell contact (Tkach & Thery, 2016). The subsequent reprogramming of recipient cells has provided important insights into disease progression, most notably in the process of oncogenesis (Choi et al., 2017). In our experiments, we isolated a population of vesicles enriched with exosomes from BL20 and TBL20 cells, as judged by their protein composition, high levels of expression of marker proteins and size profile. By labelling with PKH67, TBL20‐EV were shown to enter uninfected cells, demonstrating a potential means of delivering their content. These observations are particularly pertinent given the pathogenesis of T. annulata infection, where uncontrolled proliferation, metastasis, and destruction of the lymphoid system are hallmarks of acute disease. Although EV can contain a range of components including mRNA, miRNA, and lipids (Yanez‐Mo et al., 2015), we focused on the miRNA fraction because of their potential to modulate target gene expression. Furthermore, previous studies had implicated *bta‐miR‐155* in the proliferative phenotype of T. annulata cells, by acting in a positive feedback loop, which results in stabilisation of c‐Jun and increased transcription of the gene containing *bta‐miR‐155* (Marsolier et al., 2013).

By applying stringent criteria, six miRNA were shown to be differentially expressed in TBL20‐EV compared with BL20‐EV. These expression patterns were conserved in TBL20 versus BL20 cells, indicating that in this case, the EV cargo largely reflects that of the host cell. A recent publication (Haidar et al., 2018) described the miRNA of TBL20 and TBL3 cells relative to uninfected cells. In general, comparison of those data with the EV miRNA profiles reported here are very similar although there are subtle differences, possibly reflecting variable culture conditions used in the different laboratories.

Each of the differentially expressed miRNA identified in our study has known functions in either infectious disease or cancer. While most of the miRNA identified function to promote cell proliferation or act as oncogenes, we focused on two miRNA, *bta‐miR‐181a* and *b,* that act as tumour suppressors (Shi et al., 2008) and that were significantly downregulated in TBL20‐EV compared with BL20‐EV. In order to investigate the function of *bta‐miR‐181a* and *b* in TBL20 cells, putative target genes were determined using a dual approach: First, we selected proteins significantly upregulated in TBL20‐EV versus BL20‐EV and second, identified those containing binding sites for *bta‐miR‐18a* or *b* within the 3′ UTR of the corresponding mRNA. The interaction of some of these targets with *bta‐miR‐181a/b* was then studied in a dual luciferase assay.

*bta‐miR‐181 a* and *b* are closely linked on Chr XVI in an intergenic region of the bovine genome, and while the closest gene to the *bta‐miR‐181* locus is a protein tyrosine phosphatase receptor type C (CD45), there is no evidence of any interaction between the miRNA and this gene (data not shown). Despite sharing identical seed sequences and highly conserved mature sequences, differences were observed in the ability of *bta‐miR‐181a* and *bta‐miR‐181b* to bind to ICAM‐1 3’ UTR, with *bta‐miR‐181a* showing a three‐fold greater suppressive effect than *bta‐miR‐181b*. The *miR‐181* family has a well‐characterised role in modulating expression of multiple immune‐related molecules (Sun, Sit, & Feinberg, 2014) and endothelial‐expressed molecules such as VCAM (Liu et al., 2017). However, the major target of *bta‐miR‐181a* confirmed in this study was ICAM‐1, which encodes a cell surface glycoprotein expressed on a range of immune and endothelial cell types. ICAM‐1 is known to be significantly overexpressed in TBL20 cells (Kinnaird et al., 2013b) and in TBL20‐EV as reported here. By analogy with human cancer cells, overexpression of ICAM‐1 may play a role in metastasis (Yu, Lin, & Tang, 2013). As the dual luciferase assays were carried out in a heterologous transfection system, we next tested whether overexpression of either miRNA in TBL20 cells would affect target gene expression and thus indicate that the mimic was able to enter the miRNA pathway. Overexpression of *bta‐mir‐181a* in TBL20 cells resulted in the downregulation of ICAM‐1, while *bta‐mir‐181b* had no effect on its own. MMP‐9 was also experimentally confirmed as a weak target of *bta‐miR‐181b* but not of *bta‐miR‐181a* in the dual luciferase assay. However, expression of the *miR‐181b* mimic in TBL20 cells had no effect on MMP‐9 expression, implying that under the conditions tested, MMP‐9 is unlikely to be a true target of *miR‐181b*. However, the study of Haidar et al., demonstrated that a different miRNA, *bta‐miR126‐5p*, regulates MMP‐9 expression; transfection of TBL20 cells with an inhibitor of a *bta‐miR126‐5p* decreased MMP‐9 levels and reduced the capacity of the cells to migrate, while transfection with a mimic increased cell migration (Haidar et al., 2018).

TBL20 cells transfected with *miR‐181a* or *b* mimics were also examined to determine if there were phenotypic alterations that correlated with expression of either mimic. We observed that TBL20 cells proliferated much less well in the presence of *miR‐181a* mimic while *miR‐181b* alone had no such effect. Elevating *miR‐181a* suppressed proliferation and cellular growth, but only in the infected cell, consistent with the role of *miR‐181a* as a tumour suppressor (Shi et al., 2008). As the mechanism/s underlying the suppression of proliferation seemed unlikely to involve regulation of ICAM‐1, IPA was performed on all 858 predicted *bta‐mir‐181a* mRNA targets. A large number of potential pathways were highlighted, but the four most significant were ceramide degradation and PTEN, TGF‐b, and IL‐2 signalling (Table S5). Each of these pathways is associated with the control of proliferation and contain genes that may regulate proliferation of TBL20 cells. Cross‐referencing this list of predicted targets with previously published microarray data of genes upregulated in TBL20 cells revealed a list of 44 genes, some of which are involved in cellular proliferation. Future studies will focus on generating experimental data to examine the specific role played by *bta‐miR‐181a* in cell proliferation by confirming some of these interactions.

In conclusion, the miRNA profile of EV and TBL20 cells predicts their hyper‐proliferative capacity with reduction of major tumour suppressing miRNA and elevation of pro‐oncogenic miRNA. How infected cell EV impact on the pathobiology of tropical theileriosis is yet to be resolved, but it is intriguing to note that in older studies, parasite infected cells were shown to induce inappropriate activation and proliferation of naïve T‐cells via a contact and soluble factor dependent mechanism (Campbel & Spooner, 1999; Campbell, Howie, Odling, & Glass, 1995). While this phenomenon is likely to be complex, the role of EV with their altered expression of protein and miRNA, their ability to enter cells and to mediate immune responses (Robbins & Morelli, 2014) could have a profound effect on the progression of T. annulata infection.

## 4 Experimental procedures

### 4.1 Cell culture

BL20 is an immortalised bovine lymphosarcoma cell line (Morzaria, Roeder, Roberts, Chasey, & Drew, 1982), while TBL20, is a parasite‐infected cell line obtained by in vitro infection of BL20 cells with sporozoites of T. annulata (strain Hissar; Shiels, McDougall, Tait, & Brown, 1986). These two cell lines provide an in vitro system in which infected and non‐infected cells represent identical bovine genotypes. Cells were routinely maintained in culture at 37°C in RPMI with 20% foetal calf serum (FCS) as described previously (Shiels et al., 1992).

### 4.2 Isolation and purification of EV

Cells for EV isolation were counted, washed three times in supplement‐free RPMI, resuspended at 1 × 10^6^ cells/ml in RPMI plus 20% EV depleted FCS (Stratech), and incubated at 37°C for 12 hr prior to EV preparation. EV were initially isolated from culture supernatants of BL20 control or TBL20 infected cells by ultracentrifugation as previously described (Thery, Amigorena, Raposo, & Clayton, 2006). Briefly, 100 ml of culture was centrifuged at 300× *g* for 10 min, 2,000× *g* for 10 min, and 10,000× *g* for 30 min, with cells and cell debris discarded at each stage. EV were then pelleted by ultracentrifugation using a Surespin 630 rotor (Sorvall) at 100,000× *g* for 70 min, washed in sterile PBS, and finally centrifuged at 100,000× *g* for 70 min before resuspending in 100 μl of PBS. EV were used immediately to maximise integrity. When EV were used in cell uptake assays, they were prepared using exoEasy Maxi Kits (Qiagen) to maintain sterility. Postelution from exoEasy columns, EV were concentrated by reducing the volume of elution buffer to 50 μl using Vivaspin 500 centrifugal concentrators (Sartorius).

### 4.3 Visualisation and quantification of EV

EV purified from BL20 and TBL20 cells were resuspended in 2% paraformaldehyde (PFA) and visualised by transmission electron microscopy (TEM; Thery et al., 2006). EV were also visualised and quantified, and their size distribution measured using nanoparticle tracking analysis (NTA) on a NanoSight LM10 with NTA2.3 software (NanoSight Ltd., Minton Park, United Kingdom). EV were tracked and sized using Brownian motion. EV samples for NTA were diluted 1:250 in filtered PBS, which was also used as a negative control. The detection threshold was similar in all samples. NTA quantification of EV was confirmed using the EXOCET exosome quantitation kit read at OD405 (Systembio). EXOCET is an enzymatic, colorimetric assay that directly measures of acetylcholinesterase activity, known to be enriched, within exosomes (Gupta & Knowlton, 2007; Savina, Vidal, & Colombo, 2002).

### 4.4 Western blotting

EV were isolated from culture supernatants as described above, while cell extracts were prepared following standard protocols (Kinnaird et al., 2013a). All proteins were resolved by denaturing SDS‐PAGE (Laemmli, 1970) except when blotting with anti‐CD63 antibodies, where extracts were analysed under non‐reducing conditions (no β‐mercaptoethanol added to sample buffer and no heating to 95°C). Proteins were visualised by Coomassie blue staining of gels or by western blotting, carried out by transfer onto PVDF membrane (Amersham). Blots were probed using the following primary antibodies; mouse monoclonal antibovine CD63 (catalogue no. MCA2042, dilution, 1/500, BioRad), rabbit polyclonal antihuman Rab‐5B (catalogue no. sc‐598, dilution, 1/500, Santa Cruz Biotechnology) and rabbit antibovine Cyc1 (polyclonal, 1/1000, Aviva Systems Biology). HRP‐conjugated secondary antibodies were routinely used at one in 10,000 dilution and binding detected using the Enhanced Chemiluminscent (ECL) system (GE Healthcare).

### 4.5 EV uptake assay

TBL20‐EV were labelled with PKH67 Fluorescent Cell Linker Kit (Sigma), according to the manufacturer's guidelines. Briefly, EV were incubated with 2 × 10^−6^ M PKH67 in Diluent C, supplied with the kit, for 3 min at room temperature. Neat EV‐free FCS was used as a stop solution and was added in equal volume to the reaction. A negative control sample containing EV depleted supernatant from TBL20 was also labelled at the same time. BL20 cells at 5 × 10^5^ in 1 ml of FCS‐free RPMI were incubated with 50 μl of PKH67 labelled TBL20‐EV or TBL20‐EV depleted supernatant for 2 hr at 37°C. After incubation, cells were washed three times and resuspended in 1 ml FCS‐free RPMI and 40 μl of each suspension was analysed by cytospin, fixed in methanol for 30 min and incubated in 0.04% Evan's blue solution. After washing in PBS, slides were air dried at room temperature and mounted with ProLong Gold antifade with DAPI (molecular probes), viewed by florescence microscopy on an Olympus BX60 and images captured and overlayed using SPOT advance software.

### 4.6 Proteomic sample preparation and LC–MS analysis

Six biological replicates were used for proteomic analysis of BL20‐EV and TBL20‐EV. EV pellets were digested and acidified as described previously (Clarke et al., 2017), and the completeness of digestion was verified by SDS‐PAGE analysis of the digest preacidification and postacidification. LC–MS analysis was conducted on a QExactive HF quadrupole‐Orbitrap mass spectrometer coupled to a Dionex Ultimate 3000 RSLC nano‐liquid chromatograph (Hemel Hempstead, United Kingdom) described in (Clarke et al., 2017). Label‐free protein quantification was performed using Progenesis QI for Proteomics v.2.0 (Waters Ltd., Newcastle‐upon‐Tyne, United Kingdom). Raw data files were imported into Progenesis for peak detection and alignment and grouped into control BL20‐EV and TBL20‐EV experimental groups. Data were searched against the UniProt bovine reviewed database using Mascot v.2.4.1 (Matrix Science, London, United Kingdom). The false discovery rate at the peptide‐level was set to 1%, and peptide identifications were further filtered using a peptide ion score of 15 or greater (score indicating identity or extensive homology). Protein quantification was based on averaging the individual abundances for every unique peptide for each protein and comparing the relative abundance across sample runs and between experimental groups. IPA was applied to proteomic data for functional clustering of proteins into defined networks.

### 4.7 miRNA profiling by RNA‐Seq

RNA was prepared from triplicate samples of BL20‐EV and TBL20‐EV using Trizol (Thermo Fisher) according to manufacturer's guidelines. Library preparations and miRNA sequencing, using the Illumina next‐generation sequencing platform along with downstream statistical testing and cluster analysis, was carried out by LC Sciences (Houston, TX, U.S.A.). Cut‐offs of ANOVA *p* < 0.01 and a read count of >500 were applied to the data. miRNA qRT‐PCR was used to confirm the profile of miRNA in BL20 and TBL20 cells and EV. Three control miRNA, which were found by miRNA‐Seq to be present at similar levels between experimental conditions, were tested for consistency between groups, and *bta‐miR‐30e‐3p* was selected as the normaliser gene for all subsequent experiments. Triplicate biological replicates were used throughout and each individual PCR run in triplicate. All primer sequences are outlined in Table S1.

### 4.8 Identifying miRNA targets and confirming miRNA–mRNA interactions in HEK293 cells

3′ UTRs of the genes encoding the top 15 overexpressed proteins in TBL20‐EV were identified using NCBI GENE and UTR database website (http://utrdb.ba.itb.cnr.it/). As a reduction in *bta‐miR‐181a* and *bta‐miR‐181b* in infected cells may result in an increase in target proteins in EV, the 3′ UTR of these 15 genes were analysed for potential *bta‐miR‐181a* and *bta‐miR‐181b* binding sites using the online prediction tool (https://genie.weizmann.ac.il/pubs/mir07/mir07_prediction.html). The PITA algorithm (Kertesz, Iovino, Unnerstall, Gaul, & Segal, 2007) used a minimum seed size of eight, allowing for a single G:U wobble, and a single mismatch and results were refined by considering only those with PITA ΔΔG energies of <−7. The 3′ UTRs from predicted *bta‐mir‐181a* and *bta‐mir‐181b* target genes encoding BOLA class 1 (bovine MHC class 1), ICAM‐1, and MMP‐9 were amplified from TBL20 genomic DNA, using PfuUltra II Fusion HS DNA Polymerase (Agilent). These genes were selected based upon their potential importance in the pathogenesis of *Theileria* infection and relevance to disease progression. Primer sequences are shown in Table S1 (restriction sites underlined).

All 3′ UTR PCR products were cloned into pCR2.1‐TOPO (Invitrogen), and then subcloned into the *Not*I site downstream of firefly luciferase in pMir‐Target (Origene). A 387 bp section of the *bta‐miR‐181a* locus and a 302 bp region of the *bta‐miR‐181b* were amplified from BL20 genomic DNA, cloned into pCR2.1‐TOPO, and subcloned into vector pEGFP‐N1 (Clontech) using *Kpn*I to generate plasmids in the forward and reverse orientation.

HEK293 cells were maintained as described previously (Winter et al., 2015). For transfections, 1 × 10^4^ cells/ml were seeded into the wells of 96‐well plates in a volume of 100 μl using Dulbecco's Modified Eagle's Medium (Sigma D5671). After 24 hr, when cells were approximately 50% confluent, they were transfected using Lipofectamine LTX (Invitrogen) with 50 ng of *bta‐miR‐181a or bta‐miR‐181b‐*containing plasmid in either the forward or reverse direction, 25 ng of the relevant pMir‐Target‐derived plasmid, and 0.5 ng of phRG‐TK (Renilla luciferase, Promega). In experiments where *bta‐miR‐181a and bta‐miR‐181b* were cotransfected, 40 ng of each plasmid was used. Transfections were performed following the manufacturer's protocol for HEK293 cells. DNA was diluted to a volume of 20 μl using Opti‐MEM I Reduced Serum Medium (Invitrogen) and 0.35 μl Lipofectamine LTX Reagent added. After incubation at room temperature for 30 min, 20 μl of the DNA‐Lipofectamine complex was then added directly to the cells in each well. Cells were grown for 48 hr, then analysed using a Dual Luciferase Assay kit (Promega) following the manufactures protocol with six replicates used per test condition. Results are presented relative to the reverse orientation control miRNA, where this control is designated a value of 1.

### 4.9 *bta‐miR‐181a* and *b* mimic experiments

To obtain transient overexpression of *bta‐miR‐181a* or *b* in TBL20 cells, two exogenous miRNA mimics and the miRIDIAN microRNA mimic transfection control, a Dy547‐labelled microRNA mimic based on the Caenorhabditis elegans miRNA *cel‐miR‐67* (miRIDIAN Mimic Negative Control #1; Dharmacon) were transfected into TBL20 cells at 60 nM using an Amaxa Nucleofector (Lonza). This control was used as it is confirmed to have minimal sequence identity with miRNAs in human, mouse, and rat. It has identical design and modifications to miRIDIAN microRNA Mimics and has no identifiable effects on tested miRNA function (https://dharmacon.horizondiscovery.com/rnai/controls/microrna‐mimic/miridian‐microrna‐mimic‐negative‐control‐1/).

Cells were incubated at 37°C for 48 hr and mRNA isolated using Trizol reagent. Comparative quantification of ICAM‐1, BOLA‐1, and MMP‐9 transcripts in TBL20 cells transfected with *miR‐181a/b* mimic or the negative control mimic was carried out by qRT‐PCR. *gapdh* was used as a normalising gene for all mRNA qRT‐PCR (Durrani et al., 2012), and all experiments were carried out in triplicate. In addition, triplicate cell cultures were counted after transfection with mimics and controls using standard Trypan blue methods and a haemocytometer. Predicted putative mRNA targets of *miR‐181a* were generated and pathway analysis on this list of genes was carried out using IPA. Eight hundred and fifty‐eight high and moderate confidence predictions were included in this analysis. This list of genes was cross referenced with previous TBL20 vs. BL20 microarray data from this lab. A cut‐off of FDR (*q*‐value) <0.05 was applied to this data resulting in a list of 1,596 genes upregulated in TBL20 cells (Kinnaird et al., 2013b).

## Supporting information

Data S1 Supporting Information
