# Supplementary material for: Characterisation of infection associated microRNA and protein cargo in extracellular vesicles of Theileria annulata infected leukocytes
Source: Cell Microbiol. 2018 Nov 9;21(1):e12969. doi: 10.1111/cmi.12969 (PMC6492283; doi:10.1111/cmi.12969)
Supplement: Supplementary file 1 — Data S1 Supporting Information [file CMI-21-na-s001.docx]

**S1 Fig. Peptide Statistics.** PCA plots show proteins statistics for individual BL20-EV and TBL20-EV samples.

Protein Statistics


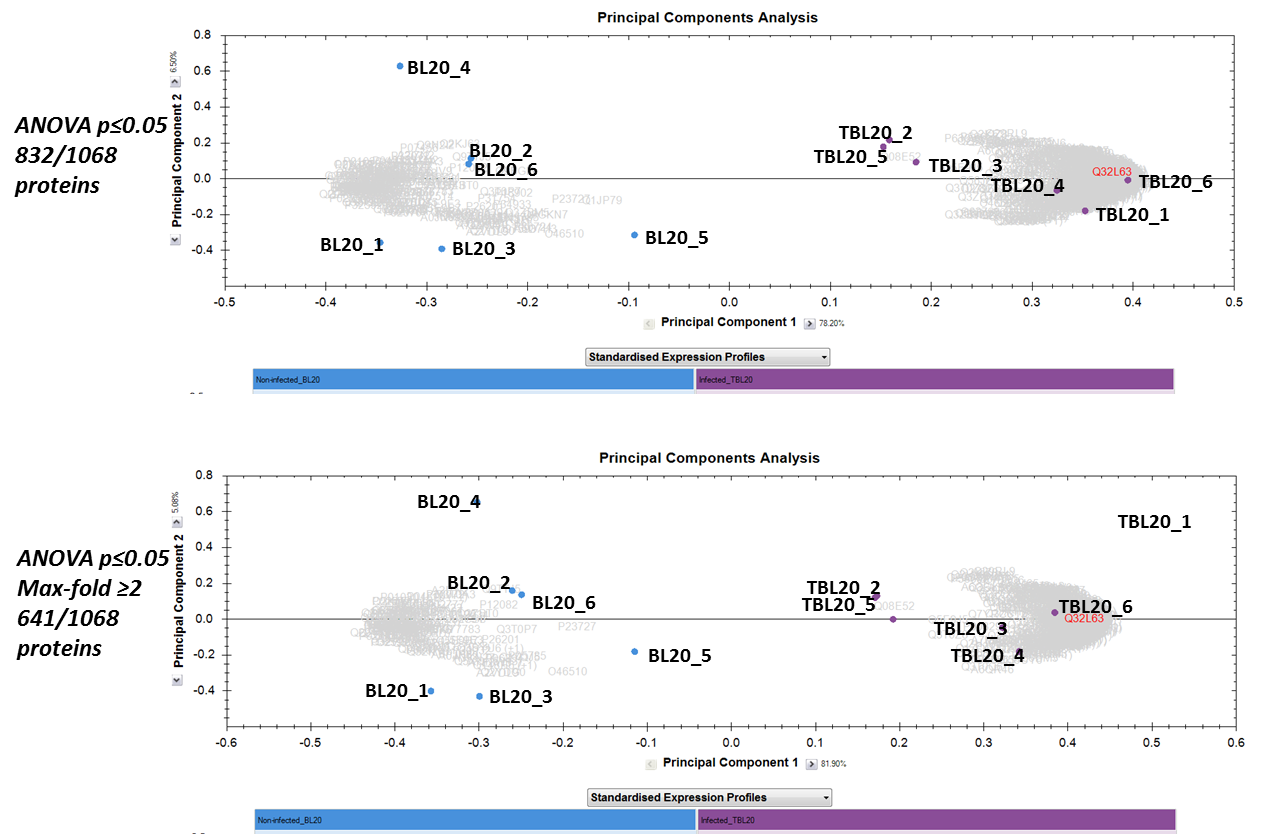


**S2 Fig. BL3-EV vs TBL3-EV and BL3 vs TBL3 cell miRNA qRT-PCR.** Histograms depict expression levels of miRNA of interest in a different *Theileria* infected cell line and in EV secreted by these cells.

**S3 Fig. *icam-1, mmp-9* and *bola-1* expression levels in TBL20 cells.** Histograms depict expression levels of mRNA encoding ICAM-1, MMP-9 and BOLA-1 in TBL20 cells compared to BL20 cells by qRT-PCR.

**S4 Fig Comparative miRNA QRTPCR** – Histograms show increased expression levels of *miR-181a* and *miR-181b* in TBL20 cells transfected with their respective mimics (Dharmacon). No such increase was observed using the negative control mimic *cel-miR-67*. Results are shown relative to expression levels in TBL20 control cells (cells with no mimic).

**S1 Table. Primer sequences.**

| Primer name | Sequence 5’-3’ |
| --- | --- |
| bta-miR-30e-5p | TGTAAACATCCTTGACTGGAAGCT |
| bta-miR-151-5p | TCGAGGAGCTCACAGTCTAGT |
| bta-miR-155 | TTAATGCTAATCGTGATAGGGGT |
| bta-mir-222 | AGCTACATCTGGCTACTGGGT |
| bta-miR-24-3p | TGGCTCAGTTCAGCAGGAACAG |
| bta-miR-27a-3p | TTCACAGTGGCTAAGTTCCG |
| bta-miR-146a | GAGAACTGAATTCCATAGGTTG |
| bta-miR-181a | AACATTCAACGCTGTCGGTGAGTT |
| bta-miR-181b | AACATTCATTGCTGTCGGTGGGTT |
| Universal reverse | GACGAGCTGCCTCAGT |
| MHC I Q F | ATGCGAGTTATGAGGCCG |
| MHC I Q R | CTGCGTGTCGTCCACATAG |
| ICAM1 Q F | CTGCAGCACAGCTTGCGC |
| ICAM1 Q R | CAGGAGTTACTGCAGTTCAC |
| MMP9 Q F | GTCCTGGCGCTCCTGGTG |
| MMP9 Q R | GTGATGTTGTGGTGGTGCCAC |
| GAPDH Q F | GGTGAAGGTCGGAGTGAAC |
| GAPDH Q R | CTGTGCCGTTGAACTTGC |
| mir-181a KpnI F | GGTACCGAGGAATCACGCTTCTTC |
| mir-181a KpnI R | GGTACCGTGAGCTTGTCCACACAG |
| mir-181b KpnI F | GGTACCCAGCCCTACCATCAGTATC |
| mir-181b KpnI R | GGTACCCACCCAGCATAGTAGCAGA |
| ICAM1 UTR NotI F | GCGGCCGCGCCCACCCCAGGATAGGA |
| ICAM1 UTR NotI R | GCGGCCGCAAGGAGTGAGCACCAC |
| MHCI UTR NotI F | GCGGCCGCGACAGCTGCCTTGTG |
| MHCI UTR NotI R | GCGGCCGCACAATAGCCTTAGAAGATAAG |
| MMP9 UTR NotI F | GCGGCCGCGGCTCCCAAGCCTGCT |
| MMP9 UTR NotI R | GCGGCCGCAACAGTGCCCGGCATATA |

**S2 Table. Exosome markers.** The top 100 proteins in the Exocarta database of exosome-associated molecules ([http://www.exocarta.org/exosomemarkers](http://www.exocarta.org/exosome_markers)) and their normalised abundance in TBL20-EV and BL20-EV.

|  | Gene Symbol | Normalized abundance BL20-EV | Normalized abundance TBL20-EV |
| --- | --- | --- | --- |
| 1 | [CD9](http://exocarta.org/query_results?query_name=CD9&prot_name_s=&prot_name_c=) | 5.00E+06 | 2.35E+06 |
| 2 | [HSPA8](http://exocarta.org/query_results?query_name=HSPA8&prot_name_s=&prot_name_c=) | 3.68E+07 | 6.71E+07 |
| 3 | [PDCD6IP](http://exocarta.org/query_results?query_name=PDCD6IP&prot_name_s=&prot_name_c=) | ND | ND |
| 4 | [GAPDH](http://exocarta.org/query_results?query_name=GAPDH&prot_name_s=&prot_name_c=) | 1.77E+07 | 3.73E+07 |
| 5 | [ACTB](http://exocarta.org/query_results?query_name=ACTB&prot_name_s=&prot_name_c=) | 1.71E+08 | 2.29E+08 |
| 6 | [ANXA2](http://exocarta.org/query_results?query_name=ANXA2&prot_name_s=&prot_name_c=) | ND | ND |
| 7 | [CD63](http://exocarta.org/query_results?query_name=CD63&prot_name_s=&prot_name_c=) | 1.74E+06 | 3.37E+06 |
| 8 | [SDCBP](http://exocarta.org/query_results?query_name=SDCBP&prot_name_s=&prot_name_c=) | ND | ND |
| 9 | [ENO1](http://exocarta.org/query_results?query_name=ENO1&prot_name_s=&prot_name_c=) | 5.74E+07 | 9.23E+07 |
| 10 | [HSP90AA1](http://exocarta.org/query_results?query_name=HSP90AA1&prot_name_s=&prot_name_c=) | 9.04E+06 | 1.73E+07 |
| 11 | [TSG101](http://exocarta.org/query_results?query_name=TSG101&prot_name_s=&prot_name_c=) | ND | ND |
| 12 | [PKM](http://exocarta.org/query_results?query_name=PKM&prot_name_s=&prot_name_c=) | ND | ND |
| 13 | [LDHA](http://exocarta.org/query_results?query_name=LDHA&prot_name_s=&prot_name_c=) | 1.25E+07 | 1.87E+07 |
| 14 | [EEF1A1](http://exocarta.org/query_results?query_name=EEF1A1&prot_name_s=&prot_name_c=) | 2.15E+07 | 6.07E+07 |
| 15 | [YWHAZ](http://exocarta.org/query_results?query_name=YWHAZ&prot_name_s=&prot_name_c=) | 7.90E+06 | 1.10E+07 |
| 16 | [PGK1](http://exocarta.org/query_results?query_name=PGK1&prot_name_s=&prot_name_c=) | 1.02E+07 | 2.05E+07 |
| 17 | [EEF2](http://exocarta.org/query_results?query_name=EEF2&prot_name_s=&prot_name_c=) | 1.14E+07 | 2.79E+07 |
| 18 | [ALDOA](http://exocarta.org/query_results?query_name=ALDOA&prot_name_s=&prot_name_c=) | ND | ND |
| 19 | [HSP90AB1](http://exocarta.org/query_results?query_name=HSP90AB1&prot_name_s=&prot_name_c=) | 1.02E+07 | 3.04E+07 |
| 20 | [ANXA5](http://exocarta.org/query_results?query_name=ANXA5&prot_name_s=&prot_name_c=) | ND | ND |
| 21 | [FASN](http://exocarta.org/query_results?query_name=FASN&prot_name_s=&prot_name_c=) | 2.13E+06 | 5.90E+06 |
| 22 | [YWHAE](http://exocarta.org/query_results?query_name=YWHAE&prot_name_s=&prot_name_c=) | 6.17E+06 | 6.81E+06 |
| 23 | [CLTC](http://exocarta.org/query_results?query_name=CLTC&prot_name_s=&prot_name_c=) | 6.42E+06 | 1.74E+07 |
| 24 | [CD81](http://exocarta.org/query_results?query_name=CD81&prot_name_s=&prot_name_c=) | 1.32E+07 | 1.55E+07 |
| 25 | [ALB](http://exocarta.org/query_results?query_name=ALB&prot_name_s=&prot_name_c=) | 4.06E+09 | 1.26E+09 |
| 26 | [VCP](http://exocarta.org/query_results?query_name=VCP&prot_name_s=&prot_name_c=) | 5.70E+06 | 1.16E+07 |
| 27 | [TPI1](http://exocarta.org/query_results?query_name=TPI1&prot_name_s=&prot_name_c=) | 7.03E+06 | 1.48E+07 |
| 28 | [PPIA](http://exocarta.org/query_results?query_name=PPIA&prot_name_s=&prot_name_c=) | 6.68E+06 | 1.20E+07 |
| 29 | [MSN](http://exocarta.org/query_results?query_name=MSN&prot_name_s=&prot_name_c=) | 1.62E+07 | 8.59E+06 |
| 30 | [CFL1](http://exocarta.org/query_results?query_name=CFL1&prot_name_s=&prot_name_c=) | 2.25E+07 | 2.93E+07 |
| 31 | [PRDX1](http://exocarta.org/query_results?query_name=PRDX1&prot_name_s=&prot_name_c=) | 8.02E+06 | 1.57E+07 |
| 32 | [PFN1](http://exocarta.org/query_results?query_name=PFN1&prot_name_s=&prot_name_c=) | 9.10E+06 | 1.65E+07 |
| 33 | [RAP1B](http://exocarta.org/query_results?query_name=RAP1B&prot_name_s=&prot_name_c=) | 5.12E+05 | 1.29E+06 |
| 34 | [ITGB1](http://exocarta.org/query_results?query_name=ITGB1&prot_name_s=&prot_name_c=) | 3.98E+06 | 1.20E+07 |
| 35 | [HSPA5](http://exocarta.org/query_results?query_name=HSPA5&prot_name_s=&prot_name_c=) | 1.30E+06 | 3.91E+06 |
| 36 | [SLC3A2](http://exocarta.org/query_results?query_name=SLC3A2&prot_name_s=&prot_name_c=) | ND | ND |
| 37 | [HIST1H4A](http://exocarta.org/query_results?query_name=HIST1H4A&prot_name_s=&prot_name_c=) | ND | ND |
| 38 | [GNB2](http://exocarta.org/query_results?query_name=GNB2&prot_name_s=&prot_name_c=) | 2.00E+06 | 4.64E+06 |
| 39 | [ATP1A1](http://exocarta.org/query_results?query_name=ATP1A1&prot_name_s=&prot_name_c=) | 7.58E+06 | 1.53E+07 |
| 40 | [YWHAQ](http://exocarta.org/query_results?query_name=YWHAQ&prot_name_s=&prot_name_c=) | 1.59E+06 | 3.47E+06 |
| 41 | [FLOT1](http://exocarta.org/query_results?query_name=FLOT1&prot_name_s=&prot_name_c=) | 7.53E+05 | 1.12E+06 |
| 42 | [FLNA](http://exocarta.org/query_results?query_name=FLNA&prot_name_s=&prot_name_c=) | ND | ND |
| 43 | [CLIC1](http://exocarta.org/query_results?query_name=CLIC1&prot_name_s=&prot_name_c=) | 9.15E+06 | 3.61E+06 |
| 44 | [CDC42](http://exocarta.org/query_results?query_name=CDC42&prot_name_s=&prot_name_c=) | 2.45E+06 | 6.79E+06 |
| 45 | [CCT2](http://exocarta.org/query_results?query_name=CCT2&prot_name_s=&prot_name_c=) | 5.43E+06 | 9.11E+06 |
| 46 | [A2M](http://exocarta.org/query_results?query_name=A2M&prot_name_s=&prot_name_c=) | 2.23E+09 | 8.12E+08 |
| 47 | [YWHAG](http://exocarta.org/query_results?query_name=YWHAG&prot_name_s=&prot_name_c=) | 5.05E+05 | 7.99E+05 |
| 48 | [TUBA1B](http://exocarta.org/query_results?query_name=TUBA1B&prot_name_s=&prot_name_c=) | ND | ND |
| 49 | [RAC1](http://exocarta.org/query_results?query_name=RAC1&prot_name_s=&prot_name_c=) | 2.07E+06 | 3.97E+06 |
| 50 | [LGALS3BP](http://exocarta.org/query_results?query_name=LGALS3BP&prot_name_s=&prot_name_c=) | 2.37E+06 | 1.05E+06 |
| 51 | [HSPA1A](http://exocarta.org/query_results?query_name=HSPA1A&prot_name_s=&prot_name_c=) | ND | ND |
| 52 | [GNAI2](http://exocarta.org/query_results?query_name=GNAI2&prot_name_s=&prot_name_c=) | ND | ND |
| 53 | [ANXA1](http://exocarta.org/query_results?query_name=ANXA1&prot_name_s=&prot_name_c=) | 1.28E+06 | 1.85E+06 |
| 54 | [RHOA](http://exocarta.org/query_results?query_name=RHOA&prot_name_s=&prot_name_c=) | 5.04E+06 | 7.64E+06 |
| 55 | [MFGE8](http://exocarta.org/query_results?query_name=MFGE8&prot_name_s=&prot_name_c=) | 1.44E+06 | 9.54E+06 |
| 56 | [PRDX2](http://exocarta.org/query_results?query_name=PRDX2&prot_name_s=&prot_name_c=) | 9.92E+05 | 6.54E+05 |
| 57 | [GDI2](http://exocarta.org/query_results?query_name=GDI2&prot_name_s=&prot_name_c=) | 3.21E+06 | 6.23E+06 |
| 58 | [EHD4](http://exocarta.org/query_results?query_name=EHD4&prot_name_s=&prot_name_c=) | ND | ND |
| 59 | [ACTN4](http://exocarta.org/query_results?query_name=ACTN4&prot_name_s=&prot_name_c=) | 9.18E+06 | 1.27E+07 |
| 60 | [YWHAB](http://exocarta.org/query_results?query_name=YWHAB&prot_name_s=&prot_name_c=) | 4.05E+06 | 4.97E+06 |
| 61 | [RAB7A](http://exocarta.org/query_results?query_name=RAB7A&prot_name_s=&prot_name_c=) | 2.14E+06 | 5.67E+06 |
| 62 | [LDHB](http://exocarta.org/query_results?query_name=LDHB&prot_name_s=&prot_name_c=) | 6.37E+06 | 1.48E+07 |
| 63 | [GNAS](http://exocarta.org/query_results?query_name=GNAS&prot_name_s=&prot_name_c=) | 1.90E+06 | 4.36E+06 |
| 64 | [TFRC](http://exocarta.org/query_results?query_name=TFRC&prot_name_s=&prot_name_c=) | ND | ND |
| 65 | [RAB5C](http://exocarta.org/query_results?query_name=RAB5C&prot_name_s=&prot_name_c=) | 7.03E+05 | 1.41E+06 |
| 66 | [ARF1](http://exocarta.org/query_results?query_name=ARF1&prot_name_s=&prot_name_c=) | ND | ND |
| 67 | [ANXA6](http://exocarta.org/query_results?query_name=ANXA6&prot_name_s=&prot_name_c=) | 1.90E+07 | 6.83E+07 |
| 68 | [ANXA11](http://exocarta.org/query_results?query_name=ANXA11&prot_name_s=&prot_name_c=) | 3.81E+05 | 9.88E+05 |
| 69 | [ACTG1](http://exocarta.org/query_results?query_name=ACTG1&prot_name_s=&prot_name_c=) | ND | ND |
| 70 | [KPNB1](http://exocarta.org/query_results?query_name=KPNB1&prot_name_s=&prot_name_c=) | ND | ND |
| 71 | [EZR](http://exocarta.org/query_results?query_name=EZR&prot_name_s=&prot_name_c=) | 1.33E+07 | 1.05E+07 |
| 72 | [ANXA4](http://exocarta.org/query_results?query_name=ANXA4&prot_name_s=&prot_name_c=) | 4.35E+05 | 1.12E+06 |
| 73 | [ACLY](http://exocarta.org/query_results?query_name=ACLY&prot_name_s=&prot_name_c=) | 7.51E+06 | 3.19E+06 |
| 74 | [TUBA1C](http://exocarta.org/query_results?query_name=TUBA1C&prot_name_s=&prot_name_c=) | 3.38E+04 | 1.92E+05 |
| 75 | [RAB14](http://exocarta.org/query_results?query_name=RAB14&prot_name_s=&prot_name_c=) | ND | ND |
| 76 | [HIST2H4A](http://exocarta.org/query_results?query_name=HIST2H4A&prot_name_s=&prot_name_c=) | ND | ND |
| 77 | [GNB1](http://exocarta.org/query_results?query_name=GNB1&prot_name_s=&prot_name_c=) | 3.78E+06 | 5.40E+06 |
| 78 | [UBA1](http://exocarta.org/query_results?query_name=UBA1&prot_name_s=&prot_name_c=) | 1.90E+03 | 1.18E+04 |
| 79 | [THBS1](http://exocarta.org/query_results?query_name=THBS1&prot_name_s=&prot_name_c=) | 1.71E+07 | 7.32E+06 |
| 80 | [RAN](http://exocarta.org/query_results?query_name=RAN&prot_name_s=&prot_name_c=) | 3.68E+06 | 5.40E+06 |
| 81 | [RAB5A](http://exocarta.org/query_results?query_name=RAB5A&prot_name_s=&prot_name_c=) | 1.37E+05 | 1.73E+05 |
| 82 | [PTGFRN](http://exocarta.org/query_results?query_name=PTGFRN&prot_name_s=&prot_name_c=) | ND | ND |
| 83 | [CCT5](http://exocarta.org/query_results?query_name=CCT5&prot_name_s=&prot_name_c=) | ND | ND |
| 84 | [CCT3](http://exocarta.org/query_results?query_name=CCT3&prot_name_s=&prot_name_c=) | 2.45E+06 | 5.48E+06 |
| 85 | [BSG](http://exocarta.org/query_results?query_name=BSG&prot_name_s=&prot_name_c=) | ND | ND |
| 86 | [AHCY](http://exocarta.org/query_results?query_name=AHCY&prot_name_s=&prot_name_c=) | 2.12E+06 | 2.58E+06 |
| 87 | [RAB5B](http://exocarta.org/query_results?query_name=RAB5B&prot_name_s=&prot_name_c=) | ND | ND |
| 88 | [RAB1A](http://exocarta.org/query_results?query_name=RAB1A&prot_name_s=&prot_name_c=) | ND | ND |
| 89 | [LAMP2](http://exocarta.org/query_results?query_name=LAMP2&prot_name_s=&prot_name_c=) | ND | ND |
| 90 | [ITGA6](http://exocarta.org/query_results?query_name=ITGA6&prot_name_s=&prot_name_c=) | ND | ND |
| 91 | [HIST1H4B](http://exocarta.org/query_results?query_name=HIST1H4B&prot_name_s=&prot_name_c=) | ND | ND |
| 92 | [GSN](http://exocarta.org/query_results?query_name=GSN&prot_name_s=&prot_name_c=) | 3.85E+07 | 2.03E+07 |
| 93 | [FN1](http://exocarta.org/query_results?query_name=FN1&prot_name_s=&prot_name_c=) | 9.10E+06 | 1.65E+07 |
| 94 | [YWHAH](http://exocarta.org/query_results?query_name=YWHAH&prot_name_s=&prot_name_c=) | 4.34E+05 | 3.71E+05 |
| 95 | [TUBA1A](http://exocarta.org/query_results?query_name=TUBA1A&prot_name_s=&prot_name_c=) | ND | ND |
| 96 | [TKT](http://exocarta.org/query_results?query_name=TKT&prot_name_s=&prot_name_c=) | 7.58E+05 | 3.24E+06 |
| 97 | [TCP1](http://exocarta.org/query_results?query_name=TCP1&prot_name_s=&prot_name_c=) | 3.76E+06 | 8.64E+06 |
| 98 | [STOM](http://exocarta.org/query_results?query_name=STOM&prot_name_s=&prot_name_c=) | ND | ND |
| 99 | [SLC16A1](http://exocarta.org/query_results?query_name=SLC16A1&prot_name_s=&prot_name_c=) | 2.84E+06 | 9.65E+06 |
| 100 | [RAB8A](http://exocarta.org/query_results?query_name=RAB8A&prot_name_s=&prot_name_c=) | 3.31E+05 | 4.62E+05 |

**S3 Table. Protein lists.**  Only those proteins identified by two or more unique peptides and exhibiting a fold change ≥2 and statistical significance (ANOVA p ≤0.05) are listed. 395 elevated in TBL20-EV compared with BL20-EV, 84 elevated in BL20-EV compared to TBL20-EV.

**395 Proteins up-regulated in TBL20 EV**

| **Accession** | **Peptide count** | **Unique peptides** | **Confidence score** | **Anova (p)** | **q Value** | **Max fold change** | **Description** |
| --- | --- | --- | --- | --- | --- | --- | --- |
| Q32L63 | 6 | 6 | 315.65 | 1.69E-10 | 3.13E-08 | 16.48894 | VTA1_BOVIN Vacuolar protein sorting-associated protein VTA1 homolog OS=Bos taurus GN=VTA1 PE=2 SV=1 |
| Q95132 | 14 | 12 | 666.39 | 5.35E-10 | 4.96E-08 | 9.602762 | ICAM1_BOVIN Intercellular adhesion molecule 1 OS=Bos taurus GN=ICAM1 PE=2 SV=1 |
| P06836 | 3 | 3 | 97.5 | 1.14E-09 | 7.06E-08 | 56.21416 | NEUM_BOVIN Neuromodulin OS=Bos taurus GN=GAP43 PE=1 SV=3 |
| Q32KU6 | 4 | 4 | 157.79 | 2.89E-09 | 8.72E-08 | 12.61466 | TSN6_BOVIN Tetraspanin-6 OS=Bos taurus GN=TSPAN6 PE=2 SV=1 |
| Q0VD48 | 9 | 9 | 387.72 | 3.22E-09 | 8.72E-08 | 9.537893 | VPS4B_BOVIN Vacuolar protein sorting-associated protein 4B OS=Bos taurus GN=VPS4B PE=2 SV=1 |
| Q95114 | 16 | 16 | 937.72 | 2.50E-09 | 8.72E-08 | 6.605176 | MFGM_BOVIN Lactadherin OS=Bos taurus GN=MFGE8 PE=1 SV=2 |
| A2VDL4 | 11 | 11 | 527.58 | 7.98E-09 | 1.85E-07 | 7.262592 | SATT_BOVIN Neutral amino acid transporter A OS=Bos taurus GN=SLC1A4 PE=2 SV=1 |
| Q27977 | 9 | 9 | 448.38 | 9.77E-09 | 2.02E-07 | 6.122219 | ITA5_BOVIN Integrin alpha-5 (Fragment) OS=Bos taurus GN=ITGA5 PE=2 SV=1 |
| Q3ZBV1 | 14 | 14 | 809.94 | 1.44E-08 | 2.58E-07 | 10.5243 | IST1_BOVIN IST1 homolog OS=Bos taurus GN=IST1 PE=2 SV=1 |
| Q5E971 | 5 | 5 | 256.6 | 1.53E-08 | 2.58E-07 | 5.383591 | TMEDA_BOVIN Transmembrane emp24 domain-containing protein 10 OS=Bos taurus GN=TMED10 PE=2 SV=1 |
| Q3T178 | 7 | 6 | 278.93 | 1.88E-08 | 2.91E-07 | 6.806968 | VPS28_BOVIN Vacuolar protein sorting-associated protein 28 homolog OS=Bos taurus GN=VPS28 PE=2 SV=1 |
| Q5E994 | 6 | 6 | 193.98 | 2.10E-08 | 3.00E-07 | 9.072056 | CHM1B_BOVIN Charged multivesicular body protein 1b OS=Bos taurus GN=CHMP1B PE=2 SV=1 |
| P52176 | 6 | 6 | 213.7 | 7.60E-08 | 9.12E-07 | 23.31806 | MMP9_BOVIN Matrix metalloproteinase-9 OS=Bos taurus GN=MMP9 PE=2 SV=1 |
| Q0VCY1 | 2 | 2 | 112.34 | 7.86E-08 | 9.12E-07 | 10.39481 | VAPA_BOVIN Vesicle-associated membrane protein-associated protein A OS=Bos taurus GN=VAPA PE=2 SV=1 |
| A6QQZ7 | 10 | 9 | 308.16 | 7.47E-08 | 9.12E-07 | 7.535415 | MPP7_BOVIN MAGUK p55 subfamily member 7 OS=Bos taurus GN=MPP7 PE=2 SV=1 |
| Q28203 | 4 | 4 | 107.94 | 9.92E-08 | 1.08E-06 | 7.938496 | TNR5_BOVIN Tumor necrosis factor receptor superfamily member 5 OS=Bos taurus GN=CD40 PE=2 SV=3 |
| Q3SYX0 | 3 | 3 | 157.31 | 1.28E-07 | 1.32E-06 | 9.594604 | NDRG1_BOVIN Protein NDRG1 OS=Bos taurus GN=NDRG1 PE=2 SV=1 |
| Q9TU03 | 13 | 10 | 738.06 | 1.38E-07 | 1.35E-06 | 4.977273 | GDIR2_BOVIN Rho GDP-dissociation inhibitor 2 OS=Bos taurus GN=ARHGDIB PE=2 SV=3 |
| Q2HJH1 | 9 | 9 | 382.69 | 1.77E-07 | 1.37E-06 | 8.292936 | DNPEP_BOVIN Aspartyl aminopeptidase OS=Bos taurus GN=DNPEP PE=1 SV=1 |
| A5D7L5 | 5 | 5 | 335.9 | 1.62E-07 | 1.37E-06 | 5.001657 | S39AE_BOVIN Zinc transporter ZIP14 OS=Bos taurus GN=SLC39A14 PE=2 SV=1 |
| P37980 | 9 | 9 | 381.1 | 1.65E-07 | 1.37E-06 | 4.986035 | IPYR_BOVIN Inorganic pyrophosphatase OS=Bos taurus GN=PPA1 PE=1 SV=2 |
| P79134 | 43 | 41 | 2724.4 | 1.62E-07 | 1.37E-06 | 3.603674 | ANXA6_BOVIN Annexin A6 OS=Bos taurus GN=ANXA6 PE=1 SV=2 |
| Q9BGI1 | 5 | 5 | 185.02 | 1.77E-07 | 1.37E-06 | 3.253504 | PRDX5_BOVIN Peroxiredoxin-5, mitochondrial OS=Bos taurus GN=PRDX5 PE=2 SV=2 |
| P01888 | 5 | 5 | 245.89 | 2.02E-07 | 1.43E-06 | 3.146125 | B2MG_BOVIN Beta-2-microglobulin OS=Bos taurus GN=B2M PE=1 SV=2 |
| O46522 | 2 | 2 | 35.43 | 2.62E-07 | 1.52E-06 | 11.43329 | CY24B_BOVIN Cytochrome b-245 heavy chain OS=Bos taurus GN=CYBB PE=2 SV=1 |
| P62194 | 14 | 13 | 529.11 | 2.46E-07 | 1.52E-06 | 4.179675 | PRS8_BOVIN 26S protease regulatory subunit 8 OS=Bos taurus GN=PSMC5 PE=2 SV=1 |
| A5D7A0 | 11 | 11 | 679.38 | 2.61E-07 | 1.52E-06 | 4.118515 | EFHD2_BOVIN EF-hand domain-containing protein D2 OS=Bos taurus GN=EFHD2 PE=2 SV=1 |
| A5PK39 | 27 | 26 | 1021.02 | 2.76E-07 | 1.55E-06 | 4.211543 | TPP2_BOVIN Tripeptidyl-peptidase 2 OS=Bos taurus GN=TPP2 PE=2 SV=1 |
| Q2M2T1 | 9 | 8 | 476.71 | 3.29E-07 | 1.70E-06 | 7.599511 | H2B1K_BOVIN Histone H2B type 1-K OS=Bos taurus GN=HIST1H2BK PE=1 SV=3 |
| Q5E9F8 | 7 | 7 | 197.48 | 3.36E-07 | 1.70E-06 | 6.81556 | H33_BOVIN Histone H3.3 OS=Bos taurus GN=H3F3A PE=1 SV=3 |
| Q1JQC2 | 2 | 2 | 62.05 | 3.47E-07 | 1.70E-06 | 6.628764 | IR3IP_BOVIN Immediate early response 3-interacting protein 1 OS=Bos taurus GN=IER3IP1 PE=3 SV=1 |
| P68399 | 2 | 2 | 52.46 | 3.40E-07 | 1.70E-06 | 6.404317 | CSK21_BOVIN Casein kinase II subunit alpha OS=Bos taurus GN=CSNK2A1 PE=1 SV=1 |
| Q9BH13 | 10 | 10 | 530.45 | 3.49E-07 | 1.70E-06 | 3.197058 | CD166_BOVIN CD166 antigen OS=Bos taurus GN=ALCAM PE=2 SV=1 |
| P56701 | 14 | 14 | 537.21 | 3.58E-07 | 1.70E-06 | 3.786525 | PSMD2_BOVIN 26S proteasome non-ATPase regulatory subunit 2 OS=Bos taurus GN=PSMD2 PE=1 SV=2 |
| P05307 | 17 | 17 | 693.48 | 4.04E-07 | 1.83E-06 | 7.352682 | PDIA1_BOVIN Protein disulfide-isomerase OS=Bos taurus GN=P4HB PE=1 SV=1 |
| Q95JC7 | 12 | 12 | 912.31 | 4.15E-07 | 1.84E-06 | 4.337358 | AAAT_BOVIN Neutral amino acid transporter B(0) OS=Bos taurus GN=SLC1A5 PE=2 SV=1 |
| P31404 | 11 | 10 | 415.18 | 4.53E-07 | 1.96E-06 | 4.064803 | VATA_BOVIN V-type proton ATPase catalytic subunit A OS=Bos taurus GN=ATP6V1A PE=2 SV=2 |
| Q0III3 | 2 | 2 | 55.03 | 4.81E-07 | 2.01E-06 | 8.334505 | DC1I2_BOVIN Cytoplasmic dynein 1 intermediate chain 2 OS=Bos taurus GN=DYNC1I2 PE=1 SV=1 |
| P80209 | 10 | 10 | 482.62 | 4.88E-07 | 2.01E-06 | 6.074035 | CATD_BOVIN Cathepsin D OS=Bos taurus GN=CTSD PE=1 SV=2 |
| Q2T9P4 | 6 | 6 | 247.82 | 6.82E-07 | 2.58E-06 | 4.357149 | NASP_BOVIN Nuclear autoantigenic sperm protein OS=Bos taurus GN=NASP PE=2 SV=2 |
| Q6B855 | 15 | 15 | 573.12 | 6.77E-07 | 2.58E-06 | 4.288747 | TKT_BOVIN Transketolase OS=Bos taurus GN=TKT PE=2 SV=1 |
| A2VDY3 | 5 | 5 | 240.89 | 7.15E-07 | 2.64E-06 | 6.041417 | CHM4A_BOVIN Charged multivesicular body protein 4a OS=Bos taurus GN=CHMP4A PE=2 SV=1 |
| P62803 | 6 | 5 | 503.84 | 7.31E-07 | 2.64E-06 | 5.821129 | H4_BOVIN Histone H4 OS=Bos taurus PE=1 SV=2 |
| Q5E964 | 4 | 4 | 155.82 | 7.50E-07 | 2.64E-06 | 5.198921 | PSD13_BOVIN 26S proteasome non-ATPase regulatory subunit 13 OS=Bos taurus GN=PSMD13 PE=2 SV=1 |
| P61286 | 15 | 15 | 686.18 | 7.55E-07 | 2.64E-06 | 3.546489 | PABP1_BOVIN Polyadenylate-binding protein 1 OS=Bos taurus GN=PABPC1 PE=2 SV=1 |
| Q3MHF7 | 10 | 10 | 599.94 | 8.48E-07 | 2.79E-06 | 3.759524 | MTAP_BOVIN S-methyl-5'-thioadenosine phosphorylase OS=Bos taurus GN=MTAP PE=1 SV=1 |
| A3KMV5 | 19 | 19 | 1101.1 | 8.57E-07 | 2.79E-06 | 3.520728 | UBA1_BOVIN Ubiquitin-like modifier-activating enzyme 1 OS=Bos taurus GN=UBA1 PE=2 SV=1 |
| Q5E954 | 10 | 10 | 437.41 | 8.37E-07 | 2.79E-06 | 2.763283 | DNJA1_BOVIN DnaJ homolog subfamily A member 1 OS=Bos taurus GN=DNAJA1 PE=2 SV=2 |
| Q2YDM1 | 3 | 3 | 81.16 | 1.02E-06 | 3.15E-06 | 8.030948 | ARL1_BOVIN ADP-ribosylation factor-like protein 1 OS=Bos taurus GN=ARL1 PE=2 SV=1 |
| Q17QQ1 | 4 | 4 | 148.98 | 1.00E-06 | 3.15E-06 | 4.421005 | LTOR3_BOVIN Ragulator complex protein LAMTOR3 OS=Bos taurus GN=LAMTOR3 PE=2 SV=1 |
| Q3ZBE9 | 2 | 2 | 44.36 | 1.13E-06 | 3.43E-06 | 19.4887 | NSDHL_BOVIN Sterol-4-alpha-carboxylate 3-dehydrogenase, decarboxylating OS=Bos taurus GN=NSDHL PE=2 SV=1 |
| Q3ZBV8 | 23 | 22 | 910.71 | 1.17E-06 | 3.44E-06 | 3.25821 | SYTC_BOVIN Threonine--tRNA ligase, cytoplasmic OS=Bos taurus GN=TARS PE=2 SV=1 |
| Q2HJH9 | 3 | 3 | 121.63 | 1.15E-06 | 3.44E-06 | 2.895377 | PDCD5_BOVIN Programmed cell death protein 5 OS=Bos taurus GN=PDCD5 PE=2 SV=3 |
| Q0VCY0 | 3 | 3 | 119.2 | 1.26E-06 | 3.62E-06 | 48.62241 | AT2A1_BOVIN Sarcoplasmic/endoplasmic reticulum calcium ATPase 1 OS=Bos taurus GN=ATP2A1 PE=1 SV=1 |
| A4FUZ5 | 8 | 8 | 431.28 | 1.27E-06 | 3.62E-06 | 3.648976 | SERC3_BOVIN Serine incorporator 3 OS=Bos taurus GN=SERINC3 PE=2 SV=1 |
| Q9TU25 | 7 | 4 | 381.82 | 1.35E-06 | 3.75E-06 | 4.703449 | RAC2_BOVIN Ras-related C3 botulinum toxin substrate 2 OS=Bos taurus GN=RAC2 PE=2 SV=1 |
| A1A4K3 | 7 | 7 | 170.82 | 1.34E-06 | 3.75E-06 | 4.265338 | DDB1_BOVIN DNA damage-binding protein 1 OS=Bos taurus GN=DDB1 PE=2 SV=1 |
| O77834 | 16 | 16 | 755.48 | 1.40E-06 | 3.82E-06 | 3.658838 | PRDX6_BOVIN Peroxiredoxin-6 OS=Bos taurus GN=PRDX6 PE=1 SV=3 |
| Q3MHL8 | 2 | 2 | 144.65 | 1.52E-06 | 4.08E-06 | 21.91838 | SMAP_BOVIN Small acidic protein OS=Bos taurus GN=SMAP PE=2 SV=1 |
| Q2KHU0 | 5 | 5 | 309.55 | 1.54E-06 | 4.08E-06 | 5.189735 | SERB_BOVIN Phosphoserine phosphatase OS=Bos taurus GN=PSPH PE=2 SV=1 |
| Q05588 | 6 | 6 | 394.21 | 1.60E-06 | 4.19E-06 | 7.395598 | UPAR_BOVIN Urokinase plasminogen activator surface receptor OS=Bos taurus GN=PLAUR PE=2 SV=1 |
| Q3ZBF7 | 8 | 8 | 377.07 | 1.64E-06 | 4.24E-06 | 3.495883 | TEBP_BOVIN Prostaglandin E synthase 3 OS=Bos taurus GN=PTGES3 PE=1 SV=1 |
| Q3T0B2 | 8 | 8 | 315.87 | 1.78E-06 | 4.53E-06 | 3.755379 | PSMD6_BOVIN 26S proteasome non-ATPase regulatory subunit 6 OS=Bos taurus GN=PSMD6 PE=2 SV=1 |
| Q95L46 | 3 | 3 | 64.85 | 1.81E-06 | 4.54E-06 | 12.72795 | IF4G2_BOVIN Eukaryotic translation initiation factor 4 gamma 2 OS=Bos taurus GN=EIF4G2 PE=2 SV=2 |
| Q29423 | 9 | 9 | 611.93 | 1.91E-06 | 4.73E-06 | 4.689529 | CD44_BOVIN CD44 antigen OS=Bos taurus GN=CD44 PE=2 SV=1 |
| A6QPY0 | 3 | 3 | 94.12 | 2.08E-06 | 5.02E-06 | 5.030241 | OST48_BOVIN Dolichyl-diphosphooligosaccharide--protein glycosyltransferase 48 kDa subunit OS=Bos taurus GN=DDOST PE=2 SV=2 |
| Q5E958 | 8 | 8 | 473.05 | 2.21E-06 | 5.26E-06 | 3.528524 | RS8_BOVIN 40S ribosomal protein S8 OS=Bos taurus GN=RPS8 PE=2 SV=3 |
| Q5MD62 | 5 | 5 | 120.06 | 2.25E-06 | 5.29E-06 | 4.796185 | CCR7_BOVIN C-C chemokine receptor type 7 OS=Bos taurus GN=CCR7 PE=2 SV=1 |
| P48452 | 6 | 6 | 211.2 | 2.32E-06 | 5.38E-06 | 3.799191 | PP2BA_BOVIN Serine/threonine-protein phosphatase 2B catalytic subunit alpha isoform OS=Bos taurus GN=PPP3CA PE=1 SV=1 |
| Q3T181 | 2 | 2 | 86.35 | 2.36E-06 | 5.41E-06 | 10.00793 | LT4R1_BOVIN Leukotriene B4 receptor 1 OS=Bos taurus GN=LTB4R PE=2 SV=1 |
| Q5E9X4 | 3 | 3 | 90.99 | 2.39E-06 | 5.41E-06 | 6.5286 | LRC59_BOVIN Leucine-rich repeat-containing protein 59 OS=Bos taurus GN=LRRC59 PE=2 SV=1 |
| Q95M18 | 10 | 9 | 455.56 | 2.66E-06 | 5.94E-06 | 3.425626 | ENPL_BOVIN Endoplasmin OS=Bos taurus GN=HSP90B1 PE=2 SV=1 |
| Q9TRY0 | 10 | 9 | 456.54 | 2.81E-06 | 6.20E-06 | 4.826883 | FKBP4_BOVIN Peptidyl-prolyl cis-trans isomerase FKBP4 OS=Bos taurus GN=FKBP4 PE=1 SV=4 |
| Q9XSJ0 | 3 | 3 | 80.54 | 3.20E-06 | 6.82E-06 | 9.337464 | UCHL5_BOVIN Ubiquitin carboxyl-terminal hydrolase isozyme L5 OS=Bos taurus GN=UCHL5 PE=2 SV=1 |
| Q32PF3 | 2 | 2 | 57.03 | 3.13E-06 | 6.82E-06 | 5.951857 | PCNP_BOVIN PEST proteolytic signal-containing nuclear protein OS=Bos taurus GN=PCNP PE=2 SV=1 |
| Q3ZBW4 | 6 | 6 | 316.26 | 3.17E-06 | 6.82E-06 | 3.598299 | PCNA_BOVIN Proliferating cell nuclear antigen OS=Bos taurus GN=PCNA PE=2 SV=1 |
| Q59A32 | 16 | 16 | 695.15 | 3.38E-06 | 7.04E-06 | 4.371598 | PUR2_BOVIN Trifunctional purine biosynthetic protein adenosine-3 OS=Bos taurus GN=GART PE=2 SV=1 |
| P28801 | 12 | 12 | 899.19 | 3.36E-06 | 7.04E-06 | 2.894622 | GSTP1_BOVIN Glutathione S-transferase P OS=Bos taurus GN=GSTP1 PE=1 SV=2 |
| Q3SZI6 | 4 | 4 | 170.79 | 3.58E-06 | 7.37E-06 | 14.9955 | RPN2_BOVIN Dolichyl-diphosphooligosaccharide--protein glycosyltransferase subunit 2 OS=Bos taurus GN=RPN2 PE=2 SV=1 |
| Q76LV1 | 41 | 24 | 2399.13 | 3.73E-06 | 7.53E-06 | 3.141064 | HS90B_BOVIN Heat shock protein HSP 90-beta OS=Bos taurus GN=HSP90AB1 PE=2 SV=3 |
| P61283 | 2 | 2 | 50.75 | 3.84E-06 | 7.65E-06 | 3.781083 | BAF_BOVIN Barrier-to-autointegration factor OS=Bos taurus GN=BANF1 PE=3 SV=1 |
| P13752 | 5 | 2 | 353.48 | 4.38E-06 | 8.47E-06 | 3.775177 | HA1A_BOVIN BOLA class I histocompatibility antigen, alpha chain BL3-6 OS=Bos taurus PE=2 SV=1 |
| Q3SZC4 | 8 | 8 | 397.34 | 4.48E-06 | 8.57E-06 | 4.19665 | NSF1C_BOVIN NSFL1 cofactor p47 OS=Bos taurus GN=NSFL1C PE=2 SV=1 |
| Q1RMX2 | 2 | 2 | 89.79 | 4.61E-06 | 8.64E-06 | 2.751838 | UB2D2_BOVIN Ubiquitin-conjugating enzyme E2 D2 OS=Bos taurus GN=UBE2D2 PE=2 SV=1 |
| Q8HZJ5 | 2 | 2 | 59.82 | 4.89E-06 | 8.70E-06 | 4.725341 | RB27B_BOVIN Ras-related protein Rab-27B OS=Bos taurus GN=RAB27B PE=2 SV=3 |
| Q3SYW6 | 10 | 10 | 430.57 | 4.76E-06 | 8.70E-06 | 3.849279 | EIF3C_BOVIN Eukaryotic translation initiation factor 3 subunit C OS=Bos taurus GN=EIF3C PE=2 SV=1 |
| Q9TR36 | 4 | 3 | 93.71 | 4.92E-06 | 8.70E-06 | 3.739263 | PIPNB_BOVIN Phosphatidylinositol transfer protein beta isoform OS=Bos taurus GN=PITPNB PE=1 SV=3 |
| Q3T160 | 6 | 6 | 294.38 | 4.92E-06 | 8.70E-06 | 2.975546 | NPM_BOVIN Nucleophosmin OS=Bos taurus GN=NPM1 PE=2 SV=1 |
| Q3ZCJ8 | 8 | 8 | 397.09 | 5.22E-06 | 9.10E-06 | 5.36418 | CATC_BOVIN Dipeptidyl peptidase 1 OS=Bos taurus GN=CTSC PE=2 SV=1 |
| Q3T133 | 2 | 2 | 65.76 | 5.46E-06 | 9.22E-06 | 6.068243 | TMED9_BOVIN Transmembrane emp24 domain-containing protein 9 OS=Bos taurus GN=TMED9 PE=1 SV=1 |
| Q2T9X2 | 24 | 24 | 1203.8 | 5.43E-06 | 9.22E-06 | 3.037867 | TCPD_BOVIN T-complex protein 1 subunit delta OS=Bos taurus GN=CCT4 PE=1 SV=3 |
| Q2KJ61 | 2 | 2 | 58.95 | 5.62E-06 | 9.40E-06 | 6.705321 | ELP3_BOVIN Elongator complex protein 3 OS=Bos taurus GN=ELP3 PE=2 SV=2 |
| O02739 | 4 | 3 | 169.28 | 5.92E-06 | 9.81E-06 | 2.968402 | SPB6_BOVIN Serpin B6 OS=Bos taurus GN=SERPINB6 PE=2 SV=1 |
| Q2KJ25 | 7 | 7 | 236.89 | 6.53E-06 | 1.06E-05 | 3.072038 | PSD12_BOVIN 26S proteasome non-ATPase regulatory subunit 12 OS=Bos taurus GN=PSMD12 PE=2 SV=3 |
| O97680 | 8 | 8 | 405.12 | 6.55E-06 | 1.06E-05 | 2.858117 | THIO_BOVIN Thioredoxin OS=Bos taurus GN=TXN PE=3 SV=3 |
| P26882 | 13 | 12 | 536.68 | 6.87E-06 | 1.09E-05 | 3.789591 | PPID_BOVIN Peptidyl-prolyl cis-trans isomerase D OS=Bos taurus GN=PPID PE=1 SV=6 |
| P35466 | 4 | 4 | 188.12 | 7.07E-06 | 1.11E-05 | 4.182316 | S10A4_BOVIN Protein S100-A4 OS=Bos taurus GN=S100A4 PE=1 SV=2 |
| P53620 | 7 | 7 | 228.11 | 7.36E-06 | 1.14E-05 | 4.204179 | COPG1_BOVIN Coatomer subunit gamma-1 OS=Bos taurus GN=COPG1 PE=1 SV=1 |
| Q5E9F9 | 6 | 6 | 215.15 | 7.61E-06 | 1.17E-05 | 4.470031 | PRS7_BOVIN 26S protease regulatory subunit 7 OS=Bos taurus GN=PSMC2 PE=2 SV=3 |
| Q58DU7 | 3 | 3 | 97.37 | 8.57E-06 | 1.29E-05 | 2.376312 | SH3L1_BOVIN SH3 domain-binding glutamic acid-rich-like protein OS=Bos taurus GN=SH3BGRL PE=3 SV=1 |
| Q0VCX2 | 19 | 17 | 1027.66 | 8.73E-06 | 1.31E-05 | 3.019288 | GRP78_BOVIN 78 kDa glucose-regulated protein OS=Bos taurus GN=HSPA5 PE=2 SV=1 |
| O18738 | 3 | 2 | 130.98 | 9.33E-06 | 1.36E-05 | 5.553509 | DAG1_BOVIN Dystroglycan OS=Bos taurus GN=DAG1 PE=1 SV=1 |
| A5D785 | 11 | 9 | 544.72 | 9.35E-06 | 1.36E-05 | 3.457611 | XPO2_BOVIN Exportin-2 OS=Bos taurus GN=CSE1L PE=2 SV=1 |
| Q5EAD2 | 15 | 15 | 1246.29 | 9.29E-06 | 1.36E-05 | 2.93933 | SERA_BOVIN D-3-phosphoglycerate dehydrogenase OS=Bos taurus GN=PHGDH PE=2 SV=3 |
| Q6VE48 | 4 | 4 | 365.31 | 9.45E-06 | 1.36E-05 | 3.235423 | MCP_BOVIN Membrane cofactor protein OS=Bos taurus GN=CD46 PE=1 SV=2 |
| Q3T0X5 | 5 | 5 | 166.35 | 9.57E-06 | 1.37E-05 | 2.83685 | PSA1_BOVIN Proteasome subunit alpha type-1 OS=Bos taurus GN=PSMA1 PE=1 SV=1 |
| P13753 | 9 | 6 | 862.27 | 9.66E-06 | 1.37E-05 | 3.812354 | HA1B_BOVIN BOLA class I histocompatibility antigen, alpha chain BL3-7 OS=Bos taurus PE=2 SV=1 |
| P05126 | 11 | 11 | 385.79 | 9.83E-06 | 1.38E-05 | 2.772748 | KPCB_BOVIN Protein kinase C beta type OS=Bos taurus GN=PRKCB PE=2 SV=4 |
| Q71SP7 | 38 | 37 | 1702.18 | 9.91E-06 | 1.38E-05 | 2.779127 | FAS_BOVIN Fatty acid synthase OS=Bos taurus GN=FASN PE=2 SV=1 |
| P36225 | 3 | 3 | 90.52 | 1.04E-05 | 1.42E-05 | 15.46144 | MAP4_BOVIN Microtubule-associated protein 4 OS=Bos taurus GN=MAP4 PE=1 SV=1 |
| Q3ZCJ7 | 20 | 3 | 1259.78 | 1.05E-05 | 1.42E-05 | 5.696058 | TBA1C_BOVIN Tubulin alpha-1C chain OS=Bos taurus GN=TUBA1C PE=1 SV=1 |
| Q3MHW6 | 10 | 10 | 711.32 | 1.06E-05 | 1.42E-05 | 3.412565 | MOT1_BOVIN Monocarboxylate transporter 1 OS=Bos taurus GN=SLC16A1 PE=2 SV=2 |
| Q5E938 | 3 | 3 | 164.17 | 1.07E-05 | 1.43E-05 | 2.834646 | EIF1_BOVIN Eukaryotic translation initiation factor 1 OS=Bos taurus GN=EIF1 PE=3 SV=1 |
| P49951 | 55 | 55 | 2795.18 | 1.10E-05 | 1.45E-05 | 2.711861 | CLH1_BOVIN Clathrin heavy chain 1 OS=Bos taurus GN=CLTC PE=1 SV=1 |
| Q3T0N1 | 3 | 3 | 208.67 | 1.15E-05 | 1.50E-05 | 5.366285 | MB12A_BOVIN Multivesicular body subunit 12A OS=Bos taurus GN=MVB12A PE=2 SV=1 |
| Q58D31 | 3 | 3 | 90.8 | 1.22E-05 | 1.58E-05 | 3.294804 | DHSO_BOVIN Sorbitol dehydrogenase OS=Bos taurus GN=SORD PE=2 SV=3 |
| Q58DW0 | 14 | 14 | 554.99 | 1.28E-05 | 1.64E-05 | 3.407773 | RL4_BOVIN 60S ribosomal protein L4 OS=Bos taurus GN=RPL4 PE=2 SV=3 |
| Q5E995 | 9 | 9 | 526.73 | 1.36E-05 | 1.73E-05 | 2.665995 | RS6_BOVIN 40S ribosomal protein S6 OS=Bos taurus GN=RPS6 PE=2 SV=1 |
| Q3SWX8 | 7 | 7 | 234.24 | 1.37E-05 | 1.73E-05 | 2.401594 | RBBP7_BOVIN Histone-binding protein RBBP7 OS=Bos taurus GN=RBBP7 PE=2 SV=1 |
| Q3ZBG0 | 9 | 9 | 307.51 | 1.38E-05 | 1.74E-05 | 2.594633 | PSA7_BOVIN Proteasome subunit alpha type-7 OS=Bos taurus GN=PSMA7 PE=1 SV=1 |
| Q9XT97 | 4 | 4 | 174.39 | 1.42E-05 | 1.76E-05 | 6.010028 | PSN1_BOVIN Presenilin-1 OS=Bos taurus GN=PSEN1 PE=2 SV=1 |
| Q3ZC13 | 6 | 6 | 186.26 | 1.45E-05 | 1.79E-05 | 3.474067 | AP2M1_BOVIN AP-2 complex subunit mu OS=Bos taurus GN=AP2M1 PE=2 SV=1 |
| Q3T0R4 | 2 | 2 | 37.35 | 1.48E-05 | 1.81E-05 | 4.232924 | DRS7B_BOVIN Dehydrogenase/reductase SDR family member 7B OS=Bos taurus GN=DHRS7B PE=2 SV=1 |
| Q2NL22 | 5 | 4 | 164.43 | 1.51E-05 | 1.83E-05 | 4.648002 | IF4A3_BOVIN Eukaryotic initiation factor 4A-III OS=Bos taurus GN=EIF4A3 PE=2 SV=3 |
| P51867 | 2 | 2 | 58.1 | 1.56E-05 | 1.86E-05 | 26.78161 | TNR6_BOVIN Tumor necrosis factor receptor superfamily member 6 OS=Bos taurus GN=FAS PE=2 SV=1 |
| Q0P5F3 | 4 | 3 | 166.8 | 1.57E-05 | 1.86E-05 | 6.195632 | LIN7C_BOVIN Protein lin-7 homolog C OS=Bos taurus GN=LIN7C PE=2 SV=1 |
| Q2KI42 | 5 | 5 | 193.59 | 1.56E-05 | 1.86E-05 | 4.753771 | PSD11_BOVIN 26S proteasome non-ATPase regulatory subunit 11 OS=Bos taurus GN=PSMD11 PE=2 SV=3 |
| Q2NL24 | 3 | 3 | 57.67 | 1.59E-05 | 1.86E-05 | 5.163306 | PSMG2_BOVIN Proteasome assembly chaperone 2 OS=Bos taurus GN=PSMG2 PE=2 SV=1 |
| P68103 | 17 | 17 | 1188.18 | 1.60E-05 | 1.86E-05 | 2.835257 | EF1A1_BOVIN Elongation factor 1-alpha 1 OS=Bos taurus GN=EEF1A1 PE=1 SV=1 |
| Q1LZA3 | 11 | 10 | 386.2 | 1.60E-05 | 1.86E-05 | 2.494512 | ASNS_BOVIN Asparagine synthetase [glutamine-hydrolyzing] OS=Bos taurus GN=ASNS PE=2 SV=3 |
| Q3T0F5 | 9 | 9 | 497.44 | 1.64E-05 | 1.87E-05 | 2.659589 | RAB7A_BOVIN Ras-related protein Rab-7a OS=Bos taurus GN=RAB7A PE=2 SV=1 |
| Q3SZV3 | 15 | 14 | 567.21 | 1.64E-05 | 1.87E-05 | 2.507608 | EF1G_BOVIN Elongation factor 1-gamma OS=Bos taurus GN=EEF1G PE=2 SV=1 |
| Q3T145 | 14 | 13 | 719.78 | 1.64E-05 | 1.87E-05 | 2.112919 | MDHC_BOVIN Malate dehydrogenase, cytoplasmic OS=Bos taurus GN=MDH1 PE=2 SV=3 |
| Q2KI84 | 6 | 6 | 225.24 | 1.69E-05 | 1.91E-05 | 3.150025 | SYHC_BOVIN Histidine--tRNA ligase, cytoplasmic OS=Bos taurus GN=HARS PE=2 SV=1 |
| P61282 | 2 | 2 | 55.01 | 1.84E-05 | 2.07E-05 | 3.050631 | NEDD8_BOVIN NEDD8 OS=Bos taurus GN=NEDD8 PE=3 SV=1 |
| Q862I1 | 4 | 3 | 218.64 | 1.86E-05 | 2.08E-05 | 3.174407 | RL24_BOVIN 60S ribosomal protein L24 OS=Bos taurus GN=RPL24 PE=2 SV=2 |
| A7YW98 | 15 | 15 | 614.59 | 1.87E-05 | 2.08E-05 | 2.816531 | SYRC_BOVIN Arginine--tRNA ligase, cytoplasmic OS=Bos taurus GN=RARS PE=2 SV=1 |
| P20072 | 8 | 6 | 352.77 | 1.91E-05 | 2.11E-05 | 2.382766 | ANXA7_BOVIN Annexin A7 OS=Bos taurus GN=ANXA7 PE=1 SV=2 |
| Q2KHW7 | 2 | 2 | 52 | 2.00E-05 | 2.17E-05 | 5.247405 | RGS10_BOVIN Regulator of G-protein signaling 10 OS=Bos taurus GN=RGS10 PE=2 SV=1 |
| Q29RL2 | 2 | 2 | 80.64 | 1.99E-05 | 2.17E-05 | 5.127471 | CHRD1_BOVIN Cysteine and histidine-rich domain-containing protein 1 OS=Bos taurus GN=CHORDC1 PE=2 SV=1 |
| Q3ZCK1 | 12 | 12 | 566.37 | 2.08E-05 | 2.25E-05 | 2.429378 | EIF3L_BOVIN Eukaryotic translation initiation factor 3 subunit L OS=Bos taurus GN=EIF3L PE=2 SV=1 |
| P05131 | 4 | 3 | 182.01 | 2.15E-05 | 2.31E-05 | 4.075241 | KAPCB_BOVIN cAMP-dependent protein kinase catalytic subunit beta OS=Bos taurus GN=PRKACB PE=1 SV=2 |
| Q2KJ93 | 8 | 7 | 395.61 | 2.18E-05 | 2.32E-05 | 2.77934 | CDC42_BOVIN Cell division control protein 42 homolog OS=Bos taurus GN=CDC42 PE=1 SV=1 |
| P33097 | 5 | 5 | 176.21 | 2.24E-05 | 2.36E-05 | 3.832267 | AATC_BOVIN Aspartate aminotransferase, cytoplasmic OS=Bos taurus GN=GOT1 PE=1 SV=3 |
| Q2TA08 | 2 | 2 | 54.91 | 2.37E-05 | 2.46E-05 | 3.84667 | BAG5_BOVIN BAG family molecular chaperone regulator 5 OS=Bos taurus GN=BAG5 PE=2 SV=1 |
| Q56JV1 | 3 | 3 | 134.76 | 2.38E-05 | 2.46E-05 | 2.347169 | RS26_BOVIN 40S ribosomal protein S26 OS=Bos taurus GN=RPS26 PE=3 SV=3 |
| Q28141 | 7 | 7 | 233.38 | 2.43E-05 | 2.49E-05 | 3.783425 | DHX9_BOVIN ATP-dependent RNA helicase A OS=Bos taurus GN=DHX9 PE=2 SV=1 |
| P79251 | 2 | 2 | 93.32 | 2.47E-05 | 2.52E-05 | 4.829308 | VATG1_BOVIN V-type proton ATPase subunit G 1 OS=Bos taurus GN=ATP6V1G1 PE=1 SV=3 |
| Q863B3 | 8 | 7 | 247.62 | 2.52E-05 | 2.52E-05 | 3.712549 | SND1_BOVIN Staphylococcal nuclease domain-containing protein 1 OS=Bos taurus GN=SND1 PE=1 SV=1 |
| A5D989 | 11 | 9 | 600.94 | 2.52E-05 | 2.52E-05 | 2.980333 | EF1D_BOVIN Elongation factor 1-delta OS=Bos taurus GN=EEF1D PE=2 SV=2 |
| Q3T147 | 6 | 6 | 219.14 | 2.50E-05 | 2.52E-05 | 2.815237 | DX39B_BOVIN Spliceosome RNA helicase DDX39B OS=Bos taurus GN=DDX39B PE=2 SV=1 |
| Q3T171 | 3 | 3 | 120.25 | 2.55E-05 | 2.53E-05 | 3.438134 | RL36_BOVIN 60S ribosomal protein L36 OS=Bos taurus GN=RPL36 PE=3 SV=3 |
| Q5E9K0 | 8 | 8 | 252.1 | 2.56E-05 | 2.53E-05 | 2.617973 | PSB2_BOVIN Proteasome subunit beta type-2 OS=Bos taurus GN=PSMB2 PE=1 SV=1 |
| Q9GL77 | 7 | 6 | 247.93 | 2.58E-05 | 2.54E-05 | 4.390249 | S4A4_BOVIN Electrogenic sodium bicarbonate cotransporter 1 OS=Bos taurus GN=SLC4A4 PE=1 SV=1 |
| Q08DS7 | 17 | 17 | 664.87 | 2.61E-05 | 2.55E-05 | 2.483719 | AP1B1_BOVIN AP-1 complex subunit beta-1 OS=Bos taurus GN=AP2B1 PE=2 SV=1 |
| A5D7E2 | 3 | 3 | 100.21 | 2.67E-05 | 2.58E-05 | 4.065994 | TM9S4_BOVIN Transmembrane 9 superfamily member 4 OS=Bos taurus GN=TM9SF4 PE=2 SV=2 |
| Q3T0D7 | 3 | 3 | 114.74 | 2.75E-05 | 2.63E-05 | 3.154095 | SAR1A_BOVIN GTP-binding protein SAR1a OS=Bos taurus GN=SAR1A PE=2 SV=1 |
| Q3SYU2 | 40 | 39 | 1995.82 | 2.75E-05 | 2.63E-05 | 2.458279 | EF2_BOVIN Elongation factor 2 OS=Bos taurus GN=EEF2 PE=2 SV=3 |
| Q5E9T9 | 5 | 5 | 146.37 | 2.85E-05 | 2.71E-05 | 4.554165 | RTCB_BOVIN tRNA-splicing ligase RtcB homolog OS=Bos taurus GN=RTCB PE=2 SV=1 |
| Q32LC7 | 5 | 5 | 243.82 | 3.01E-05 | 2.85E-05 | 4.190349 | G3BP1_BOVIN Ras GTPase-activating protein-binding protein 1 OS=Bos taurus GN=G3BP PE=2 SV=1 |
| Q3SYR5 | 4 | 4 | 101.03 | 3.11E-05 | 2.93E-05 | 2.576727 | APOC4_BOVIN Apolipoprotein C-IV OS=Bos taurus GN=APOC4 PE=2 SV=1 |
| Q32LE5 | 4 | 4 | 108.46 | 3.16E-05 | 2.96E-05 | 5.051434 | ASGL1_BOVIN Isoaspartyl peptidase/L-asparaginase OS=Bos taurus GN=ASRGL1 PE=2 SV=1 |
| Q3ZBZ8 | 19 | 19 | 724.04 | 3.20E-05 | 2.98E-05 | 2.493777 | STIP1_BOVIN Stress-induced-phosphoprotein 1 OS=Bos taurus GN=STIP1 PE=2 SV=1 |
| Q3SZ62 | 13 | 13 | 726.01 | 3.26E-05 | 3.01E-05 | 2.646669 | PGAM1_BOVIN Phosphoglycerate mutase 1 OS=Bos taurus GN=PGAM1 PE=2 SV=3 |
| A5PJN2 | 2 | 2 | 80.63 | 3.33E-05 | 3.03E-05 | 6.142644 | ERO1A_BOVIN ERO1-like protein alpha OS=Bos taurus GN=ERO1L PE=2 SV=1 |
| P13214 | 9 | 9 | 401.79 | 3.57E-05 | 3.22E-05 | 2.589237 | ANXA4_BOVIN Annexin A4 OS=Bos taurus GN=ANXA4 PE=1 SV=2 |
| Q3T140 | 2 | 2 | 68.55 | 3.63E-05 | 3.25E-05 | 3.686837 | DLRB1_BOVIN Dynein light chain roadblock-type 1 OS=Bos taurus GN=DYNLRB1 PE=3 SV=1 |
| P09867 | 11 | 9 | 639.97 | 3.83E-05 | 3.41E-05 | 2.527631 | ROA1_BOVIN Heterogeneous nuclear ribonucleoprotein A1 OS=Bos taurus GN=HNRNPA1 PE=1 SV=2 |
| Q8WN55 | 11 | 11 | 583.68 | 3.93E-05 | 3.47E-05 | 3.366703 | PTBP1_BOVIN Polypyrimidine tract-binding protein 1 OS=Bos taurus GN=PTBP1 PE=2 SV=1 |
| A7MBJ5 | 15 | 15 | 571.21 | 4.07E-05 | 3.54E-05 | 3.335246 | CAND1_BOVIN Cullin-associated NEDD8-dissociated protein 1 OS=Bos taurus GN=CAND1 PE=2 SV=1 |
| Q3SZ54 | 16 | 15 | 919.21 | 4.05E-05 | 3.54E-05 | 2.597303 | IF4A1_BOVIN Eukaryotic initiation factor 4A-I OS=Bos taurus GN=EIF4A1 PE=2 SV=1 |
| A7MB16 | 6 | 6 | 282.85 | 4.21E-05 | 3.64E-05 | 4.562853 | EIF3B_BOVIN Eukaryotic translation initiation factor 3 subunit B OS=Bos taurus GN=EIF3B PE=2 SV=1 |
| P23356 | 10 | 10 | 433.18 | 4.20E-05 | 3.64E-05 | 2.912128 | UCHL1_BOVIN Ubiquitin carboxyl-terminal hydrolase isozyme L1 OS=Bos taurus GN=UCHL1 PE=1 SV=2 |
| P33672 | 4 | 4 | 141.48 | 4.30E-05 | 3.69E-05 | 2.585682 | PSB3_BOVIN Proteasome subunit beta type-3 OS=Bos taurus GN=PSMB3 PE=1 SV=3 |
| Q2KHU8 | 8 | 8 | 312.65 | 4.33E-05 | 3.71E-05 | 3.618156 | IF2G_BOVIN Eukaryotic translation initiation factor 2 subunit 3 OS=Bos taurus GN=EIF2S3 PE=2 SV=1 |
| Q3T0Y5 | 3 | 3 | 91.38 | 4.40E-05 | 3.75E-05 | 3.545026 | PSA2_BOVIN Proteasome subunit alpha type-2 OS=Bos taurus GN=PSMA2 PE=1 SV=3 |
| P10895 | 3 | 3 | 62.15 | 4.48E-05 | 3.79E-05 | 18.17663 | PLCD1_BOVIN 1-phosphatidylinositol 4,5-bisphosphate phosphodiesterase delta-1 OS=Bos taurus GN=PLCD1 PE=2 SV=2 |
| Q2KJG3 | 8 | 8 | 319.83 | 4.65E-05 | 3.92E-05 | 3.655096 | SYNC_BOVIN Asparagine--tRNA ligase, cytoplasmic OS=Bos taurus GN=NARS PE=2 SV=3 |
| Q9GMB8 | 9 | 9 | 349.25 | 4.75E-05 | 3.99E-05 | 2.762499 | SYSC_BOVIN Serine--tRNA ligase, cytoplasmic OS=Bos taurus GN=SARS PE=2 SV=3 |
| Q3T0I2 | 2 | 2 | 78.28 | 4.99E-05 | 4.11E-05 | 3.081334 | CATH_BOVIN Pro-cathepsin H OS=Bos taurus GN=CTSH PE=2 SV=1 |
| Q56JZ1 | 5 | 5 | 242.47 | 4.99E-05 | 4.11E-05 | 2.971327 | RL13_BOVIN 60S ribosomal protein L13 OS=Bos taurus GN=RPL13 PE=2 SV=3 |
| Q3ZCI9 | 25 | 25 | 1495.32 | 4.97E-05 | 4.11E-05 | 2.580072 | TCPQ_BOVIN T-complex protein 1 subunit theta OS=Bos taurus GN=CCT8 PE=1 SV=3 |
| Q2TBL6 | 5 | 5 | 233.41 | 5.13E-05 | 4.21E-05 | 2.660757 | TALDO_BOVIN Transaldolase OS=Bos taurus GN=TALDO1 PE=2 SV=1 |
| Q24JY1 | 4 | 4 | 158.59 | 5.27E-05 | 4.31E-05 | 2.737638 | RL23A_BOVIN 60S ribosomal protein L23a OS=Bos taurus GN=RPL23A PE=2 SV=1 |
| Q0P5A6 | 3 | 3 | 95.56 | 5.32E-05 | 4.33E-05 | 4.571047 | PSMD5_BOVIN 26S proteasome non-ATPase regulatory subunit 5 OS=Bos taurus GN=PSMD5 PE=2 SV=1 |
| A7YW45 | 7 | 6 | 259.25 | 5.41E-05 | 4.36E-05 | 3.079126 | ANM5_BOVIN Protein arginine N-methyltransferase 5 OS=Bos taurus GN=PRMT5 PE=2 SV=1 |
| Q3T168 | 9 | 8 | 390.49 | 5.41E-05 | 4.36E-05 | 2.755249 | CYBP_BOVIN Calcyclin-binding protein OS=Bos taurus GN=CACYBP PE=2 SV=1 |
| Q2TBX6 | 7 | 7 | 275.66 | 5.43E-05 | 4.36E-05 | 2.336767 | PSB1_BOVIN Proteasome subunit beta type-1 OS=Bos taurus GN=PSMB1 PE=1 SV=1 |
| Q2KIW6 | 9 | 9 | 326.67 | 5.46E-05 | 4.36E-05 | 2.822158 | PRS10_BOVIN 26S protease regulatory subunit 10B OS=Bos taurus GN=PSMC6 PE=2 SV=1 |
| Q2KJ46 | 7 | 7 | 295.18 | 5.78E-05 | 4.60E-05 | 2.737758 | PSMD3_BOVIN 26S proteasome non-ATPase regulatory subunit 3 OS=Bos taurus GN=PSMD3 PE=2 SV=1 |
| Q5E9I7 | 4 | 4 | 184.11 | 5.92E-05 | 4.67E-05 | 4.230895 | MEP50_BOVIN Methylosome protein 50 OS=Bos taurus GN=WDR77 PE=2 SV=1 |
| Q56K03 | 3 | 3 | 172.14 | 6.04E-05 | 4.73E-05 | 2.95082 | RL27A_BOVIN 60S ribosomal protein L27a OS=Bos taurus GN=RPL27A PE=2 SV=3 |
| Q0VCU8 | 3 | 3 | 105.85 | 6.16E-05 | 4.78E-05 | 2.731838 | EIF3J_BOVIN Eukaryotic translation initiation factor 3 subunit J OS=Bos taurus GN=EIF3J PE=2 SV=1 |
| P67808 | 2 | 2 | 83.77 | 6.53E-05 | 5.01E-05 | 3.056033 | YBOX1_BOVIN Nuclease-sensitive element-binding protein 1 OS=Bos taurus GN=YBX1 PE=2 SV=3 |
| Q28104 | 2 | 2 | 76.76 | 6.65E-05 | 5.08E-05 | 2.785481 | COPE_BOVIN Coatomer subunit epsilon OS=Bos taurus GN=COPE PE=1 SV=3 |
| Q0VCK0 | 23 | 23 | 1108.53 | 6.71E-05 | 5.08E-05 | 2.598846 | PUR9_BOVIN Bifunctional purine biosynthesis protein PURH OS=Bos taurus GN=ATIC PE=2 SV=1 |
| Q5E956 | 17 | 17 | 1032.14 | 6.69E-05 | 5.08E-05 | 2.102184 | TPIS_BOVIN Triosephosphate isomerase OS=Bos taurus GN=TPI1 PE=2 SV=3 |
| Q3T003 | 4 | 4 | 174.32 | 6.79E-05 | 5.12E-05 | 2.577969 | RL18A_BOVIN 60S ribosomal protein L18a OS=Bos taurus GN=RPL18A PE=2 SV=1 |
| Q3ZC84 | 5 | 5 | 168.46 | 6.86E-05 | 5.15E-05 | 2.981283 | CNDP2_BOVIN Cytosolic non-specific dipeptidase OS=Bos taurus GN=CNDP2 PE=2 SV=1 |
| P01250 | 2 | 2 | 101.53 | 7.21E-05 | 5.33E-05 | 6.281915 | THP2_BOVIN Thymopoietin-2 OS=Bos taurus PE=1 SV=1 |
| P53712 | 22 | 22 | 1156.35 | 7.53E-05 | 5.48E-05 | 3.007864 | ITB1_BOVIN Integrin beta-1 OS=Bos taurus GN=ITGB1 PE=1 SV=3 |
| P52175 | 7 | 5 | 373.45 | 7.56E-05 | 5.48E-05 | 2.469235 | NDKA2_BOVIN Nucleoside diphosphate kinase A 2 OS=Bos taurus GN=NME1-2 PE=1 SV=3 |
| P08166 | 6 | 6 | 217.58 | 7.68E-05 | 5.54E-05 | 3.369225 | KAD2_BOVIN Adenylate kinase 2, mitochondrial OS=Bos taurus GN=AK2 PE=1 SV=2 |
| P31081 | 3 | 3 | 115.95 | 7.86E-05 | 5.63E-05 | 8.626482 | CH60_BOVIN 60 kDa heat shock protein, mitochondrial OS=Bos taurus GN=HSPD1 PE=1 SV=2 |
| A6H769 | 5 | 5 | 190.49 | 7.85E-05 | 5.63E-05 | 2.757452 | RS7_BOVIN 40S ribosomal protein S7 OS=Bos taurus GN=RPS7 PE=2 SV=1 |
| Q865V6 | 8 | 8 | 400.84 | 7.95E-05 | 5.63E-05 | 2.686886 | CAPG_BOVIN Macrophage-capping protein OS=Bos taurus GN=CAPG PE=2 SV=1 |
| Q32L40 | 25 | 25 | 1363.02 | 7.97E-05 | 5.63E-05 | 2.300515 | TCPA_BOVIN T-complex protein 1 subunit alpha OS=Bos taurus GN=TCP1 PE=1 SV=1 |
| Q2TBU9 | 9 | 9 | 360.15 | 8.08E-05 | 5.68E-05 | 2.866367 | RUVB2_BOVIN RuvB-like 2 OS=Bos taurus GN=RUVBL2 PE=2 SV=3 |
| Q5E9J1 | 7 | 6 | 374.93 | 8.25E-05 | 5.75E-05 | 2.214614 | HNRPF_BOVIN Heterogeneous nuclear ribonucleoprotein F OS=Bos taurus GN=HNRNPF PE=2 SV=3 |
| Q2KIK0 | 4 | 4 | 111.19 | 8.49E-05 | 5.87E-05 | 2.975996 | SUGT1_BOVIN Suppressor of G2 allele of SKP1 homolog OS=Bos taurus GN=SUGT1 PE=2 SV=1 |
| Q3T112 | 6 | 6 | 158.54 | 8.66E-05 | 5.93E-05 | 3.582263 | PSB8_BOVIN Proteasome subunit beta type-8 OS=Bos taurus GN=PSMB8 PE=1 SV=2 |
| Q3ZCF0 | 5 | 5 | 160.97 | 8.72E-05 | 5.95E-05 | 4.135528 | DCTN2_BOVIN Dynactin subunit 2 OS=Bos taurus GN=DCTN2 PE=2 SV=1 |
| Q3T030 | 6 | 5 | 184.1 | 8.77E-05 | 5.96E-05 | 3.31554 | PRS6B_BOVIN 26S protease regulatory subunit 6B OS=Bos taurus GN=PSMC4 PE=2 SV=1 |
| Q3SZ19 | 2 | 2 | 77.43 | 8.93E-05 | 6.03E-05 | 3.895573 | PSMD9_BOVIN 26S proteasome non-ATPase regulatory subunit 9 OS=Bos taurus GN=PSMD9 PE=1 SV=1 |
| P62833 | 10 | 4 | 644.48 | 9.09E-05 | 6.11E-05 | 3.147393 | RAP1A_BOVIN Ras-related protein Rap-1A OS=Bos taurus GN=RAP1A PE=1 SV=1 |
| Q3SZA1 | 6 | 6 | 235.37 | 9.20E-05 | 6.14E-05 | 3.421838 | RHOF_BOVIN Rho-related GTP-binding protein RhoF OS=Bos taurus GN=RHOF PE=2 SV=1 |
| Q1JPJ2 | 4 | 3 | 140.51 | 9.24E-05 | 6.15E-05 | 2.681412 | XPP1_BOVIN Xaa-Pro aminopeptidase 1 OS=Bos taurus GN=XPNPEP1 PE=2 SV=1 |
| Q2TBG8 | 2 | 2 | 98.94 | 9.29E-05 | 6.16E-05 | 2.912437 | UCHL3_BOVIN Ubiquitin carboxyl-terminal hydrolase isozyme L3 OS=Bos taurus GN=UCHL3 PE=2 SV=1 |
| Q0VCD2 | 3 | 3 | 106.76 | 9.35E-05 | 6.17E-05 | 3.547017 | TBG1_BOVIN Tubulin gamma-1 chain OS=Bos taurus GN=TUBG1 PE=2 SV=1 |
| Q1JPJ8 | 2 | 2 | 69.89 | 9.94E-05 | 6.52E-05 | 7.399914 | THOP1_BOVIN Thimet oligopeptidase OS=Bos taurus GN=THOP1 PE=2 SV=3 |
| Q08E38 | 3 | 3 | 84.37 | 0.0001 | 6.53E-05 | 6.357204 | PRP19_BOVIN Pre-mRNA-processing factor 19 OS=Bos taurus GN=PRPF19 PE=2 SV=1 |
| P10575 | 2 | 2 | 88.52 | 0.000102 | 6.60E-05 | 2.564906 | GLRX1_BOVIN Glutaredoxin-1 OS=Bos taurus GN=GLRX PE=1 SV=3 |
| P00376 | 3 | 3 | 120.61 | 0.000103 | 6.62E-05 | 2.80469 | DYR_BOVIN Dihydrofolate reductase OS=Bos taurus GN=DHFR PE=1 SV=3 |
| P80311 | 7 | 7 | 234.37 | 0.000103 | 6.62E-05 | 2.390845 | PPIB_BOVIN Peptidyl-prolyl cis-trans isomerase B OS=Bos taurus GN=PPIB PE=1 SV=4 |
| Q3SYT7 | 6 | 6 | 185.04 | 0.000107 | 6.79E-05 | 2.453772 | PSMD8_BOVIN 26S proteasome non-ATPase regulatory subunit 8 OS=Bos taurus GN=PSMD8 PE=2 SV=3 |
| Q58DS9 | 8 | 6 | 474.3 | 0.000107 | 6.79E-05 | 2.010919 | RAB5C_BOVIN Ras-related protein Rab-5C OS=Bos taurus GN=RAB5C PE=2 SV=1 |
| Q2KJD0 | 20 | 3 | 1424.87 | 0.000108 | 6.80E-05 | 2.506357 | TBB5_BOVIN Tubulin beta-5 chain OS=Bos taurus GN=TUBB5 PE=2 SV=1 |
| A6H767 | 12 | 11 | 490.65 | 0.00011 | 6.89E-05 | 2.33343 | NP1L1_BOVIN Nucleosome assembly protein 1-like 1 OS=Bos taurus GN=NAP1L1 PE=2 SV=1 |
| P67868 | 2 | 2 | 77.32 | 0.000113 | 7.05E-05 | 11.51556 | CSK2B_BOVIN Casein kinase II subunit beta OS=Bos taurus GN=CSNK2B PE=1 SV=1 |
| Q2TBP0 | 3 | 3 | 68.13 | 0.000114 | 7.08E-05 | 2.626001 | PSB7_BOVIN Proteasome subunit beta type-7 OS=Bos taurus GN=PSMB7 PE=1 SV=1 |
| Q3T102 | 7 | 7 | 207.99 | 0.000117 | 7.22E-05 | 3.954549 | EIF3E_BOVIN Eukaryotic translation initiation factor 3 subunit E OS=Bos taurus GN=EIF3E PE=2 SV=1 |
| Q3T0M7 | 5 | 5 | 204.29 | 0.000124 | 7.58E-05 | 2.548921 | RANG_BOVIN Ran-specific GTPase-activating protein OS=Bos taurus GN=RANBP1 PE=2 SV=1 |
| Q5EA85 | 11 | 11 | 410.85 | 0.000125 | 7.61E-05 | 4.65547 | SEM4A_BOVIN Semaphorin-4A OS=Bos taurus GN=SEMA4A PE=2 SV=1 |
| Q3T0E0 | 3 | 3 | 122.76 | 0.000125 | 7.63E-05 | 3.063736 | ATOX1_BOVIN Copper transport protein ATOX1 OS=Bos taurus GN=ATOX1 PE=3 SV=1 |
| Q5E9B1 | 19 | 16 | 1065.8 | 0.000127 | 7.68E-05 | 2.321438 | LDHB_BOVIN L-lactate dehydrogenase B chain OS=Bos taurus GN=LDHB PE=2 SV=4 |
| P18493 | 12 | 12 | 399.16 | 0.00013 | 7.80E-05 | 3.496838 | PARP1_BOVIN Poly [ADP-ribose] polymerase 1 OS=Bos taurus GN=PARP1 PE=2 SV=2 |
| Q5E984 | 3 | 3 | 162.77 | 0.00013 | 7.80E-05 | 2.199976 | TCTP_BOVIN Translationally-controlled tumor protein OS=Bos taurus GN=TPT1 PE=2 SV=1 |
| P55859 | 13 | 13 | 524.11 | 0.000131 | 7.81E-05 | 2.45908 | PNPH_BOVIN Purine nucleoside phosphorylase OS=Bos taurus GN=PNP PE=1 SV=3 |
| P68102 | 10 | 9 | 463.46 | 0.000131 | 7.81E-05 | 2.348276 | IF2A_BOVIN Eukaryotic translation initiation factor 2 subunit 1 OS=Bos taurus GN=EIF2S1 PE=2 SV=2 |
| Q2KJA1 | 3 | 3 | 114.16 | 0.000132 | 7.81E-05 | 4.394646 | SH3G1_BOVIN Endophilin-A2 OS=Bos taurus GN=SH3GL1 PE=2 SV=1 |
| P51122 | 7 | 2 | 248.85 | 0.000136 | 7.99E-05 | 2.355379 | AN32A_BOVIN Acidic leucine-rich nuclear phosphoprotein 32 family member A OS=Bos taurus GN=ANP32A PE=1 SV=2 |
| Q2YDJ9 | 3 | 3 | 107.1 | 0.00014 | 8.14E-05 | 3.040323 | GMPPB_BOVIN Mannose-1-phosphate guanyltransferase beta OS=Bos taurus GN=GMPPB PE=2 SV=1 |
| P27214 | 12 | 11 | 538.46 | 0.00014 | 8.14E-05 | 2.596604 | ANX11_BOVIN Annexin A11 OS=Bos taurus GN=ANXA11 PE=1 SV=1 |
| Q2KJ37 | 2 | 2 | 72.59 | 0.000147 | 8.38E-05 | 3.051704 | ERD22_BOVIN ER lumen protein-retaining receptor 2 OS=Bos taurus GN=KDELR2 PE=2 SV=1 |
| Q0IIK5 | 6 | 6 | 211.35 | 0.000149 | 8.43E-05 | 6.632443 | DDX1_BOVIN ATP-dependent RNA helicase DDX1 OS=Bos taurus GN=DDX1 PE=2 SV=1 |
| Q28009 | 3 | 3 | 64.67 | 0.000149 | 8.43E-05 | 4.015089 | FUS_BOVIN RNA-binding protein FUS OS=Bos taurus GN=FUS PE=2 SV=2 |
| P10881 | 5 | 5 | 128.48 | 0.00015 | 8.45E-05 | 3.835 | LA_BOVIN Lupus La protein homolog OS=Bos taurus GN=SSB PE=2 SV=2 |
| A6QQ21 | 2 | 2 | 64.08 | 0.000155 | 8.71E-05 | 4.408385 | CSN6_BOVIN COP9 signalosome complex subunit 6 OS=Bos taurus GN=COPS6 PE=2 SV=1 |
| P61223 | 9 | 2 | 544.81 | 0.00016 | 8.88E-05 | 2.524505 | RAP1B_BOVIN Ras-related protein Rap-1b OS=Bos taurus GN=RAP1B PE=2 SV=1 |
| Q5E9G3 | 7 | 6 | 185.06 | 0.000162 | 8.93E-05 | 2.534191 | PSME2_BOVIN Proteasome activator complex subunit 2 OS=Bos taurus GN=PSME2 PE=1 SV=3 |
| Q2HJ58 | 9 | 7 | 318.92 | 0.000163 | 8.93E-05 | 2.870753 | PRPS1_BOVIN Ribose-phosphate pyrophosphokinase 1 OS=Bos taurus GN=PRPS1 PE=2 SV=3 |
| Q2HJI8 | 10 | 4 | 551.33 | 0.000163 | 8.93E-05 | 2.543663 | RAB8B_BOVIN Ras-related protein Rab-8B OS=Bos taurus GN=RAB8B PE=2 SV=1 |
| Q3MHM5 | 20 | 3 | 1258.21 | 0.000163 | 8.95E-05 | 2.427987 | TBB4B_BOVIN Tubulin beta-4B chain OS=Bos taurus GN=TUBB4B PE=2 SV=1 |
| Q3T108 | 2 | 2 | 114.15 | 0.000171 | 9.29E-05 | 2.01194 | PSB4_BOVIN Proteasome subunit beta type-4 OS=Bos taurus GN=PSMB4 PE=1 SV=1 |
| Q3T199 | 5 | 5 | 200.19 | 0.000174 | 9.38E-05 | 3.448946 | RS23_BOVIN 40S ribosomal protein S23 OS=Bos taurus GN=RPS23 PE=2 SV=1 |
| Q0VCK5 | 5 | 4 | 183.98 | 0.000173 | 9.38E-05 | 3.2971 | AP2A2_BOVIN AP-2 complex subunit alpha-2 OS=Bos taurus GN=AP2A2 PE=1 SV=1 |
| Q2T9L8 | 6 | 6 | 201.25 | 0.000183 | 9.73E-05 | 2.604101 | SYMC_BOVIN Methionine--tRNA ligase, cytoplasmic OS=Bos taurus GN=MARS PE=2 SV=1 |
| Q58DQ3 | 9 | 9 | 459.18 | 0.000183 | 9.73E-05 | 2.35684 | RL6_BOVIN 60S ribosomal protein L6 OS=Bos taurus GN=RPL6 PE=2 SV=3 |
| Q28205 | 4 | 3 | 93.95 | 0.00019 | 9.96E-05 | 8.509345 | TBCD_BOVIN Tubulin-specific chaperone D OS=Bos taurus GN=TBCD PE=1 SV=1 |
| Q6EWQ7 | 8 | 8 | 547.73 | 0.000189 | 9.96E-05 | 2.4062 | IF5A1_BOVIN Eukaryotic translation initiation factor 5A-1 OS=Bos taurus GN=EIF5A PE=2 SV=3 |
| Q3T025 | 4 | 4 | 134.78 | 0.000191 | 9.98E-05 | 2.751545 | RL17_BOVIN 60S ribosomal protein L17 OS=Bos taurus GN=RPL17 PE=2 SV=3 |
| Q3SWY3 | 13 | 12 | 572.67 | 0.000192 | 1.00E-04 | 2.780055 | IMDH2_BOVIN Inosine-5'-monophosphate dehydrogenase 2 OS=Bos taurus GN=IMPDH2 PE=2 SV=1 |
| P63243 | 16 | 16 | 863.23 | 0.000196 | 0.000101 | 2.318555 | GBLP_BOVIN Guanine nucleotide-binding protein subunit beta-2-like 1 OS=Bos taurus GN=GNB2L1 PE=2 SV=3 |
| Q08DM8 | 4 | 4 | 193.66 | 0.000208 | 0.000107 | 2.033836 | TSN_BOVIN Translin OS=Bos taurus GN=TSN PE=2 SV=1 |
| Q3T0W9 | 3 | 2 | 144.74 | 0.000209 | 0.000107 | 2.529718 | RL19_BOVIN 60S ribosomal protein L19 OS=Bos taurus GN=RPL19 PE=2 SV=1 |
| Q29RK4 | 5 | 5 | 166.94 | 0.000211 | 0.000108 | 2.5335 | RD23B_BOVIN UV excision repair protein RAD23 homolog B OS=Bos taurus GN=RAD23B PE=2 SV=1 |
| Q9XSI3 | 6 | 6 | 192.57 | 0.000214 | 0.000108 | 2.258262 | RL10_BOVIN 60S ribosomal protein L10 OS=Bos taurus GN=RPL10 PE=2 SV=4 |
| A6H7I5 | 8 | 8 | 269.53 | 0.000216 | 0.000109 | 2.565816 | DYN2_BOVIN Dynamin-2 OS=Bos taurus GN=DNM2 PE=2 SV=1 |
| P41541 | 3 | 3 | 100.83 | 0.000226 | 0.000113 | 3.172434 | USO1_BOVIN General vesicular transport factor p115 OS=Bos taurus GN=USO1 PE=1 SV=1 |
| Q2TBQ5 | 9 | 9 | 439.16 | 0.000228 | 0.000114 | 2.467333 | RL7A_BOVIN 60S ribosomal protein L7a OS=Bos taurus GN=RPL7A PE=2 SV=3 |
| Q3T122 | 8 | 7 | 253.74 | 0.000232 | 0.000115 | 2.706996 | EIF3D_BOVIN Eukaryotic translation initiation factor 3 subunit D OS=Bos taurus GN=EIF3D PE=2 SV=1 |
| Q29465 | 12 | 11 | 414.59 | 0.000232 | 0.000115 | 2.399614 | SYYC_BOVIN Tyrosine--tRNA ligase, cytoplasmic OS=Bos taurus GN=YARS PE=2 SV=4 |
| Q2NKZ1 | 20 | 20 | 1148.15 | 0.000233 | 0.000115 | 2.155177 | TCPH_BOVIN T-complex protein 1 subunit eta OS=Bos taurus GN=CCT7 PE=1 SV=1 |
| Q58DA7 | 4 | 4 | 90.85 | 0.000237 | 0.000116 | 2.371543 | GLRX3_BOVIN Glutaredoxin-3 OS=Bos taurus GN=GLRX3 PE=2 SV=1 |
| P07107 | 3 | 3 | 191.99 | 0.000252 | 0.000123 | 2.001451 | ACBP_BOVIN Acyl-CoA-binding protein OS=Bos taurus GN=DBI PE=1 SV=2 |
| Q58DW5 | 7 | 7 | 257.4 | 0.000258 | 0.000124 | 2.423714 | RL5_BOVIN 60S ribosomal protein L5 OS=Bos taurus GN=RPL5 PE=2 SV=3 |
| Q3B7M5 | 3 | 2 | 118.36 | 0.000259 | 0.000125 | 2.371165 | LASP1_BOVIN LIM and SH3 domain protein 1 OS=Bos taurus GN=LASP1 PE=2 SV=1 |
| Q1JQB2 | 2 | 2 | 60.79 | 0.000266 | 0.000127 | 3.252712 | BUB3_BOVIN Mitotic checkpoint protein BUB3 OS=Bos taurus GN=BUB3 PE=2 SV=1 |
| P60519 | 2 | 2 | 60.52 | 0.000278 | 0.000132 | 2.690965 | GBRL2_BOVIN Gamma-aminobutyric acid receptor-associated protein-like 2 OS=Bos taurus GN=GABARAPL2 PE=1 SV=1 |
| Q3SZI4 | 15 | 10 | 728.49 | 0.00028 | 0.000133 | 2.185544 | 1433T_BOVIN 14-3-3 protein theta OS=Bos taurus GN=YWHAQ PE=2 SV=1 |
| Q56JV3 | 2 | 2 | 55.2 | 0.000287 | 0.000135 | 5.204721 | RHEB_BOVIN GTP-binding protein Rheb OS=Bos taurus GN=RHEB PE=2 SV=1 |
| Q3T0K2 | 26 | 26 | 1349.43 | 0.000287 | 0.000135 | 2.245148 | TCPG_BOVIN T-complex protein 1 subunit gamma OS=Bos taurus GN=CCT3 PE=1 SV=1 |
| P61356 | 5 | 5 | 174.86 | 0.000293 | 0.000137 | 2.217739 | RL27_BOVIN 60S ribosomal protein L27 OS=Bos taurus GN=RPL27 PE=2 SV=2 |
| P00432 | 4 | 4 | 120.64 | 0.000292 | 0.000137 | 2.217287 | CATA_BOVIN Catalase OS=Bos taurus GN=CAT PE=1 SV=3 |
| Q08DP3 | 2 | 2 | 36.88 | 0.000297 | 0.000138 | 4.094834 | RELL1_BOVIN RELT-like protein 1 OS=Bos taurus GN=RELL1 PE=2 SV=1 |
| Q2HJ60 | 10 | 8 | 530.41 | 0.000322 | 0.000148 | 2.049448 | ROA2_BOVIN Heterogeneous nuclear ribonucleoproteins A2/B1 OS=Bos taurus GN=HNRNPA2B1 PE=2 SV=1 |
| A4FV97 | 2 | 2 | 51.24 | 0.000323 | 0.000148 | 3.65394 | RL1D1_BOVIN Ribosomal L1 domain-containing protein 1 OS=Bos taurus GN=RSL1D1 PE=2 SV=1 |
| Q58DA0 | 3 | 3 | 115.26 | 0.000324 | 0.000149 | 4.710131 | PSMD4_BOVIN 26S proteasome non-ATPase regulatory subunit 4 OS=Bos taurus GN=PSMD4 PE=2 SV=1 |
| P13696 | 6 | 6 | 400.92 | 0.000327 | 0.000149 | 2.036204 | PEBP1_BOVIN Phosphatidylethanolamine-binding protein 1 OS=Bos taurus GN=PEBP1 PE=1 SV=2 |
| P10096 | 16 | 15 | 1046.53 | 0.000338 | 0.000153 | 2.1127 | G3P_BOVIN Glyceraldehyde-3-phosphate dehydrogenase OS=Bos taurus GN=GAPDH PE=1 SV=4 |
| Q0IIM3 | 22 | 21 | 1077.84 | 0.000347 | 0.000156 | 3.014523 | HS105_BOVIN Heat shock protein 105 kDa OS=Bos taurus GN=HSPH1 PE=2 SV=1 |
| P04896 | 12 | 11 | 656.42 | 0.000349 | 0.000157 | 2.293495 | GNAS2_BOVIN Guanine nucleotide-binding protein G(s) subunit alpha isoforms short OS=Bos taurus GN=GNAS PE=1 SV=1 |
| A1L5A6 | 2 | 2 | 49.34 | 0.000374 | 0.000167 | 7.236878 | ADRM1_BOVIN Proteasomal ubiquitin receptor ADRM1 OS=Bos taurus GN=ADRM1 PE=2 SV=1 |
| Q5E9A1 | 4 | 3 | 265.47 | 0.000377 | 0.000167 | 2.813395 | NACA_BOVIN Nascent polypeptide-associated complex subunit alpha OS=Bos taurus GN=NACA PE=1 SV=1 |
| Q2HJG5 | 6 | 6 | 242.3 | 0.000392 | 0.000174 | 2.452184 | VPS35_BOVIN Vacuolar protein sorting-associated protein 35 OS=Bos taurus GN=VPS35 PE=2 SV=1 |
| A5PK63 | 3 | 3 | 106.67 | 0.000402 | 0.000177 | 2.200594 | RS17_BOVIN 40S ribosomal protein S17 OS=Bos taurus GN=RPS17 PE=2 SV=1 |
| P68138 | 18 | 3 | 1076.62 | 0.000405 | 0.000178 | 2.092687 | ACTS_BOVIN Actin, alpha skeletal muscle OS=Bos taurus GN=ACTA1 PE=1 SV=1 |
| A3KN12 | 5 | 5 | 255.02 | 0.000422 | 0.000184 | 3.351605 | PUR8_BOVIN Adenylosuccinate lyase OS=Bos taurus GN=ADSL PE=2 SV=1 |
| Q1RMH8 | 4 | 4 | 124.45 | 0.000432 | 0.000188 | 2.239231 | SNX3_BOVIN Sorting nexin-3 OS=Bos taurus GN=SNX3 PE=2 SV=3 |
| O18789 | 11 | 11 | 533.97 | 0.000442 | 0.000192 | 2.169223 | RS2_BOVIN 40S ribosomal protein S2 OS=Bos taurus GN=RPS2 PE=2 SV=2 |
| Q5KR48 | 9 | 3 | 381.15 | 0.00046 | 0.000197 | 2.587962 | TPM2_BOVIN Tropomyosin beta chain OS=Bos taurus GN=TPM2 PE=2 SV=1 |
| Q3MHY1 | 3 | 3 | 104.34 | 0.00048 | 0.000204 | 3.317455 | CSRP1_BOVIN Cysteine and glycine-rich protein 1 OS=Bos taurus GN=CSRP1 PE=2 SV=3 |
| Q2HJ33 | 6 | 5 | 201.15 | 0.00048 | 0.000204 | 2.162503 | OLA1_BOVIN Obg-like ATPase 1 OS=Bos taurus GN=OLA1 PE=2 SV=1 |
| Q3B7L6 | 4 | 4 | 109.05 | 0.000496 | 0.00021 | 2.44519 | SRSF6_BOVIN Serine/arginine-rich splicing factor 6 OS=Bos taurus GN=SRSF6 PE=2 SV=1 |
| P26452 | 7 | 7 | 430.44 | 0.000506 | 0.000212 | 2.514932 | RSSA_BOVIN 40S ribosomal protein SA OS=Bos taurus GN=RPSA PE=2 SV=4 |
| Q3SZC2 | 2 | 2 | 134.65 | 0.000507 | 0.000212 | 2.397612 | PSB9_BOVIN Proteasome subunit beta type-9 OS=Bos taurus GN=PSMB9 PE=1 SV=1 |
| Q3SYU7 | 4 | 4 | 187.8 | 0.000527 | 0.00022 | 2.451391 | TNPO1_BOVIN Transportin-1 OS=Bos taurus GN=TNPO1 PE=1 SV=2 |
| Q28036 | 2 | 2 | 40.16 | 0.000544 | 0.000225 | 10.32611 | SL9A1_BOVIN Sodium/hydrogen exchanger 1 OS=Bos taurus GN=SLC9A1 PE=2 SV=1 |
| Q5E973 | 6 | 6 | 383.98 | 0.000543 | 0.000225 | 2.095399 | RL18_BOVIN 60S ribosomal protein L18 OS=Bos taurus GN=RPL18 PE=2 SV=3 |
| Q08DA1 | 36 | 21 | 2660.28 | 0.000544 | 0.000225 | 2.031851 | AT1A1_BOVIN Sodium/potassium-transporting ATPase subunit alpha-1 OS=Bos taurus GN=ATP1A1 PE=2 SV=1 |
| P00517 | 4 | 3 | 122.21 | 0.000549 | 0.000226 | 3.37221 | KAPCA_BOVIN cAMP-dependent protein kinase catalytic subunit alpha OS=Bos taurus GN=PRKACA PE=1 SV=3 |
| P67774 | 5 | 5 | 203.49 | 0.000574 | 0.000236 | 2.155849 | PP2AA_BOVIN Serine/threonine-protein phosphatase 2A catalytic subunit alpha isoform OS=Bos taurus GN=PPP2CA PE=1 SV=1 |
| Q3T0D5 | 5 | 5 | 316.72 | 0.000597 | 0.000244 | 2.372297 | RL30_BOVIN 60S ribosomal protein L30 OS=Bos taurus GN=RPL30 PE=3 SV=3 |
| Q17QN6 | 7 | 7 | 325.54 | 0.000601 | 0.000244 | 2.159323 | EM55_BOVIN 55 kDa erythrocyte membrane protein OS=Bos taurus GN=MPP1 PE=1 SV=1 |
| Q3ZBD0 | 4 | 4 | 134.22 | 0.000647 | 0.00026 | 3.23735 | PSMD7_BOVIN 26S proteasome non-ATPase regulatory subunit 7 OS=Bos taurus GN=PSMD7 PE=2 SV=1 |
| Q3T0V4 | 8 | 8 | 272.5 | 0.000648 | 0.00026 | 2.218375 | RS11_BOVIN 40S ribosomal protein S11 OS=Bos taurus GN=RPS11 PE=2 SV=3 |
| Q07130 | 5 | 5 | 149.16 | 0.000665 | 0.000265 | 2.830321 | UGPA_BOVIN UTP--glucose-1-phosphate uridylyltransferase OS=Bos taurus GN=UGP2 PE=1 SV=2 |
| Q2HJH2 | 7 | 4 | 441.06 | 0.000668 | 0.000265 | 2.149761 | RAB1B_BOVIN Ras-related protein Rab-1B OS=Bos taurus GN=RAB1B PE=2 SV=1 |
| Q3ZBG5 | 2 | 2 | 66.07 | 0.000725 | 0.000286 | 3.711906 | BAG2_BOVIN BAG family molecular chaperone regulator 2 OS=Bos taurus PE=2 SV=1 |
| P61603 | 2 | 2 | 96.14 | 0.00074 | 0.00029 | 2.855803 | CH10_BOVIN 10 kDa heat shock protein, mitochondrial OS=Bos taurus GN=HSPE1 PE=3 SV=2 |
| Q3ZCF3 | 2 | 2 | 41.09 | 0.000743 | 0.00029 | 2.157336 | SKP1_BOVIN S-phase kinase-associated protein 1 OS=Bos taurus GN=SKP1 PE=2 SV=1 |
| Q58DR5 | 4 | 4 | 247.67 | 0.000786 | 0.000304 | 3.304772 | SCAM3_BOVIN Secretory carrier-associated membrane protein 3 OS=Bos taurus GN=SCAMP3 PE=2 SV=1 |
| Q28046 | 8 | 7 | 290.84 | 0.000786 | 0.000304 | 2.540535 | ADSV_BOVIN Adseverin OS=Bos taurus GN=SCIN PE=1 SV=1 |
| P00514 | 3 | 3 | 95.15 | 0.000797 | 0.000306 | 2.845032 | KAP0_BOVIN cAMP-dependent protein kinase type I-alpha regulatory subunit OS=Bos taurus GN=PRKAR1A PE=1 SV=2 |
| Q95140 | 13 | 13 | 580.15 | 0.000797 | 0.000306 | 2.325632 | RLA0_BOVIN 60S acidic ribosomal protein P0 OS=Bos taurus GN=RPLP0 PE=2 SV=3 |
| P56425 | 2 | 2 | 96.59 | 0.000813 | 0.000312 | 2.95046 | CTHL7_BOVIN Cathelicidin-7 OS=Bos taurus GN=CATHL7 PE=2 SV=1 |
| Q3T0W4 | 2 | 2 | 69.82 | 0.000821 | 0.000314 | 4.57186 | PP1R7_BOVIN Protein phosphatase 1 regulatory subunit 7 OS=Bos taurus GN=PPP1R7 PE=1 SV=1 |
| Q2KIA5 | 4 | 4 | 107.87 | 0.000848 | 0.000321 | 2.881513 | DNM1L_BOVIN Dynamin-1-like protein OS=Bos taurus GN=DNM1L PE=2 SV=1 |
| Q2KII4 | 2 | 2 | 81.85 | 0.000852 | 0.000321 | 2.788295 | ELOC_BOVIN Transcription elongation factor B polypeptide 1 OS=Bos taurus GN=TCEB1 PE=3 SV=1 |
| Q3SYZ4 | 16 | 16 | 541.57 | 0.000851 | 0.000321 | 2.317502 | SYDC_BOVIN Aspartate--tRNA ligase, cytoplasmic OS=Bos taurus GN=DARS PE=2 SV=1 |
| Q3SZF3 | 4 | 3 | 242.27 | 0.000852 | 0.000321 | 2.197792 | HNRH2_BOVIN Heterogeneous nuclear ribonucleoprotein H2 OS=Bos taurus GN=HNRNPH2 PE=2 SV=1 |
| Q3T148 | 4 | 4 | 140.24 | 0.000894 | 0.000334 | 2.758851 | EIF3M_BOVIN Eukaryotic translation initiation factor 3 subunit M OS=Bos taurus GN=EIF3M PE=2 SV=2 |
| P43033 | 4 | 3 | 109.16 | 0.000918 | 0.000341 | 2.046229 | LIS1_BOVIN Platelet-activating factor acetylhydrolase IB subunit alpha OS=Bos taurus GN=PAFAH1B1 PE=1 SV=2 |
| Q58DM3 | 2 | 2 | 132.03 | 0.00099 | 0.000366 | 2.292547 | CD53_BOVIN Leukocyte surface antigen CD53 OS=Bos taurus GN=CD53 PE=2 SV=1 |
| P17248 | 3 | 3 | 147.71 | 0.001036 | 0.000378 | 2.643963 | SYWC_BOVIN Tryptophan--tRNA ligase, cytoplasmic OS=Bos taurus GN=WARS PE=1 SV=3 |
| Q9XSK7 | 3 | 3 | 126.66 | 0.001051 | 0.000382 | 2.577156 | HDGF_BOVIN Hepatoma-derived growth factor OS=Bos taurus GN=HDGF PE=2 SV=1 |
| Q2KIR1 | 3 | 3 | 95.01 | 0.001047 | 0.000382 | 2.258346 | SNRPA_BOVIN U1 small nuclear ribonucleoprotein A OS=Bos taurus GN=SNRPA PE=2 SV=1 |
| O97594 | 5 | 4 | 108.9 | 0.001082 | 0.000391 | 2.136409 | SMC3_BOVIN Structural maintenance of chromosomes protein 3 OS=Bos taurus GN=SMC3 PE=1 SV=1 |
| Q0V8B7 | 3 | 3 | 67.68 | 0.00109 | 0.000393 | 3.108111 | MCM5_BOVIN DNA replication licensing factor MCM5 OS=Bos taurus GN=MCM5 PE=2 SV=1 |
| Q3T0Q4 | 7 | 5 | 470.29 | 0.001102 | 0.000396 | 2.056852 | NDKB_BOVIN Nucleoside diphosphate kinase B OS=Bos taurus GN=NME2 PE=1 SV=1 |
| Q3ZBT1 | 38 | 38 | 1806.39 | 0.001142 | 0.000409 | 2.040048 | TERA_BOVIN Transitional endoplasmic reticulum ATPase OS=Bos taurus GN=VCP PE=2 SV=1 |
| Q2KIW9 | 2 | 2 | 69.01 | 0.001149 | 0.00041 | 3.260506 | KCY_BOVIN UMP-CMP kinase OS=Bos taurus GN=CMPK1 PE=2 SV=2 |
| Q32PB9 | 3 | 2 | 84.14 | 0.001189 | 0.000418 | 2.737504 | RL38_BOVIN 60S ribosomal protein L38 OS=Bos taurus GN=RPL38 PE=3 SV=4 |
| P04973 | 4 | 4 | 135.22 | 0.001189 | 0.000418 | 2.097093 | CLCA_BOVIN Clathrin light chain A OS=Bos taurus GN=CLTA PE=1 SV=1 |
| Q5E966 | 6 | 6 | 241.45 | 0.001192 | 0.000418 | 2.264471 | EIF3I_BOVIN Eukaryotic translation initiation factor 3 subunit I OS=Bos taurus GN=EIF3I PE=2 SV=1 |
| Q32LG3 | 2 | 2 | 86.94 | 0.001223 | 0.000427 | 3.766053 | MDHM_BOVIN Malate dehydrogenase, mitochondrial OS=Bos taurus GN=MDH2 PE=1 SV=1 |
| Q17QN3 | 3 | 3 | 99.07 | 0.001313 | 0.000452 | 2.947039 | RSMN_BOVIN Small nuclear ribonucleoprotein-associated protein N OS=Bos taurus GN=SNRPN PE=2 SV=1 |
| Q5E9Q4 | 2 | 2 | 48.84 | 0.001321 | 0.000453 | 2.38105 | NECP2_BOVIN Adaptin ear-binding coat-associated protein 2 OS=Bos taurus GN=NECAP2 PE=2 SV=1 |
| Q3T105 | 2 | 2 | 37.2 | 0.001455 | 0.000492 | 11.03923 | GALE_BOVIN UDP-glucose 4-epimerase OS=Bos taurus GN=GALE PE=2 SV=2 |
| Q3SZ43 | 3 | 3 | 132.71 | 0.001461 | 0.000493 | 2.583321 | UB2V2_BOVIN Ubiquitin-conjugating enzyme E2 variant 2 OS=Bos taurus GN=UBE2V2 PE=2 SV=3 |
| A7Z035 | 2 | 2 | 55.91 | 0.001538 | 0.000518 | 2.487444 | EPN4_BOVIN Clathrin interactor 1 OS=Bos taurus GN=CLINT1 PE=2 SV=1 |
| A0JN39 | 3 | 3 | 111.82 | 0.001545 | 0.000518 | 2.623166 | COPB_BOVIN Coatomer subunit beta OS=Bos taurus GN=COPB1 PE=1 SV=1 |
| P79103 | 10 | 9 | 500.08 | 0.001622 | 0.000537 | 2.023532 | RS4_BOVIN 40S ribosomal protein S4 OS=Bos taurus GN=RPS4 PE=2 SV=3 |
| Q3T057 | 3 | 3 | 216.66 | 0.001682 | 0.000554 | 2.475048 | RL23_BOVIN 60S ribosomal protein L23 OS=Bos taurus GN=RPL23 PE=2 SV=2 |
| P10790 | 7 | 7 | 296.1 | 0.001681 | 0.000554 | 2.127796 | FABPH_BOVIN Fatty acid-binding protein, heart OS=Bos taurus GN=FABP3 PE=1 SV=2 |
| Q3T0S6 | 4 | 4 | 194.46 | 0.001713 | 0.000563 | 2.072603 | RL8_BOVIN 60S ribosomal protein L8 OS=Bos taurus GN=RPL8 PE=2 SV=3 |
| Q0IIG8 | 4 | 4 | 110.93 | 0.001765 | 0.000577 | 2.245389 | RAB18_BOVIN Ras-related protein Rab-18 OS=Bos taurus GN=RAB18 PE=2 SV=1 |
| Q5E988 | 5 | 5 | 222.98 | 0.001808 | 0.000589 | 2.164497 | RS5_BOVIN 40S ribosomal protein S5 OS=Bos taurus GN=RPS5 PE=2 SV=3 |
| Q56K14 | 2 | 2 | 70.74 | 0.001991 | 0.000646 | 2.155428 | RLA1_BOVIN 60S acidic ribosomal protein P1 OS=Bos taurus GN=RPLP1 PE=3 SV=1 |
| A2VE14 | 2 | 2 | 72.25 | 0.002012 | 0.000651 | 3.744507 | SAE1_BOVIN SUMO-activating enzyme subunit 1 OS=Bos taurus GN=SAE1 PE=2 SV=1 |
| P45879 | 2 | 2 | 85.9 | 0.002085 | 0.000672 | 5.003046 | VDAC1_BOVIN Voltage-dependent anion-selective channel protein 1 OS=Bos taurus GN=VDAC1 PE=1 SV=3 |
| Q8HYI9 | 2 | 2 | 56.74 | 0.002092 | 0.000673 | 2.918086 | PFD5_BOVIN Prefoldin subunit 5 OS=Bos taurus GN=PFDN5 PE=2 SV=1 |
| Q0VD53 | 5 | 5 | 132.09 | 0.002101 | 0.000674 | 2.441833 | VP26A_BOVIN Vacuolar protein sorting-associated protein 26A OS=Bos taurus GN=VPS26A PE=2 SV=1 |
| Q3SZQ6 | 3 | 3 | 122.07 | 0.002332 | 0.000736 | 2.608403 | RL32_BOVIN 60S ribosomal protein L32 OS=Bos taurus GN=RPL32 PE=2 SV=3 |
| Q3SYR7 | 4 | 4 | 136.83 | 0.00242 | 0.000759 | 2.582689 | RL9_BOVIN 60S ribosomal protein L9 OS=Bos taurus GN=RPL9 PE=2 SV=1 |
| P11064 | 2 | 2 | 104.16 | 0.002689 | 0.00083 | 2.999731 | PPAC_BOVIN Low molecular weight phosphotyrosine protein phosphatase OS=Bos taurus GN=ACP1 PE=1 SV=3 |
| Q6Q137 | 8 | 7 | 296.78 | 0.002723 | 0.000835 | 2.072005 | SEPT7_BOVIN Septin-7 OS=Bos taurus GN=SEPT7 PE=2 SV=2 |
| O18956 | 3 | 3 | 116.49 | 0.00278 | 0.00085 | 2.013348 | ENTP1_BOVIN Ectonucleoside triphosphate diphosphohydrolase 1 OS=Bos taurus GN=ENTPD1 PE=1 SV=1 |
| P00727 | 7 | 7 | 235.06 | 0.003042 | 0.000916 | 2.474485 | AMPL_BOVIN Cytosol aminopeptidase OS=Bos taurus GN=LAP3 PE=1 SV=3 |
| Q2TBQ8 | 2 | 2 | 33.62 | 0.003166 | 0.000946 | 3.171426 | 6PGL_BOVIN 6-phosphogluconolactonase OS=Bos taurus GN=PGLS PE=2 SV=1 |
| P11019 | 4 | 4 | 149.28 | 0.003166 | 0.000946 | 2.386248 | VATE1_BOVIN V-type proton ATPase subunit E 1 OS=Bos taurus GN=ATP6V1E1 PE=2 SV=1 |
| P52193 | 6 | 6 | 162.14 | 0.003205 | 0.000955 | 2.116182 | CALR_BOVIN Calreticulin OS=Bos taurus GN=CALR PE=1 SV=2 |
| P48734 | 5 | 4 | 194.23 | 0.003319 | 0.000982 | 2.045587 | CDK1_BOVIN Cyclin-dependent kinase 1 OS=Bos taurus GN=CDK1 PE=2 SV=2 |
| Q3ZC12 | 2 | 2 | 78.75 | 0.003555 | 0.001044 | 3.091525 | EIF3G_BOVIN Eukaryotic translation initiation factor 3 subunit G OS=Bos taurus GN=EIF3G PE=2 SV=1 |
| Q3SYS6 | 5 | 5 | 176.31 | 0.003724 | 0.001087 | 2.680048 | CHP1_BOVIN Calcineurin B homologous protein 1 OS=Bos taurus GN=CHP1 PE=2 SV=1 |
| P01252 | 4 | 4 | 155.61 | 0.003825 | 0.001114 | 2.024055 | PTMA_BOVIN Prothymosin alpha OS=Bos taurus GN=PTMA PE=1 SV=2 |
| A7MAZ5 | 4 | 4 | 171.76 | 0.003838 | 0.001116 | 2.467914 | H13_BOVIN Histone H1.3 OS=Bos taurus GN=HIST1H1D PE=1 SV=1 |
| Q3SZA0 | 2 | 2 | 82.56 | 0.005074 | 0.001438 | 3.210996 | CSN4_BOVIN COP9 signalosome complex subunit 4 OS=Bos taurus GN=COPS4 PE=2 SV=1 |
| Q76I82 | 2 | 2 | 70.47 | 0.00646 | 0.001774 | 2.312873 | RS15A_BOVIN 40S ribosomal protein S15a OS=Bos taurus GN=RPS15A PE=2 SV=1 |
| P53793 | 3 | 3 | 161.27 | 0.006727 | 0.00183 | 2.860011 | SC5A3_BOVIN Sodium/myo-inositol cotransporter OS=Bos taurus GN=SLC5A3 PE=3 SV=1 |
| Q8SPU8 | 2 | 2 | 42.83 | 0.007348 | 0.001985 | 2.285229 | DHRS4_BOVIN Dehydrogenase/reductase SDR family member 4 OS=Bos taurus GN=DHRS4 PE=2 SV=2 |
| Q29RL9 | 2 | 2 | 76.32 | 0.007693 | 0.00207 | 2.341932 | TCEA1_BOVIN Transcription elongation factor A protein 1 OS=Bos taurus GN=TCEA1 PE=2 SV=1 |
| A6QQ91 | 10 | 9 | 307.27 | 0.008133 | 0.002165 | 2.119152 | RASL3_BOVIN RAS protein activator like-3 OS=Bos taurus GN=RASAL3 PE=2 SV=1 |
| Q17QQ2 | 4 | 4 | 123.39 | 0.009812 | 0.002572 | 2.36643 | TPMT_BOVIN Thiopurine S-methyltransferase OS=Bos taurus GN=TPMT PE=2 SV=1 |
| P00829 | 3 | 3 | 148.24 | 0.010485 | 0.002722 | 2.676366 | ATPB_BOVIN ATP synthase subunit beta, mitochondrial OS=Bos taurus GN=ATP5B PE=1 SV=2 |
| Q2HJ81 | 10 | 2 | 579.52 | 0.010923 | 0.002808 | 2.798639 | TBB6_BOVIN Tubulin beta-6 chain OS=Bos taurus GN=TUBB6 PE=2 SV=1 |
| A4FV08 | 6 | 6 | 189.15 | 0.011823 | 0.003019 | 2.008956 | GNPI1_BOVIN Glucosamine-6-phosphate isomerase 1 OS=Bos taurus GN=GNPDA1 PE=2 SV=1 |
| Q2KIZ8 | 3 | 3 | 112.01 | 0.013699 | 0.003464 | 2.069531 | MCM6_BOVIN DNA replication licensing factor MCM6 OS=Bos taurus GN=MCM6 PE=2 SV=1 |
| A6QR46 | 3 | 2 | 145.92 | 0.01397 | 0.003518 | 2.341025 | RAB6B_BOVIN Ras-related protein Rab-6B OS=Bos taurus GN=RAB6B PE=2 SV=1 |
| Q29451 | 3 | 3 | 89.69 | 0.015126 | 0.003773 | 2.269065 | MA2B1_BOVIN Lysosomal alpha-mannosidase OS=Bos taurus GN=MAN2B1 PE=1 SV=4 |
| Q3ZCA2 | 2 | 2 | 57.81 | 0.016784 | 0.004137 | 2.499303 | CPSF5_BOVIN Cleavage and polyadenylation specificity factor subunit 5 OS=Bos taurus GN=NUDT21 PE=2 SV=1 |
| Q3ZCH0 | 5 | 4 | 168.86 | 0.017433 | 0.004271 | 2.015847 | GRP75_BOVIN Stress-70 protein, mitochondrial OS=Bos taurus GN=HSPA9 PE=2 SV=1 |
| Q3SZ20 | 2 | 2 | 52.98 | 0.029063 | 0.006794 | 2.476253 | GLYM_BOVIN Serine hydroxymethyltransferase, mitochondrial OS=Bos taurus GN=SHMT2 PE=2 SV=1 |
| Q3ZCC9 | 2 | 2 | 85.6 | 0.029498 | 0.006877 | 2.19942 | SEC13_BOVIN Protein SEC13 homolog OS=Bos taurus GN=SEC13 PE=2 SV=1 |
| Q3T0Q6 | 5 | 5 | 155.68 | 0.041489 | 0.009379 | 2.326779 | CNBP_BOVIN Cellular nucleic acid-binding protein OS=Bos taurus GN=CNBP PE=2 SV=1 |

**84 Proteins up-regulated in BL20 EV**

| **Accession** | **Peptide count** | **Unique peptides** | **Confidence score** | **Anova (p)** | **q Value** | **Max fold change** | **Description** |
| --- | --- | --- | --- | --- | --- | --- | --- |
| Q28125 | 3 | 2 | 119.37 | 4.84E-06 | 8.70E-06 | 6.375331 | ICAM3_BOVIN Intercellular adhesion molecule 3 OS=Bos taurus GN=ICAM3 PE=2 SV=1 |
| Q3T149 | 10 | 10 | 428.62 | 3.29E-09 | 8.72E-08 | 7.071143 | HSPB1_BOVIN Heat shock protein beta-1 OS=Bos taurus GN=HSPB1 PE=2 SV=1 |
| P61625 | 17 | 17 | 560.52 | 9.50E-07 | 3.04E-06 | 5.142964 | ITAL_BOVIN Integrin alpha-L OS=Bos taurus GN=ITGAL PE=2 SV=1 |
| A7E3W2 | 10 | 10 | 421.55 | 2.01E-06 | 4.92E-06 | 2.250387 | LG3BP_BOVIN Galectin-3-binding protein OS=Bos taurus GN=LGALS3BP PE=1 SV=1 |
| Q3ZBD7 | 10 | 10 | 351.09 | 3.61E-06 | 7.37E-06 | 2.91413 | G6PI_BOVIN Glucose-6-phosphate isomerase OS=Bos taurus GN=GPI PE=2 SV=4 |
| P32592 | 15 | 15 | 484.06 | 4.71E-06 | 8.70E-06 | 3.100648 | ITB2_BOVIN Integrin beta-2 OS=Bos taurus GN=ITGB2 PE=1 SV=1 |
| P02081 | 14 | 9 | 1390.54 | 5.47E-06 | 9.22E-06 | 2.890319 | HBBF_BOVIN Hemoglobin fetal subunit beta OS=Bos taurus PE=1 SV=1 |
| P22226 | 4 | 4 | 173.6 | 6.40E-06 | 1.05E-05 | 3.103312 | CTHL1_BOVIN Cathelicidin-1 OS=Bos taurus GN=CATHL1 PE=1 SV=2 |
| Q29443 | 43 | 43 | 3340.16 | 8.20E-06 | 1.25E-05 | 2.709379 | TRFE_BOVIN Serotransferrin OS=Bos taurus GN=TF PE=2 SV=1 |
| Q2TBU0 | 19 | 19 | 796.6 | 2.54E-05 | 2.53E-05 | 3.75274 | HPT_BOVIN Haptoglobin OS=Bos taurus GN=HP PE=2 SV=1 |
| Q05443 | 13 | 13 | 663.54 | 3.33E-05 | 3.03E-05 | 2.462829 | LUM_BOVIN Lumican OS=Bos taurus GN=LUM PE=1 SV=1 |
| Q29RQ1 | 31 | 31 | 1398.72 | 5.92E-05 | 4.67E-05 | 2.311009 | CO7_BOVIN Complement component C7 OS=Bos taurus GN=C7 PE=2 SV=1 |
| Q2UVX4 | 98 | 97 | 5945.26 | 6.15E-05 | 4.78E-05 | 2.319348 | CO3_BOVIN Complement C3 OS=Bos taurus GN=C3 PE=1 SV=2 |
| Q3SZR3 | 12 | 11 | 632.58 | 6.92E-05 | 5.16E-05 | 4.103648 | A1AG_BOVIN Alpha-1-acid glycoprotein OS=Bos taurus GN=ORM1 PE=2 SV=1 |
| P41361 | 16 | 16 | 820.34 | 7.06E-05 | 5.24E-05 | 2.424113 | ANT3_BOVIN Antithrombin-III OS=Bos taurus GN=SERPINC1 PE=1 SV=2 |
| Q3SZV7 | 15 | 15 | 610.22 | 7.26E-05 | 5.35E-05 | 2.385303 | HEMO_BOVIN Hemopexin OS=Bos taurus GN=HPX PE=2 SV=1 |
| Q3MHN2 | 19 | 19 | 984.98 | 7.49E-05 | 5.48E-05 | 2.348719 | CO9_BOVIN Complement component C9 OS=Bos taurus GN=C9 PE=2 SV=1 |
| Q5E9B7 | 9 | 8 | 453.19 | 8.20E-05 | 5.74E-05 | 2.593321 | CLIC1_BOVIN Chloride intracellular channel protein 1 OS=Bos taurus GN=CLIC1 PE=2 SV=3 |
| Q5NTB3 | 2 | 2 | 55.81 | 8.28E-05 | 5.75E-05 | 3.187231 | FA11_BOVIN Coagulation factor XI OS=Bos taurus GN=F11 PE=1 SV=1 |
| Q32PJ2 | 24 | 24 | 1439.7 | 0.000107 | 6.79E-05 | 2.37678 | APOA4_BOVIN Apolipoprotein A-IV OS=Bos taurus GN=APOA4 PE=2 SV=1 |
| P00978 | 13 | 13 | 775.67 | 0.000108 | 6.79E-05 | 2.306472 | AMBP_BOVIN Protein AMBP OS=Bos taurus GN=AMBP PE=1 SV=2 |
| Q28178 | 41 | 41 | 1862.11 | 0.000108 | 6.80E-05 | 2.336451 | TSP1_BOVIN Thrombospondin-1 OS=Bos taurus GN=THBS1 PE=2 SV=2 |
| P28800 | 12 | 12 | 539.67 | 0.000114 | 7.05E-05 | 2.363687 | A2AP_BOVIN Alpha-2-antiplasmin OS=Bos taurus GN=SERPINF2 PE=1 SV=2 |
| P02070 | 13 | 9 | 1182.02 | 0.000122 | 7.49E-05 | 2.577669 | HBB_BOVIN Hemoglobin subunit beta OS=Bos taurus GN=HBB PE=1 SV=1 |
| Q9TT36 | 16 | 15 | 760.44 | 0.000136 | 7.99E-05 | 2.189601 | THBG_BOVIN Thyroxine-binding globulin OS=Bos taurus GN=SERPINA7 PE=2 SV=1 |
| P37141 | 7 | 7 | 284.5 | 0.000145 | 8.35E-05 | 2.211697 | GPX3_BOVIN Glutathione peroxidase 3 OS=Bos taurus GN=GPX3 PE=2 SV=2 |
| Q3Y5Z3 | 5 | 5 | 290.45 | 0.000166 | 9.06E-05 | 3.627153 | ADIPO_BOVIN Adiponectin OS=Bos taurus GN=ADIPOQ PE=1 SV=1 |
| P34955 | 29 | 29 | 1876.95 | 0.000177 | 9.44E-05 | 2.64792 | A1AT_BOVIN Alpha-1-antiproteinase OS=Bos taurus GN=SERPINA1 PE=1 SV=1 |
| P17697 | 16 | 14 | 913.47 | 0.000185 | 9.83E-05 | 2.176927 | CLUS_BOVIN Clusterin OS=Bos taurus GN=CLU PE=1 SV=1 |
| P81187 | 30 | 30 | 1729.64 | 0.00019 | 9.96E-05 | 2.057717 | CFAB_BOVIN Complement factor B OS=Bos taurus GN=CFB PE=1 SV=2 |
| P35445 | 15 | 13 | 728.5 | 0.000214 | 0.000108 | 2.491474 | COMP_BOVIN Cartilage oligomeric matrix protein OS=Bos taurus GN=COMP PE=1 SV=2 |
| P19035 | 4 | 4 | 334.6 | 0.000214 | 0.000108 | 3.660115 | APOC3_BOVIN Apolipoprotein C-III OS=Bos taurus GN=APOC3 PE=1 SV=2 |
| Q58D62 | 13 | 13 | 830.57 | 0.000224 | 0.000112 | 2.322888 | FETUB_BOVIN Fetuin-B OS=Bos taurus GN=FETUB PE=1 SV=1 |
| P02672 | 20 | 19 | 1137.56 | 0.000235 | 0.000116 | 2.786274 | FIBA_BOVIN Fibrinogen alpha chain OS=Bos taurus GN=FGA PE=1 SV=5 |
| E1BF81 | 10 | 10 | 634.35 | 0.000263 | 0.000126 | 2.050833 | CBG_BOVIN Corticosteroid-binding globulin OS=Bos taurus GN=SERPINA6 PE=3 SV=1 |
| Q7SIH1 | 95 | 90 | 7701.76 | 0.000285 | 0.000134 | 2.740499 | A2MG_BOVIN Alpha-2-macroglobulin OS=Bos taurus GN=A2M PE=1 SV=2 |
| P02769 | 54 | 53 | 4902.73 | 0.000284 | 0.000134 | 3.223403 | ALBU_BOVIN Serum albumin OS=Bos taurus GN=ALB PE=1 SV=4 |
| P15497 | 21 | 20 | 1277.67 | 0.000295 | 0.000137 | 2.486157 | APOA1_BOVIN Apolipoprotein A-I OS=Bos taurus GN=APOA1 PE=1 SV=3 |
| P80425 | 2 | 2 | 75.36 | 0.000299 | 0.000138 | 3.016269 | FABPL_BOVIN Fatty acid-binding protein, liver OS=Bos taurus GN=FABP1 PE=1 SV=1 |
| Q3SZZ7 | 2 | 2 | 54.55 | 0.000333 | 0.000151 | 2.379136 | FGL1_BOVIN Fibrinogen-like protein 1 OS=Bos taurus GN=FGL1 PE=2 SV=1 |
| Q32PA5 | 3 | 3 | 105.47 | 0.000344 | 0.000156 | 2.65951 | UBE2C_BOVIN Ubiquitin-conjugating enzyme E2 C OS=Bos taurus GN=UBE2C PE=2 SV=1 |
| P19879 | 3 | 3 | 77 | 0.000353 | 0.000158 | 2.046722 | MIME_BOVIN Mimecan OS=Bos taurus GN=OGN PE=1 SV=2 |
| Q03247 | 20 | 20 | 1361.34 | 0.000366 | 0.000164 | 2.341082 | APOE_BOVIN Apolipoprotein E OS=Bos taurus GN=APOE PE=1 SV=1 |
| P01044 | 23 | 5 | 973.49 | 0.00037 | 0.000165 | 3.283213 | KNG1_BOVIN Kininogen-1 OS=Bos taurus GN=KNG1 PE=1 SV=1 |
| Q6URK6 | 7 | 7 | 279.5 | 0.000426 | 0.000186 | 2.900556 | CADH5_BOVIN Cadherin-5 OS=Bos taurus GN=CDH5 PE=2 SV=1 |
| P56652 | 34 | 34 | 1799.93 | 0.000456 | 0.000196 | 2.205935 | ITIH3_BOVIN Inter-alpha-trypsin inhibitor heavy chain H3 OS=Bos taurus GN=ITIH3 PE=1 SV=2 |
| Q2KJF1 | 15 | 14 | 1113.64 | 0.000471 | 0.000201 | 2.180778 | A1BG_BOVIN Alpha-1B-glycoprotein OS=Bos taurus GN=A1BG PE=1 SV=1 |
| P12799 | 13 | 13 | 564.41 | 0.000498 | 0.000211 | 2.10633 | FIBG_BOVIN Fibrinogen gamma-B chain OS=Bos taurus GN=FGG PE=1 SV=1 |
| P12763 | 15 | 15 | 1635.28 | 0.000713 | 0.000282 | 2.808216 | FETUA_BOVIN Alpha-2-HS-glycoprotein OS=Bos taurus GN=AHSG PE=1 SV=2 |
| P07224 | 10 | 10 | 371.84 | 0.000732 | 0.000288 | 2.399418 | PROS_BOVIN Vitamin K-dependent protein S OS=Bos taurus GN=PROS1 PE=1 SV=1 |
| Q3T0Y8 | 2 | 2 | 62.88 | 0.000736 | 0.000289 | 2.018389 | VAMP8_BOVIN Vesicle-associated membrane protein 8 OS=Bos taurus GN=VAMP8 PE=3 SV=1 |
| Q0VCX1 | 6 | 6 | 208.05 | 0.000751 | 0.000293 | 2.996765 | C1S_BOVIN Complement C1s subcomponent OS=Bos taurus GN=C1S PE=2 SV=2 |
| Q2KJ39 | 3 | 3 | 137.62 | 0.000793 | 0.000306 | 2.914673 | RCN3_BOVIN Reticulocalbin-3 OS=Bos taurus GN=RCN3 PE=2 SV=1 |
| Q28085 | 17 | 16 | 649.42 | 0.000845 | 0.000321 | 2.227007 | CFAH_BOVIN Complement factor H OS=Bos taurus GN=CFH PE=1 SV=3 |
| P01030 | 18 | 17 | 979.76 | 0.000962 | 0.000357 | 2.478328 | CO4_BOVIN Complement C4 (Fragments) OS=Bos taurus GN=C4 PE=1 SV=2 |
| Q24K22 | 4 | 3 | 86.54 | 0.001002 | 0.00037 | 2.459067 | HGFL_BOVIN Hepatocyte growth factor-like protein OS=Bos taurus GN=MST1 PE=2 SV=1 |
| A2I7N1 | 13 | 2 | 427.04 | 0.001077 | 0.00039 | 2.794911 | SPA35_BOVIN Serpin A3-5 OS=Bos taurus GN=SERPINA3-5 PE=3 SV=1 |
| P02676 | 17 | 17 | 773.27 | 0.001155 | 0.000411 | 2.412305 | FIBB_BOVIN Fibrinogen beta chain OS=Bos taurus GN=FGB PE=1 SV=2 |
| Q9TTE1 | 15 | 2 | 862.91 | 0.00116 | 0.000411 | 3.622459 | SPA31_BOVIN Serpin A3-1 OS=Bos taurus GN=SERPINA3-1 PE=1 SV=3 |
| A0JN69 | 2 | 2 | 52.13 | 0.001167 | 0.000413 | 2.546956 | MARH3_BOVIN E3 ubiquitin-protein ligase MARCH3 OS=Bos taurus GN=MARCH3 PE=2 SV=1 |
| Q95M17 | 9 | 9 | 413.41 | 0.0012 | 0.00042 | 2.13394 | CHIA_BOVIN Acidic mammalian chitinase OS=Bos taurus GN=CHIA PE=1 SV=1 |
| P30932 | 4 | 4 | 223.13 | 0.001249 | 0.000435 | 2.124227 | CD9_BOVIN CD9 antigen OS=Bos taurus GN=CD9 PE=2 SV=2 |
| Q32KY0 | 5 | 4 | 176.03 | 0.001306 | 0.000451 | 2.166479 | APOD_BOVIN Apolipoprotein D OS=Bos taurus GN=APOD PE=2 SV=1 |
| Q3SZ57 | 26 | 25 | 1140.62 | 0.001343 | 0.00046 | 2.322679 | FETA_BOVIN Alpha-fetoprotein OS=Bos taurus GN=AFP PE=2 SV=1 |
| Q2KIT0 | 3 | 3 | 120.43 | 0.001353 | 0.000462 | 3.573179 | HP20_BOVIN Protein HP-20 homolog OS=Bos taurus PE=2 SV=1 |
| Q9TTJ5 | 3 | 3 | 109.82 | 0.001408 | 0.00048 | 3.329858 | RGN_BOVIN Regucalcin OS=Bos taurus GN=RGN PE=2 SV=1 |
| P24591 | 3 | 3 | 82.55 | 0.001425 | 0.000484 | 2.022075 | IBP1_BOVIN Insulin-like growth factor-binding protein 1 OS=Bos taurus GN=IGFBP1 PE=2 SV=2 |
| P50448 | 10 | 10 | 593.67 | 0.001431 | 0.000486 | 2.077351 | F12AI_BOVIN Factor XIIa inhibitor OS=Bos taurus PE=1 SV=1 |
| Q3SYU6 | 3 | 3 | 83.8 | 0.001542 | 0.000518 | 2.077923 | CNN2_BOVIN Calponin-2 OS=Bos taurus GN=CNN2 PE=2 SV=3 |
| Q28106 | 3 | 3 | 84.33 | 0.00157 | 0.000524 | 2.729686 | CNTN1_BOVIN Contactin-1 OS=Bos taurus GN=CNTN1 PE=2 SV=1 |
| A2I7N3 | 7 | 5 | 345.92 | 0.001671 | 0.000552 | 2.593972 | SPA37_BOVIN Serpin A3-7 OS=Bos taurus GN=SERPINA3-7 PE=3 SV=1 |
| P81644 | 4 | 4 | 290.47 | 0.002112 | 0.000675 | 2.745647 | APOA2_BOVIN Apolipoprotein A-II OS=Bos taurus GN=APOA2 PE=1 SV=2 |
| Q58CQ9 | 2 | 2 | 44.65 | 0.003265 | 0.000971 | 3.567331 | VNN1_BOVIN Pantetheinase OS=Bos taurus GN=VNN1 PE=1 SV=1 |
| P35541 | 3 | 3 | 144.74 | 0.004955 | 0.001408 | 2.89858 | SAA_BOVIN Serum amyloid A protein OS=Bos taurus GN=SAA1 PE=1 SV=2 |
| Q2KIS7 | 9 | 9 | 635.62 | 0.005719 | 0.001606 | 2.78106 | TETN_BOVIN Tetranectin OS=Bos taurus GN=CLEC3B PE=2 SV=1 |
| Q3T0A3 | 2 | 2 | 57.63 | 0.005786 | 0.00162 | 2.266021 | CFAD_BOVIN Complement factor D OS=Bos taurus GN=CFD PE=2 SV=1 |
| P04815 | 3 | 3 | 153.07 | 0.005831 | 0.00163 | 3.031992 | BPT2_BOVIN Spleen trypsin inhibitor I OS=Bos taurus PE=1 SV=2 |
| P02754 | 3 | 2 | 114.56 | 0.006938 | 0.001882 | 3.825642 | LACB_BOVIN Beta-lactoglobulin OS=Bos taurus GN=LGB PE=1 SV=3 |
| O77783 | 2 | 2 | 48.25 | 0.008468 | 0.002242 | 2.742601 | EXT2_BOVIN Exostosin-2 OS=Bos taurus GN=EXT2 PE=1 SV=1 |
| Q27967 | 5 | 5 | 297.27 | 0.017094 | 0.004202 | 2.206484 | SPP24_BOVIN Secreted phosphoprotein 24 OS=Bos taurus GN=SPP2 PE=1 SV=2 |
| Q5XQN5 | 18 | 10 | 823.36 | 0.021123 | 0.005104 | 2.976525 | K2C5_BOVIN Keratin, type II cytoskeletal 5 OS=Bos taurus GN=KRT5 PE=1 SV=1 |
| Q148H6 | 4 | 2 | 244.26 | 0.03551 | 0.008166 | 3.974888 | K1C28_BOVIN Keratin, type I cytoskeletal 28 OS=Bos taurus GN=KRT28 PE=2 SV=1 |
| P06394 | 11 | 7 | 573.62 | 0.044624 | 0.010039 | 3.888336 | K1C10_BOVIN Keratin, type I cytoskeletal 10 OS=Bos taurus GN=KRT10 PE=3 SV=1 |

**S4 Table. Structure of *miR-181a* and *b.*** *bta-miR-181a and bta-miR-181b* sequences were obtained from miRBase 21. *bta-miR-181a and bta-miR-181b* secondary structure was predicted by folding and hybridization software Mfold (http://unafold.rna.albany.edu )

| **Species and miRNA** | **Co-ords** | **Sequence (Mature miRNA in uppercase, seed underlined)** | **Structure (using mfold http://mfold.rna.albany.edu/)** | **Initial ΔG** |
| --- | --- | --- | --- | --- |
| Bos taurus mir-181a-1 | chr16: 79940482-79940591 [-] | tgagctccgaggttgcttcagtgAACATTCAACGCTGTCGGTGAGTTtggaattaaaaatcaaaaccatcgaccgttgattgtaccctatggccaaccaccatctccacc | 10 20 30 40 50  u---\| cuccga - uc ugA U CU A g auu  gag gguug cu ag ACA UCAACG GUCGGUG GUUu ga a  cuc ccaac gg uc ugu aguugc cagcuac caaa cu a  ccac^ uacca- c ua cca u -- - a aaa  . 100 90 80 70 60 | -31.70 |
| Bos taurus mir-181b-1 | chr16: 79940310-79940419 [-] | cttgggcagaggttctttcttaaaaggtcacaatcAACATTCATTGCTGTCGGTGGGTTgaactgtgtggacaagctcactgaacaatgagtgcaactgtggccccgcat | 10  cuugg .-a\| uu  gc gagg \  cg uucu c  ua--- \ -^ uu  . 20  30 40 50 60  aaaa aucAA CUG gaa g  ggucaca CAUUCAUUG UCGGUGGGUU cu u  ccggugu gugaguaac agucacucga gg g  cc-- caac- a-- aca u  100 90 80 70 | -36.00 |

**S5 Table. List of four most significant pathways corresponding to *miR-181a* targets.**

| Ingenuity Canonical Pathways | -log(p-value) | Ratio | Molecules |
| --- | --- | --- | --- |
| Ceramide Degradation | 2.79 | 0.429 | ASAH2B,ACER2,ASAH2 |
| PTEN Signaling | 2.77 | 0.101 | PIK3R3,TGFBR2,RPS6KB1, CSNK2A2,TGFBR1,FLT1,KRAS,CDKN1B,MAP2K1,BCL2L11,FASLG,BCL2 |
| Antiproliferative Role of TOB in T Cell Signaling | 2.6 | 0.192 | TGFBR2,RB1,TGFBR1,IL2,CDKN1B |
| Regulation of IL-2 Expression in Activated and Anergic T Lymphocytes | 2.52 | 0.112 | TGFBR2,CALM1 (includes others),TGFBR1,IL2,CARD11,PPP3R1,KRAS,MAP2K1,TRA |

**S6 Table** – **Cross-referencing of gene lists**. List of 44 genes which are present in the IPA generated miR-181a predicted target list and are up-regulated in TBL20 cells (microarray data from Kinnaird *et al*, 2013b)

| Gene | Function (from NCBI GENE description) | | | | | |
| --- | --- | --- | --- | --- | --- | --- |
| ACTA2 | The protein encoded by this gene belongs to the actin family of proteins, which are highly conserved proteins that play a role in cell motility, structure and integrity. Alpha, beta and gamma actin isoforms have been identified, with alpha actins being a major constituent of the contractile apparatus, while beta and gamma actins are involved in the regulation of cell motility. This actin is an alpha actin that is found in skeletal muscle. | | | | | |
| ARHGAP18 | ARHGAP18 belongs to a family of Rho (see MIM 165390) GTPase-activating proteins that modulate cell signaling | | | | | |
| ATP6V1C2 | his gene encodes a component of vacuolar ATPase (V-ATPase), a multisubunit enzyme that mediates acidification of eukaryotic intracellular organelles. V-ATPase dependent organelle acidification is necessary for such intracellular processes as protein sorting, zymogen activation, receptor-mediated endocytosis, and synaptic vesicle proton gradient generation. V-ATPase is composed of a cytosolic V1 domain and a transmembrane V0 domain. The V1 domain consists of three A,three B, and two G subunits, as well as a C, D, E, F, and H subunit. The V1 domain contains the ATP catalytic site. This gene encodes alternate transcriptional splice variants, encoding different V1 domain C subunit isoforms. | | | | | |
| BAG2 | BAG proteins compete with Hip for binding to the Hsc70/Hsp70 ATPase domain and promote substrate release. All the BAG proteins have an approximately 45-amino acid BAG domain near the C terminus but differ markedly in their N-terminal regions. The predicted BAG2 protein contains 211 amino acids. The BAG domains of BAG1, BAG2, and BAG3 interact specifically with the Hsc70 ATPase domain in vitro and in mammalian cells. All 3 proteins bind with high affinity to the ATPase domain of Hsc70 and inhibit its chaperone activity in a Hip-repressible manner. | | | | | |
| BHLHE40 | This gene encodes a basic helix-loop-helix protein expressed in various tissues. The encoded protein can interact with ARNTL or compete for E-box binding sites in the promoter of PER1 and repress CLOCK/ARNTL's transactivation of PER1. This gene is believed to be involved in the control of circadian rhythm and cell differentiation. | | | | | |
| CAMKK1 | The product of this gene belongs to the Serine/Threonine protein kinase family, and to the Ca(2+)/calmodulin-dependent protein kinase subfamily. This protein plays a role in the calcium/calmodulin-dependent (CaM) kinase cascade. Three transcript variants encoding two distinct isoforms have been identified for this gene. | | | | | |
| CBLB | This gene encodes an E3 ubiquitin-protein ligase which promotes proteosome-mediated protein degradation by transferring ubiquitin from an E2 ubiquitin-conjugating enzyme to a substrate. The encoded protein is involved in the regulation of immune response by limiting T-cell receptor, B-cell receptor, and high affinity immunoglobulin epsilon receptor activation. Studies in mouse suggest that this gene is involved in antifungal host defense and that its inhibition leads to increased fungal killing. Manipulation of this gene may be beneficial in implementing immunotherapies for a variety of conditions, including cancer, autoimmune diseases, allergies, and infections. | | | | | |
| CBX7 | This gene encodes a protein that contains the CHROMO (CHRomatin Organization MOdifier) domain. The encoded protein is a component of the Polycomb repressive complex 1 (PRC1), and is thought to control the lifespan of several normal human cells. | | | | | |
| CD69 | This gene encodes a member of the calcium dependent lectin superfamily of type II transmembrane receptors. Expression of the encoded protein is induced upon activation of T lymphocytes, and may play a role in proliferation. Furthermore, the protein may act to transmit signals in natural killer cells and platelets. [ | | | | | |
| CPOX | The protein encoded by this gene is the sixth enzyme of the heme biosynthetic pathway. The encoded enzyme is soluble and found in the intermembrane space of mitochondria. This enzyme catalyzes the stepwise oxidative decarboxylation of coproporphyrinogen III to protoporphyrinogen IX, a precursor of heme. Defects in this gene are a cause of hereditary coproporphyria (HCP). | | | | | |
| DDIT4 | This gene encodes the DNA damage inducible transcript 4 | | | | | |
| DERL1 | he protein encoded by this gene is a member of the derlin family. Members of this family participate in the ER-associated degradation response and retrotranslocate misfolded or unfolded proteins from the ER lumen to the cytosol for proteasomal degradation. This protein recognizes substrate in the ER and works in a complex to retrotranslocate it across the ER membrane into the cytosol. This protein may select cystic fibrosis transmembrane conductance regulator protein (CFTR) for degradation as well as unfolded proteins in Alzheimer's disease. Alternative splicing results in multiple transcript variants that encode different protein isoforms. | | | | | |
| DUSP10 | Dual specificity protein phosphatases inactivate their target kinases by dephosphorylating both the phosphoserine/threonine and phosphotyrosine residues. They negatively regulate members of the MAP kinase superfamily, which is associated with cellular proliferation and differentiation. Different members of this family of dual specificity phosphatases show distinct substrate specificities for MAP kinases, different tissue distribution and subcellular localization, and different modes of expression induction by extracellular stimuli. This gene product binds to and inactivates p38 and SAPK/JNK. Alternative splicing results in multiple transcript variants | | | | | |
| DUSP6 | The protein encoded by this gene is a member of the dual specificity protein phosphatase subfamily. These phosphatases inactivate their target kinases by dephosphorylating both the phosphoserine/threonine and phosphotyrosine residues. They negatively regulate members of the mitogen-activated protein (MAP) kinase superfamily (MAPK/ERK, SAPK/JNK, p38), which are associated with cellular proliferation and differentiation. Different members of the family of dual specificity phosphatases show distinct substrate specificities for various MAP kinases, different tissue distribution and subcellular localization, and different modes of inducibility of their expression by extracellular stimuli. This gene product inactivates ERK2, is expressed in a variety of tissues with the highest levels in heart and pancreas, and unlike most other members of this family, is localized in the cytoplasm. Mutations in this gene have been associated with congenital hypogonadotropic hypogonadism. Alternatively spliced transcript variants have been found for this gene. [ | | | | | |
| EIF4A2 | Encodes eukaryotic translation initiation factor 4A2 | | | | |  |
| EXOSC3 | This gene encodes a non-catalytic component of the human exosome, a complex with 3'-5' exoribonuclease activity that plays a role in numerous RNA processing and degradation activities. Related pseudogenes of this gene are found on chromosome 19 and 21. Alternatively spliced transcript variants encoding different isoforms have been described. | | | | | |
| FAS | The protein encoded by this gene is a member of the TNF-receptor superfamily. This receptor contains a death domain. It has been shown to play a central role in the physiological regulation of programmed cell death, and has been implicated in the pathogenesis of various malignancies and diseases of the immune system. The interaction of this receptor with its ligand allows the formation of a death-inducing signaling complex that includes Fas-associated death domain protein (FADD), caspase 8, and caspase 10. The autoproteolytic processing of the caspases in the complex triggers a downstream caspase cascade, and leads to apoptosis. This receptor has been also shown to activate NF-kappaB, MAPK3/ERK1, and MAPK8/JNK, and is found to be involved in transducing the proliferating signals in normal diploid fibroblast and T cells. Several alternatively spliced transcript variants have been described, some of which are candidates for nonsense-mediated mRNA decay (NMD). The isoforms lacking the transmembrane domain may negatively regulate the apoptosis mediated by the full length isoform. | | | | | |
| GTPBP3 | This locus encodes a GTP-binding protein. The encoded protein is localized to the mitochondria and may play a role in mitochondrial tRNA modification. Polymorphisms at this locus may be associated with severity of aminoglycoside-induced deafness, a disease associated with a mutation in the 12S rRNA. Alternatively spliced transcript variants encoding different isoforms have been described. [ | | | | | |
| HIGD2A | The protein encoded by this gene is a subunit of the cytochrome c oxidase complex (complex IV), which is the terminal enzyme in the mitochondrial respiratory chain. The encoded protein is an inner mitochondrial membrane protein and is a functional ortholog of the yeast respiratory supercomplex factor 1 (Rcf1). In mouse, the orthologous protein enhances cell survival under conditions of hypoxia. | | | | | |
| HOXB8 | This gene is a member of the Antp homeobox family and encodes a nuclear protein with a homeobox DNA-binding domain. It is included in a cluster of homeobox B genes located on chromosome 17. The encoded protein functions as a sequence-specific transcription factor that is involved in development. Increased expression of this gene is associated with colorectal cancer. Mice that have had the murine ortholog of this gene knocked out exhibit an excessive pathologic grooming behavior. This behavior is similar to the behavior of humans suffering from the obsessive-compulsive spectrum disorder trichotillomania. | | | | | |
| HSP90B1 | HSP90B1 |  |  |  |  |  |
| IPPK | The protein encoded by this gene is a kinase that phosphorylates position 2 of inositol-1,3,4,5,6-pentakisphosphate to form inositol-1,2,3,4,5,6-hexakisphosphate (InsP6). InsP6 has a variety of functions, including stimulation of DNA repair, endocytosis, and mRNA export. | | | | | |
| MAGEB3 | This gene is a MAGE-B subfamily member of the MAGE gene family. MAGE family member proteins direct the expression of tumor antigens recognized on a human melanoma by autologous cytolytic T lymphocytes. There are two known clusters of MAGE genes on chromosome X. The members of the MAGE-A subfamily are located in the Xq28 region, while the members of the MAGE-B subfamily are clustered in the Xp21 region. | | | | | |
| MBOAT1 | This gene belongs to the membrane-bound O-acetyltransferase superfamily. The encoded transmembrane protein is an enzyme that transfers organic compounds, preferably from oleoyl-CoA, to hydroxyl groups of protein targets in membranes. A translocation disrupting this gene may be associated with brachydactyly syndactyly syndrome. Alternately spliced transcript variants have been described for this gene. [ | | | | | |
| MMP14 | Proteins of the matrix metalloproteinase (MMP) family are involved in the breakdown of extracellular matrix in normal physiological processes, such as embryonic development, reproduction, and tissue remodeling, as well as in disease processes, such as arthritis and metastasis. Most MMP's are secreted as inactive proproteins which are activated when cleaved by extracellular proteinases. However, the protein encoded by this gene is a member of the membrane-type MMP (MT-MMP) subfamily; each member of this subfamily contains a potential transmembrane domain suggesting that these proteins are expressed at the cell surface rather than secreted. This protein activates MMP2 protein, and this activity may be involved in tumor invasion. | | | | | |
| MPP7 | The protein encoded by this gene is a member of the p55 Stardust family of membrane-associated guanylate kinase (MAGUK) proteins, which function in the establishment of epithelial cell polarity. This family member forms a complex with the polarity protein DLG1 (discs, large homolog 1) and facilitates epithelial cell polarity and tight junction formation. Polymorphisms in this gene are associated with variations in site-specific bone mineral density (BMD). Alternative splicing results in multiple transcript variants. | | | | | |
| MPZL3 | Encodes myelin protein zero-like 3 | | |  |  |  |
| NKAIN2 | This gene encodes a transmembrane protein that interacts with the beta subunit of a sodium/potassium-transporting ATPase. A chromosomal translocation involving this gene is a cause of lymphoma. Alternative splicing results in multiple transcript variants encoding distinct isoforms. | | | | | |
| PAM | This gene encodes a multifunctional protein. The encoded preproprotein is proteolytically processed to generate the mature enzyme. This enzyme includes two domains with distinct catalytic activities, a peptidylglycine alpha-hydroxylating monooxygenase (PHM) domain and a peptidyl-alpha-hydroxyglycine alpha-amidating lyase (PAL) domain. These catalytic domains work sequentially to catalyze the conversion of neuroendocrine peptides to active alpha-amidated products. Alternative splicing results in multiple transcript variants, at least one of which encodes an isoform that is proteolytically processed. | | | | | |
| PARM1 | Encodes prostate androgen-regulated mucin-like protein 1 | | | | | |
| PAX9 | This gene is a member of the paired box (PAX) family of transcription factors. Members of this gene family typically contain a paired box domain, an octapeptide, and a paired-type homeodomain. These genes play critical roles during fetal development and cancer growth. Mice lacking this gene exhibit impaired development of organs, musculature and the skeleton, including absent and abnormally developed teeth, and neonatal lethality. Mutations in the human gene are associated with selective tooth agenesis-3. | | | | | |
| PCDHA13 | This gene is a member of the protocadherin alpha gene cluster, one of three related gene clusters tandemly linked on chromosome five that demonstrate an unusual genomic organization similar to that of B-cell and T-cell receptor gene clusters. The alpha gene cluster is composed of 15 cadherin superfamily genes related to the mouse CNR genes and consists of 13 highly similar and 2 more distantly related coding sequences. The tandem array of 15 N-terminal exons, or variable exons, are followed by downstream C-terminal exons, or constant exons, which are shared by all genes in the cluster. The large, uninterrupted N-terminal exons each encode six cadherin ectodomains while the C-terminal exons encode the cytoplasmic domain. These neural cadherin-like cell adhesion proteins are integral plasma membrane proteins that most likely play a critical role in the establishment and function of specific cell-cell connections in the brain. Alternative splicing has been observed and additional variants have been suggested but their full-length nature has yet to be determined. | | | | | |
| PITPNB | This gene encodes a cytoplasmic protein that catalyzes the transfer of phosphatidylinositol and phosphatidylcholine between membranes. This transfer activity is required for COPI complex-mediated retrograde transport from the Golgi apparatus to the endoplasmic reticulum. Alternative splicing of this gene results in multiple transcript variants. | | | | | |
| POMC | This gene encodes a polypeptide hormone precursor that undergoes extensive, tissue-specific, post-translational processing. Processing yields several biologically active peptides, which are involved in diverse cellular functions, such as energy homeostasis, steroidogenesis, and increased melanin production in melanocytes. In mouse deficiency of this gene is associated with obesity, defects in adrenal development, and altered pigmentation. A pseudogene of this gene is located on chromosome 19. Alternative splicing results in multiple transcript variants. | | | | | |
| PQLC1 | Encodes a PQ loop repeat containing 1 | | | |  |  |
| PRKCD | The protein encoded by this gene is a member of the protein kinase C family of serine- and threonine-specific protein kinases. The encoded protein is activated by diacylglycerol and is both a tumor suppressor and a positive regulator of cell cycle progression. Also, this protein can positively or negatively regulate apoptosis. Defects in this gene are a cause of autoimmune lymphoproliferative syndrome. | | | | | |
| RAP2A | Encodes a member of RAS oncogene family | | | |  |  |
| RBM47 | This gene encodes RNA binding motif protein 47 | | | | |  |
| RCAN3 | This gene encodes regulator of calcineurin 3 | | | |  |  |
| SLA | This gene encodes an Src like adaptor | | | |  |  |
| SLC2A3 | Encodes a solute carrier family 2 member 3 | | | |  |  |
| ZADH2 | Encodes zinc binding alcohol dehydrogenase, domain containing 2 | | | | | |
| ZDHHC3 | Encodes the zinc finger, DHHC domain containing 3 | | | | |  |
| ZFP28 | Encodes zinc finger protein 28pr | | |  |  |  |
